# Supplementary material for: Developing an intervention to improve early infant HIV diagnosis service uptake among postpartum women in Malawi’s primary healthcare using a co-designing approach with stakeholders
Source: PLOS Glob Public Health. 2025 Apr 22;5(4):e0004426. doi: 10.1371/journal.pgph.0004426 (PMC12013899; doi:10.1371/journal.pgph.0004426)
Supplement: S1 Text — (DOCX) [file pgph.0004426.s001.docx]

Inclusivity in global research

PLOS’ policy on inclusivity in global research aims to improve transparency in the reporting of research performed outside of researchers’ own country or community and ensures that PLOS publications reporting global research adhere to high standards for research ethics and authorship. Authors of relevant research articles may be asked to complete the questionnaire below, which outlines ethical, cultural, and scientific considerations specific to inclusivity in global research. This questionnaire may be requested when researchers have travelled to a different country to conduct research, if research uses samples collected in another country, research with Indigenous populations or their lands, or if research is on cultural artefacts. Researchers travelling to another country solely to use laboratory equipment will not normally be required to complete the questionnaire. However, the questionnaire can be requested at the journal’s discretion for any submission – if you have been requested to complete this questionnaire by the PLOS journal you submitted to, please do so.

Please complete the questionnaire below and include this as a Supporting Information file with your manuscript. Note that if your paper is accepted for publication, this checklist will be published with your article in the supporting information files. Please ensure that you reference the checklist in the main body of your manuscript. We suggest adding a subsection ‘Inclusivity in global research’ to your Methods section and adding the following sentence: “Additional information regarding the ethical, cultural, and scientific considerations specific to inclusivity in global research is included in the Supporting Information (SX Checklist)”

The questions have been designed to be applicable to a wide range of study types, and there are subsections for both human subjects research and non-human subjects’ research. If any of the questions are not relevant to your research, please mark them as “N/A” as appropriate.

**Ethical considerations, permits and authorship**

*This section is applicable to all research types.*

Provide details as to who granted permissions and/or consent for the study to take place in the Methods section of your manuscript. This should include the names of **all** ethics boards, governmental organizations, community leaders or other bodies that provided approval for the study. If individuals provided approval refer to these people by their role or title but do not list their name(s).

Reported on page number: Page 6

1. College of Medicine Research & Ethics Committee (P.04/22/3607) and the Liverpool School of Tropical Medicine Research Ethics Committee (22-025).

2. Blantyre district health management and research committee

3. Director of Health and Social Services in Blantyre district

4. Respective Health facilities management team members and representatives from the community for the health advisory committee

5. Different non-governmental organisation (EGPAF, Umunthu, John Hopkins, Malawi Liverpool Welcome Trust, Macro)

If there were any deviations from the study protocol after approval was obtained, please provide details of these changes in the Methods section of your manuscript.
Did this study involve local collaborators that are residents of the country where the research was conducted, or members of the community studied? If you do not have any authors from said communities, please provide an explanation for this below.

No

Reported on page number: N/A

1. Yes, the study involved collaborators from the country (Augustine Choko ^1,2^, Linda Alinane Nyondo-Mipando ^5, 6^) and some co-authors are part of the stakeholders involved in the study (Jenifer Hezekiah Zimba ^7^, Edda Lipipa ^7^, Dorcus Nothale^7^, Afunawo Mdala^8^ Joe Nkhonjera ^9^, Melody Sakala^3^,)

2. The principal researcher is from the country

Everyone listed as an author should meet PLOS’ criteria for authorship and all individuals who meet these criteria should be included in the author byline, rather than the acknowledgements. For further information please see the journal’s Authorship Policy. (Yes, they do)

**Human subjects research (e.g. health research, medical research, cross-cultural psychology)**

Did you obtain written informed consent from a representative of the local community or region before the research took place? How did you establish who speaks for the community? Details of written informed consent obtained from study participants should be reported separately in the Methods section of your manuscript.

1. Before the study, we made a research presentation to the Blantyre district health management team and research committee, and we later received approval to conduct the study.
2. We made a presentation to the primary facilities, including the management teams for the facilities, who also invited community representatives who speak on behalf of the community as members of health advisory committees
3. After stakeholder mapping led by the Blantyre district managers, the director of health and social services approved later for different healthcare workers' stakeholders to attend workshops
4. We contacted non-governmental organization stakeholders who appointed themselves people to represent them at workshops.

How did members of the local community provide input on the aims of the research investigation, its methodology, and its anticipated outcome(s)?

We held workshops and had small homogenous group discussions to facilitate people to speak freely within small groups of people with similar characteristics. They were only given topics to discuss and were free to talk in line with their experiences and knowledge. And used flip charts to document what they could share with the group. Moderators walked around to check if they had any questions and how they were getting along with the discussion. A representative shared These discussions with the larger group of members and were open to the entire group. At the end of the workshops, these were summarised, and members confirmed what they reported. Their flip charts were also used to consolidate their inputs. There were two series of workshops, and at the second workshop, the session started with members validating what they shared in the previous session.

Some coauthors who were part of the engagement team also reviewed and validated all analysed findings. The entire community of healthcare workers further validated these findings in two study sites during training of proposed solutions in subsequent phases of the study.

When engaging with the local community, how did you ensure that the informed consent documents and other materials could be understood by local stakeholders?

Information sheets about the study were given to stakeholders mapped through the program managers, and two days later, the researcher followed up by phone with participants. Those who were interested in attending the workshops opted in. The workshop agenda and other supporting documents, including workshop logistics, were shared in advance for an average of one to two weeks. Participants were free to ask the researcher anything during this period. The workshops took place at a neutral place, and those who understood and were willing came to the workshop venue.

Will the findings of the research be made available in an understandable format to stakeholders in the community where the study was conducted (e.g. via a presentation, summary report, copies of publications, etc.)? Please provide details of how this will be achieved.

Yes, for example, these findings were already summarised and validated during the training of HCW at two study sites.

This manuscript has been the work of researchers and coauthors who are part of the community, and it has been circulated, getting their input and feedback.

However, overall findings will further be disseminated at the district health office, engaging the NGO and key healthcare workers from the facilities and later at the primary facilities involving the community representatives as well

**Non-human subjects research using specimens/ animals collected as part of the study, or those housed in archival collections. Examples include archaeology, paleontology, botany and zoology.**

Did the permission you obtained from a local authority to perform the study include an agreement on access to outputs and benefit sharing? This may include procedures to enable fair distribution of the benefits and resources arising from the research performed. Please include any details of Prior Informed Consent and Benefit Sharing Agreements obtained. These may be required by field-specific regulations, for example the Convention on Biological Diversity (CBD) and the associated Nagoya Protocol.

N/A

If the material used in your study was imported, please A) provide the year it was imported and B) indicate whether permits were obtained to import/export the materials used, C) provide details of any permits obtained. If this information is not available, please indicate this.

N/A

If you used archival specimens, please state how the material used in your study was acquired by the institute it is held in and provide details of any permits obtained for the original excavations/ sample collection. If this information is not available, please indicate this.

N/A

How was the potential cultural significance of the materials collected in your study to local communities considered in your research design? Were Indigenous peoples and/or local researchers and institutions involved with archaeological excavations / collection of specimens? If so, please provide a description of their involvement.

N/A

If your manuscript includes photographs of human remains, please indicate whether authors obtained permission from descendants or affiliated cultural communities to do so.

No
