# Supplementary material for: Developing an intervention to improve early infant HIV diagnosis service uptake among postpartum women in Malawi’s primary healthcare using a co-designing approach with stakeholders
Source: PLOS Glob Public Health. 2025 Apr 22;5(4):e0004426. doi: 10.1371/journal.pgph.0004426 (PMC12013899; doi:10.1371/journal.pgph.0004426)
Supplement: S2 Text — (PDF) [file pgph.0004426.s002.pdf]

Aim of the study

- Explored performance and process of care provided to infants exposed to HIV from birth to 6 weeks of age in two primary health care facilities in Blantyre, Malawi, to inform strategies to improve uptake of EID services

Data collection

Retrospective review of EID data

- Data extraction
  - Routinely collected HEIs data
    - Jan- Dec 2018

Process mapping

- Mapping HEIs journey of HIV care
  - Postnatal ward to exiting facility
  - Immunisation /postnatal clinic to exiting facility

Semi- structured interviews

- Group semi structured interviews with key health workers, following findings from process maps
  - Four in each group

Characteristics of women with live infants exposed to HIV, Jan- December 2018

	Total<br>Both Facilities<br>N(%)<br>N=163	Urban<br>N(%)<br>N=123	Rural<br>N(%)<br>N=40
Number of women with live HEIs	163(100)	123(75)	40(25)
Age in years for women with HEIs in categories<br>17-24<br>25-34<br>>35	38(28)<br>77(47)<br>48(29)	28(23)<br>58(47)<br>37(30)	10(25)<br>19(47)<br>11(28)
Median age(age range))	28(17- 48)	27(17- 48)	30(17- 43)
Women with infants documented ART facility	123(75)	86(69)	37(93)
Women receiving ART at facility of delivery	93(76)	63(73)	30(81)

Outcomes of infants exposed to HIV at 6 weeks from July-December at Urban and January –November 2018 at Rural facilities

Variables	Both	Urban	Rural
All infants exposed to HIV born at facility	163(100)	123(100)	40(100)
Infants exposed to HIV ever enrolled in HIV care	98(60)	66(53)	32(80)
Infants exposed to HIV enrolled in HIV care before post-natal discharge	39(24)	16(13)	23 (57)
Infants without documentation of enrolment	65(40)	57(46)	8(22)
HIV-tested(DNA-PCR, POC or DBS)	85(52)	57(46)	8(22)
HIV testing time in weeks IQR	6(5-20)	6(5-16)	6(5-20)
HIV- Positive	2(3)	0(0)	2(4)
HIV-Negative	77(90)	54(94)	23(79)
Infants exposed to HIV without results	6(8)	3(5)	3(11)

Infants exposed to HIV process of HIV care journey in selected health facilities (February – March 2020

- Of the eight mother-child pairs
  - Three were followed at birth and five at 6 weeks
  - Three in rural and Five in urban
  - Median age 31(16-34) years
  - Median ART duration 9 (6-36) months
  - Median number of Children 2(1-4)

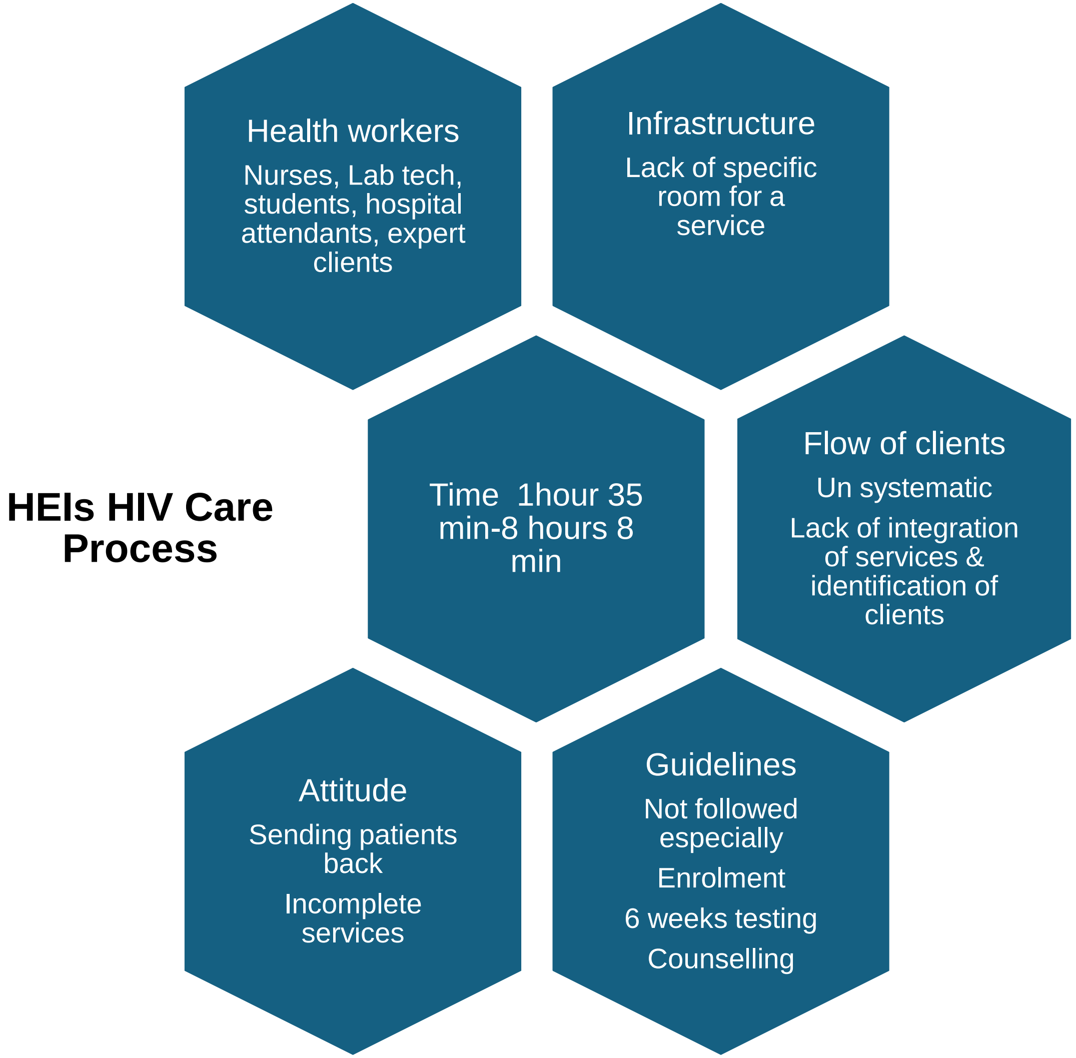

Healthcare workers' perspectives on the process of HEIs HIV care in selected health Facilities March 2020

- Of the 16 health workers
  - 8 from rural
  - 13 female, 3 male
  - Comprised of HSA, nurses, data clerks, medical assistants, medical officer
- Time contributing factors
  - Poor flow of care
  - Health workers arrangements
  - Late reporting

One of it could be late reporting for duties by hospital staff

- HIV care service provision
  - Inadequate understanding of responsibilities
  - In availability of qualified workers on duty
  - Attitude
  - Use of lay cadres

.... Let me give a picture of our laboratory staff, we have technician, lab Micropist who is trained on all lab work but am not a counsellor. The time they were training counsellors they also trained some HSA's but they don't work in the lab..... We have one counsellor and one technician but at the moment they are in Mchinji but we still have to test the child.

Incomplete service contributing factors

- Lack of screening
- Poor coordination
- Assumption that care will be provided elsewhere
- Unclear instructions to clients

Most of the times these women leave the lab by 2 or 3 pm and by this time family planning has already closed that is why most of them go back without accessing family planning.
