# Supplementary material for: Developing an intervention to improve early infant HIV diagnosis service uptake among postpartum women in Malawi’s primary healthcare using a co-designing approach with stakeholders
Source: PLOS Glob Public Health. 2025 Apr 22;5(4):e0004426. doi: 10.1371/journal.pgph.0004426 (PMC12013899; doi:10.1371/journal.pgph.0004426)
Supplement: S3 Text — (PDF) [file pgph.0004426.s003.pdf]

EEHS Intervention

- Enhanced Health Systems for Postpartum Women (EEHs)

  - A set of four initiatives to improve client identification, provision of individualised comprehensive care for both mother and child to primarily improve uptake of early infant diagnosis of HIV

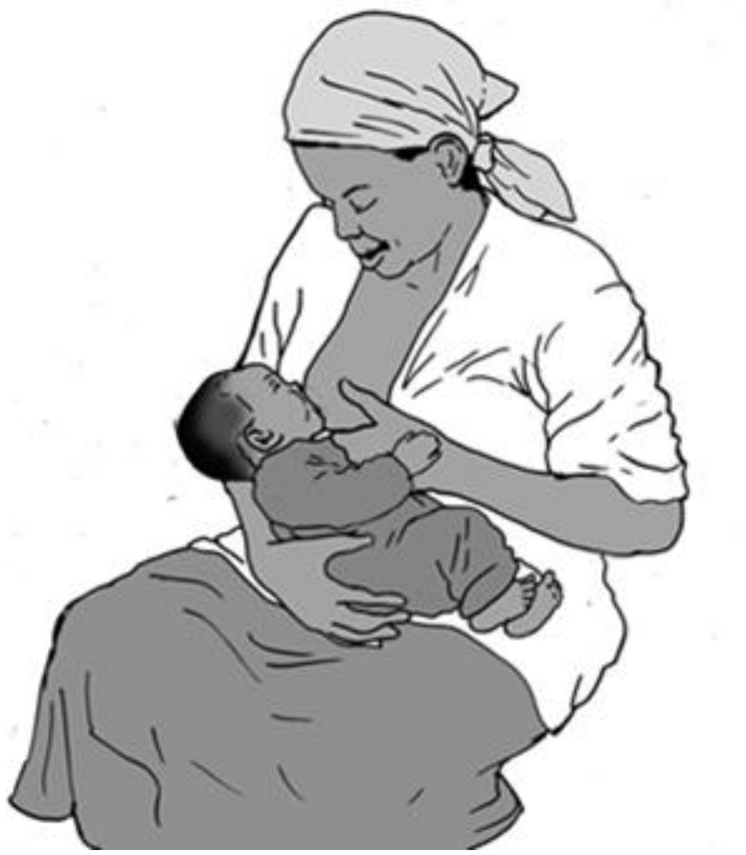

Key co-designed initiatives to address health systems challenges

Active client identification and Tracking

- Failure to identify clients within the health system, poor flow of care, lack of individualised comprehensive care

Booking system for HIV testing using POC machine

- Increased waiting time, returning clients home without HIV testing and other HIV care

Daily data validation

- Poor data quality(incomplete documentation inconsistences, illegibility) Missing Registers , inadequate handovers on data

Strengthened leadership

- Poor coordination, lack of compliance to guidelines, ineffective quality improvement teams (dependent on availability of allowances), inadequate enrolment of infant in HIV program, Poor documentation

Aim	What	Who	Where/When	Actors
Active client identification and tracking	Indicate unique labels<br>Actively screen for the identifiers or HIV status<br>Information giving<br>Offer self test if mother HIV status unknown	All nurse-midwives, clinicians and support staff	The antenatal and postnatal clinic, labour ward and under-five clinic	The antenatal and postnatal clinic, labour ward and under-five clinic
Improve motivation of health workers, teamwork and compliance to PMTCT guidelines through strengthened leadership	Placement of under24-months cared and HCC register at labour ward<br>Task allocation<br>Improved hand overs<br>Equipped PMTCT focal person<br>DHMT support	All nurses and support staff, PMTCT focal person, Health centre management and DHMT	Labour ward	Meeting and training all staff<br>Consult nurse in charge to guide selection of PMTCT focal
Booking system for MIP	Make available a book for booking POC HIV testing. Assign date for HIV testing in line with other services not exceeding the capacity of the POC on a particular date	All nurses and support staff	At one week postnatal check-up	Meeting and training with all staff
Improve data management	Improved handovers<br>Data validation<br>Back up tablets during data collection	Health workers, ART data clerks , PMTCT focal [illegible]	ART clinic, labour ward and HIV testing room	Meeting and training with all staff<br>Training research assistants

Minimum package of initiatives

Initiative	SOP Number	SOP Sections
**Understand active identification and booking**	SOP 1	1. At admission
		3.At Handovers
		4.At Weighing area & consultation
		2.At birth
		3.At one week
		4.At 6 weeks
**Booking system for MIP**	SOP 2	1.Booking at birth
		2.Verification at one week
		3.Verification and testing at 6 weeks

Initiative	SOP Number	SOP Section
**Strengthened leadership**	SOP 3	1.Develop task allocation
		2.Appoint PMTC focal person
		3.Improved Handovers with verification of documents
**Data validation**	SOP4	1.Review of documents during hand over and end of shift
		2.Back up tablets for the study

Active identification and tracking

- **There are six sections for health care workers to actively identify and track patients during post-natal period from birth to 6 weeks**
  - Admission
  - Handover
  - Weighing area and any encounter
  - Post-natal at birth
  - Post-natal at one week
  - Post-natal at six weeks
- **Overall, to actively identify and track women with HEI during the post-natal period, healthcare workers should:**
  - Indicate letter Q on the ANC page of the health passport book of a PMTCT mother and the infants passport book
  - Give mother-infant pair (MIP) information on where to first report at subsequent visits before post-natal discharge
  - **Health care workers should do the following to ensure the tracking of an HEI**
  - Check for letter Q on the ANC page, or HIV status, on the middle page of the mother's health passport book at any encounter before providing care

Active identification and tracking

- Please do not ask the mother about her status in a clinic queue;
  - Check for letter Q OR HIV status to track an HEI to enhance privacy
- Once identified, health care worker (HCW) should give information and direct MIP where to report first for care
- Link women with HEI with unknown HIV status to the HDA to avoid waiting in a queue for HIV tests
- Refer to the active identification and tracking flow chart next slide
  - Discuss the chart to suit context of your facility what can work and what can not work?

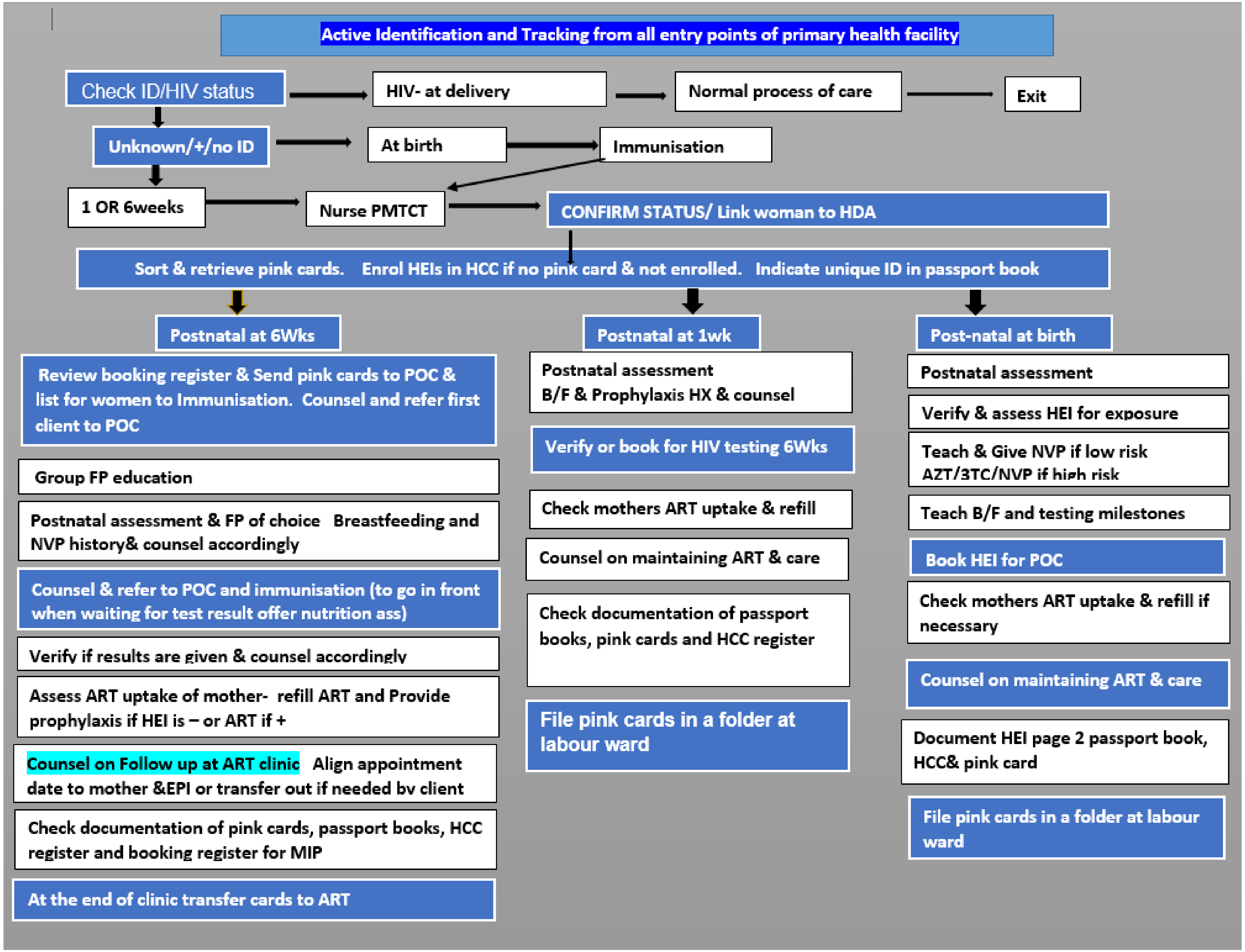
Active Identification and Tracking from all entry points of primary health facility
Check ID/HIV status
HIV- at delivery
Normal process of care
Exit
Unknown/+/no ID
At birth
Immunisation
1 OR 6weeks
Nurse PMTCT
CONFIRM STATUS/ Link woman to HDA
Sort & retrieve pink cards. Enrol HEIs in HCC if no pink card & not enrolled. Indicate unique ID in passport book
Postnatal at 6Wks
Review booking register & Send pink cards to POC & list for women to Immunisation. Counsel and refer first client to POC
Group FP education
Postnatal assessment & FP of choice Breastfeeding and NVP history& counsel accordingly
Counsel & refer to POC and immunisation (to go in front when waiting for test result offer nutrition ass)
Verify if results are given & counsel accordingly
Assess ART uptake of mother- refill ART and Provide prophylaxis if HEI is – or ART if +
Counsel on Follow up at ART clinic Align appointment date to mother &EPI or transfer out if needed by client
Check documentation of pink cards, passport books, HCC register and booking register for MIP
At the end of clinic transfer cards to ART
Postnatal at 1wk
Postnatal assessment
B/F & Prophylaxis HX & counsel
Verify or book for HIV testing 6Wks
Check mothers ART uptake & refill
Counsel on maintaining ART & care
Check documentation of passport books, pink cards and HCC register
File pink cards in a folder at labour ward
Post-natal at birth
Postnatal assessment
Verify & assess HEI for exposure
Teach & Give NVP if low risk
AZT/3TC/NVP if high risk
Teach B/F and testing milestones
Book HEI for POC
Check mothers ART uptake & refill if necessary
Counsel on maintaining ART & care
Document HEI page 2 passport book, HCC& pink card
File pink cards in a folder at labour ward

Section 1:Active identification and tracking on admission

- **Admit the woman in labour**
- Establish the HIV status of the woman, either -ve or +ve.
- Offer new HIV test for all women with:
  - Unknown HIV test results
  - Negative test results any time in the past, even last trimester
  - Arrange with HDA to screen women in labour ward twice a day to test women not in active labour & after birth
  - **Documentation of HIV status in Maternity register -**
- **For HIV-positive women indicate a unique label , letter Q**
  - On the ANC page of mother's health passport book,
- Assess HIV-positive women at increased risk of HIV transmission to new-born (high-risk exposed infant)
  - Follow questions on slide 11, if no VL results, assess adherence by asking pill count
- Do clinical examination and refer accordingly
- Check if the woman has brought her ARVs and prophylaxis 2P or NVP syrup

Section 2: Active identification and tracking during handovers

- Nurses should **provide and receive morning handover that includes information about all PMTCT women** in labour and post-natal ward at the primary health facility as follows:

  - Total number of PMTCT women,
  - Total number of women whose HIV status is known from the labour ward,
  - ART status of PMTCT women
  - HEI enrollment in HCC and administration of prophylaxis

Section 3: Active identification and tracking at weighing area

- Any health worker should know that they will meet women with HEIs of different ages at the weighing area as follow:
  - **Child at birth, one week and six weeks**

- Patient attendant, Health surveillance assistant (HSA), or any health worker should :
  - **Check the mothers' health passport book for a unique ID, letter Q or HIV status when weighing the infant**
  - **Separate passport books with letter Q, positive HIV status or unknown HIV status from the rest**
  - **HSA should offer immunisation to infants getting discharged at birth before referring them to post-natal nurse**
  - **Handover the books to a nurse offering post-natal care**
  - **Invite women with HEIs to a room with a nurse to provide all services required by the MIP**
  - **Link women to HDA if the status is unknown**
- Offer care to women with current HIV negative results (HIV test at the labour ward) according to the post-natal guidelines

Section 4: Active identification and tracking discharging women at birth

- A nurse or any health care worker should:
  - **Check and confirm the HIV status of the woman or letter Q in the health passport**
    - If unknown link the woman to the HDA for HIV testing
    - **Indicate unique ID, letter Q on the ANC page of the health passport book of the mother if not available**
  - Sort the passport books according to the age of HEI and the service to provide:
    - At birth, One week, At six weeks

- **Start providing services to women at six weeks, following the section for six weeks**,
  - Refer to section 6 active identification tracking at six weeks

Postnatal at birth

- Verify if the passport book has been created and If not available, create
- Verify if a weight has been checked
- Assess if the baby is feeding well
- Verify if the baby was assessed for a high-risk HIV-exposed infant status at birth

- Assess if the HEI is a high risk or not ,the baby is a high risk if there is a yes on the below questions
  - **Did the woman start ART when she was pregnant during her third trimester**
  - **The woman never started ART during pregnancy**
  - **Those women starting ART in maternity**
  - **Those women with interrupted ART uptake during pregnancy**
  - **Women with high viral load**
  - **Unknown viral load? What is ART adherence? Pill count?**
- Check if the mother knows the administration of prophylaxis and teach accordingly, guided below:
  - **Give NVP according to body weight for low-risk exposed infant**
  - **Give AZT/3TC/NVP 2 tablets for high-risk exposed infant**
  - **Document in HEI passport book page 2(if available) or any page if ordinary passport book, pink card and HCC register**

Postnatal at birth

- Indicate the HCC number next to the initiation and follow-up words on the infant passport book
- Indicate actual prophylaxis given under NVP section in the health passport book
- Enter HEI in the HCC register and transfer out if the mother receives ART elsewhere
- **NB: Document the above if using an ordinary book where there space in the passportbook**

- Offer ARVs to the mother if she has run out of drugs
  - If she collects her medications elsewhere, give her an emergency supply
- **Document HEIs in the HIV-testing booking register regardless of where the mother collects her ARVs**
- Document in HEI passport book page 2 or any page if using ordinary book, pink card and HCC register
- **Indicate the HCC number next to the initiation and follow-up words in health passport book**
- **Indicate actual prophylaxis given under NVP in health passport book**
- Enter HEI in the HCC register and transfer out if the mother receives ART elsewhere
- Offer ARVs to the mother if she has run out of drugs
- If she collects her medications elsewhere, give her an emergency supply
- **Document HEIs in the HIV-testing booking register regardless of where the mother collects ARVs**

Postnatal at birth

- Agree with the HSA on duty on the next appointment date to give PMTCT women
- Check and align the next appointment date with EPI date, family planning date and date in the booking register by verifying EPI appointment date and booking register capacity
  - If booking capacity date is full, liaise with HSA to change the date for EPI
  - Aligns next appointment date with the mother ART appointment date
  - Explains the need for testing the child at three different milestones. Six weeks, 12 months and 24 months
- File pink cards in a folder according to the birth date or cohort

Section 5:Active identification and tracking at one week

- Nurse or health care workers should:
  - Check and confirm HIV status of woman in health passport
    - Look for a unique ID letter Q or HIV status on middle page of health passport book
  - If HIV status is unknown link the woman to the HDA for HIV testing(explore how to do this at the facility)
  - Indicate the agreed unique ID letter Q, on the ANC page of the health passport book of the mother
  - Conduct post-natal assessment for the mother and infant according to the existing guidelines

Section 5:Active identification and tracking at one week

- Verify the following information using the HEI passport book on page 2 OR any page of the ordinary health passport bookif the information was documented on previous visits
  - Assessed for a high-risk status,
  - Booked for HIV testing
  - Uptake of Prophylaxis
  - HEI is enrolled in HIV care
- If information is not available, refer to the Exposed pink card and HCC register, and the booking register
- If any of the answers to the above questions is NO, then follow the procedure for consultation at birth
- Remind women on the uptake of prophylaxis
- Remind mother of the appointment dates at six weeks in line with the date in the booking register
- Provide information to the mother-infant pairs on where to report for subsequent visits after birth (nurses' office at the post-natal clinic)
- Teach the importance of making sure the mother returns to the facility at 6 weeks

Section 6:Active identification and tracking at six weeks

- Check and confirm the HIV status of the woman in the health passport
  - **Look for a unique ID letter Q on the ANC page or HIV status on the middle page of the health passport book**
  - **If unknown link woman to the HDA for HIV testing**
  - **Indicate the agreed unique Id letter Q on the middle page of the health passport book of the mother**

- Provide education to all women, including family planning information

- The nurse compiles pink cards for available HIV-exposed infants, and the booking register

- The hospital attendant compiles a list of women to give to the immunisation clinic for women not to queue

- Advise women to go in front and meet up with an HSA for immunisation at the clinic and show the page with the unique ID letter Q

Section 6:Active identification and tracking at six weeks

- The nurse should provide pre-test information and seek consent for testing

Group Reflection

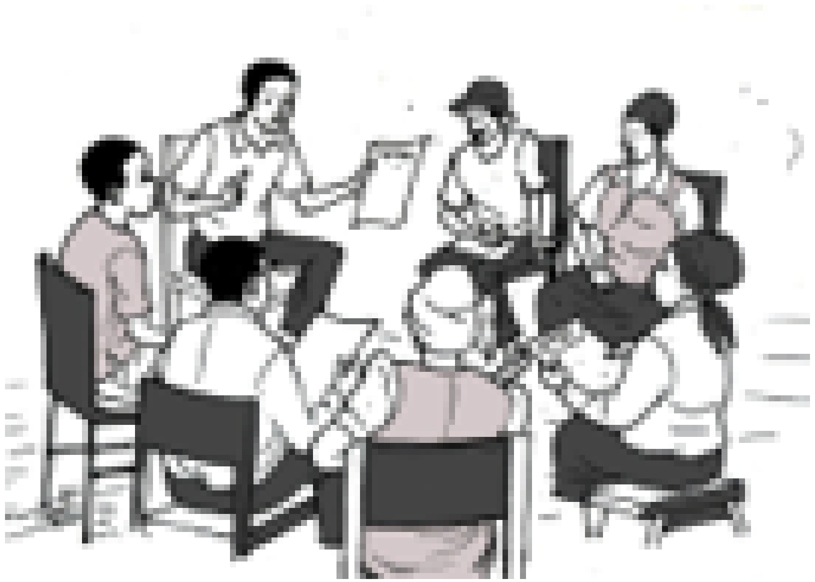

Section 6:Active identification and tracking at six weeks

- Pre-test information
  - The benefit of the test and why the infant should be tested
    - The importance of early HIV diagnosis for mothers and infants
    - The risk of transmitting HIV to the infant
    - The importance of ART for the mother's health and in preventing transmission to the baby
    - Counselling on infant feeding practices to reduce the risk of HIV transmission
    - Partner and family testing

Section 6:Active identification and tracking at six weeks

- Hospital attendants take pink cards and booking register to the HIV testing room
- Manage time and refer one woman for POC when providing postnatal care to the rest
- Conduct postnatal assessment for the mother and infant according to the existing guidelines
- Provide family planning according to the choice of the client
- Obtain a history of breastfeeding and prophylaxis
- Verify infant enrolment in HCC
- Inform mother about HIV testing and testing milestone
- Assess if a child requires a test

Section 6:Active identification and tracking at six weeks

- Refer the woman to the testing room and give directions
  - If the woman is unable to understand directions, ask hospital attendant to escort
- Inform women that they will come back to nursing consultation
- In the POC testing room, provider should check if the mother has information about HIV test
  - If mother does not know about HIV testing, counsel before testing infant
- Inform mother of procedure for testing and time it may take for results to come out
- After taking a sample from an HIV-exposed infant for HIV testing using POC
  - provider must check if MIP has already received family planning and immunisation

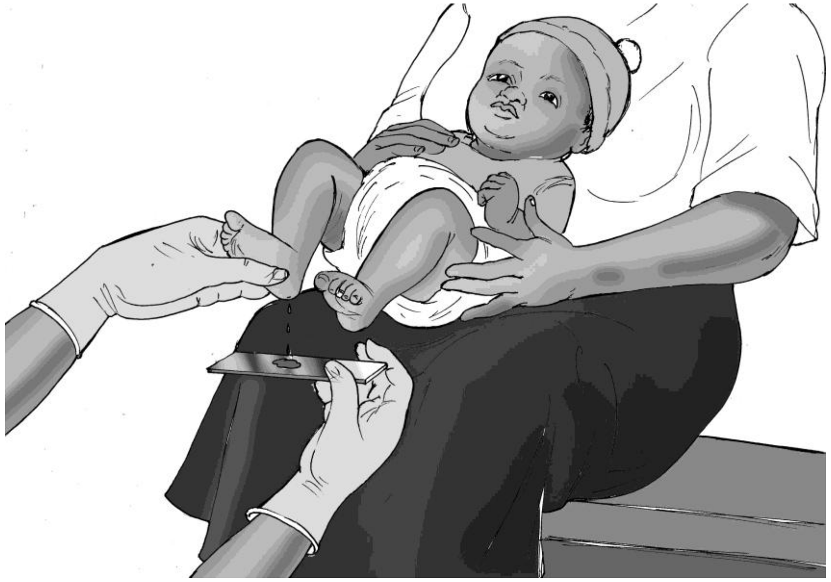

Section 6:Active identification and tracking at six weeks

- If collecting samples using DBS
  - MIP must not return to the testing room after referring them for immunisation and family planning

- If the mother-infant pair has not received immunisation for the baby and her family planning method
  - Inform mother to meet HSA for immunisation without being on the immunisation queue
  - Then the nurse for family planning without being on the family planning queue
- The HSA should provide immunisation and check the weight and height of the HEI
- Ask the mother to come back to the POC testing room to receive results immediately after accessing immunisation and family planning.
- Provide post-test counselling and results after 1 hour if using POC /not more than 14 days if using DBS

Section 6:Active identification and tracking at six weeks

- After providing the results, refer the mother and HEI back to the postnatal nurse
- Verify if the mother has received results for the infant
- Nurse should explain in simple terms the medication given to the infant depending on the HIV test result outcome
- Verify if the child has been weighed and checked for height
- Explain to mother nutrition status
- If HEI is negative, provide Cotrimoxazole therapy for the infant according to body weight
- If HEI is positive, initiate baby on ART(take the baby to the ART clinic), following the protocal
- Assess the ART status of the mother
- Provide ARVs for the mother
- Mother-infant paring (align mother and infant dates)
- Document and transfer out if the mother collects ART at another health facility

Module 4: Standard operating procedure 2
Booking system for MIP
Time: 60 minutes

Booking System for MIP

- Presence of a book at the postnatal clinic
  - Nurses or support staff will document MIP to be tested on a particular day,
  - Up to the number the facility POC machine can test per day
  - Three MIP to accommodate other walk in MIP
- Once that number is reached
  - follow-up clients will be booked for the next day
  - Number three is considered to accommodate walk in MIP from other facilities not booked in at birth
- There are three main points for health care workers to use the booking register, but can also use at any encounter with MIP
  - Postnatal at birth, postnatal at one week and postnatal at six weeks.

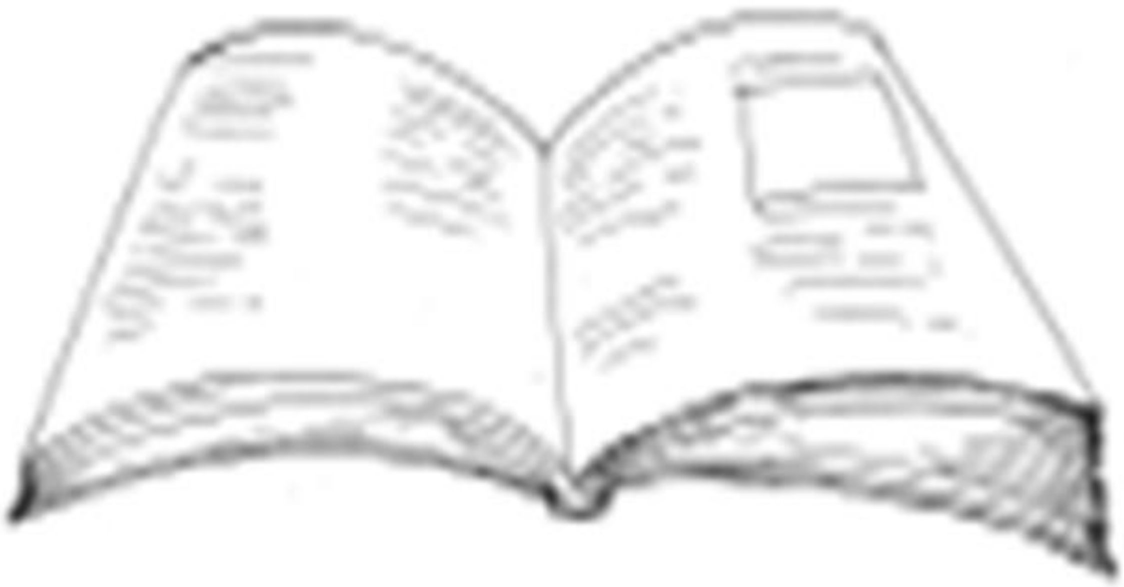

MIP BOOKING REGISTER

Serial #	Name	HCC number	Booking date	EPI appointment date	Mothers ART appointment date	DBS appointment date	Family Planning date	POC testing appointment date	Mothers ART site	Contact information	Comments
1											
2											
3											
POC testing capacity											

Section 1:Booking at birth

- Have a booking register at the beginning of the clinic at discharge after delivery

- Document HEI in HIV MIP booking register regardless of where mother collects ARVs

- Fill in the following variables:
    - Serial number, not to exceed 3 because testing capacity is 5 and 2 is to accommodate walk ins
    - Name of the HEI, HIV care clinic number,
    - EPI appointment date, Mothers ART appointment date, Family planning appointment date
    - DBS appointment date, for facilities with DBS, they can schedule more than three due to capacity of DBS but have to accommodate other services
    - POC HIV testing date
    - Where the mother access ART
    - Contact information for the mother
    - Comments sections

Section 1:Booking at birth

- If the booking capacity date is full(three people where the POC capacity is five in a day)
  - Liaise with HSA to check and align next appointment date around six weeks with immunisation and the date in the booking register
  - Verify immunisation appointment date and booking register capacity, and the mothers' appointment date
  - Change date for immunisation and change mothers ART date

- Full booking date means:
  - Having three HEIs per day to offer HIV testing using a POC machine
    - Testing more than five HEIs on a single day will exceed the POC testing capacity and time when clinics operate each day
    - Two clients are reserved for walk in clients

- Align all appointment dates, immunization, HIV testing, ART date, EPI date

Section 2: Booking at one week

- When giving the mother an appointment date,
  - Verify if six weeks appointment date was given

- If it was given, check if
  - The HEI was entered in the booking register and if it corresponds to the given appointment date at six weeks
  - If it does not correspond to the given date at six weeks correct it accordingly following the steps at birth

- If the next appointment date was not given:
  - Follow the process at birth to give the appointment

Section 3: Booking at six weeks

- The nurse should compile pink cards for available HIV-exposed infants and verify with the booking register

- Support staff or the nurse should take the files to the POC care testing room

- Mark all HEI in the booking register that has received HIV tests and gives the name that did not come to Expert clients for tracing

- Transfer the pink cards to the ART clinic after HIV testing and tick all MIP that came

Strengthened Leadership

- **Appointment of a midwife to be a focal person who will**
  - **Work with the nurse in charge to lead the team of nurses in the labour and postnatal ward**
  - **To comply with PMTCT guidelines and EHHs intervention**
  - **The PMTCT focal person will not replace EID focal person**
- The focal person will lead supportive facility mentorship based on gaps from handovers and staff needs The focal person will facilitate that
  - Nurse managers should develop a task allocation rotter accommodating PMTCT services
  - Initiate meetings to enhance care provision(sharing progress and updates on EIDservices)
- There are three action points to strengthen leadership and team working
  - 1. Develop task allocation
  - 2. Appoint PMTCT focal person
  - 3. Improved handovers
  - 4. CPD

Section 1: Develop task allocation for shifts

- Complete the task allocation template every Thursday of the week
- Allocate tasks evenly amongst team members, making use of team strengths or areas where development is desired
- Ensure agreement from team members to take on specified allocated tasks by Friday of the week
- Provide frequent opportunities for feedback to team members from individuals
- Re-allocate roles and responsibilities only after agreement with all of the relevant team members

Group Work : Task allocation templates

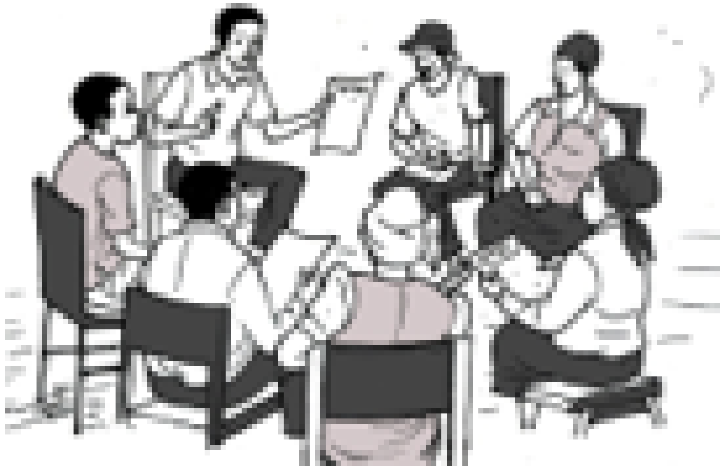

Section 2: Appoint an equipped focal person

- Appoint an equipped PMTCT focal person
- Let people that are willing to be the focal person express interest
- Let the nurse in charge and the PMTCT focal person consider the list of interested people
- Qualities of the PMTCT focal person include:
  - Must be a nurse or midwife trained in PMTCT
  - Passion with PMTCT
  - Willing to work hand in hand with the nurse in charge and lead a team of nurses in the PMTCT programme
  - The focal person must work towards bringing all nurses and other health workers working in PMTCT work together
  - PMTCT focal person is not replacing EID focal person

Section 2: Appoint an equipped focal person

Group work: Roles of PMTCT focal person

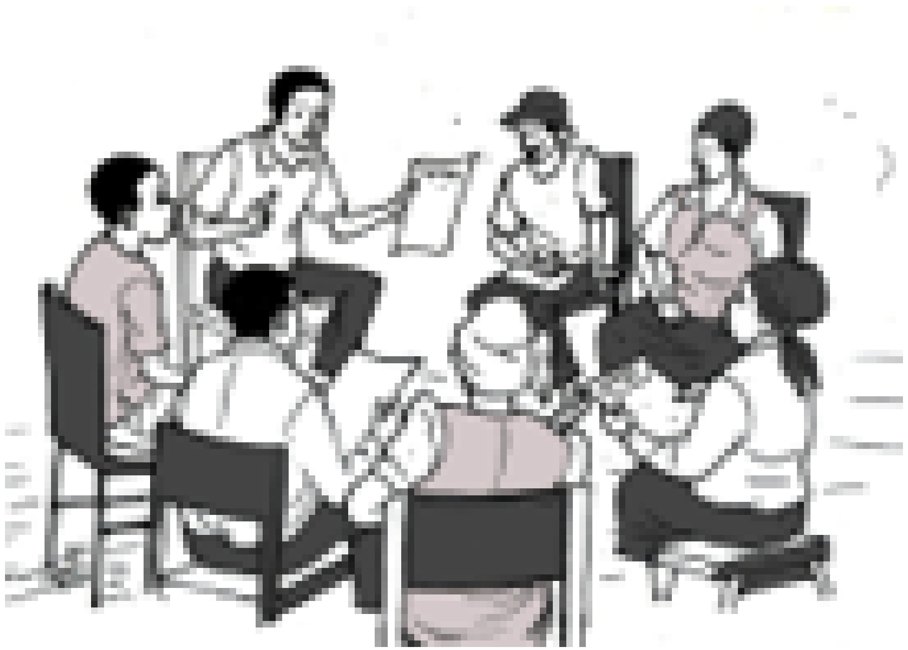

Section 2: Appoint an equipped focal person

Roles of PMTCT vs EID

PMTCT focal must ensure PMTCT measures at ANC and Labour ward are complied and must be a qualified nurse

1. HIV exposed infant enrollment according to protocol
2. Testing according to protocol
3. Verifying documentation of data sources including HIV status, booking register and cards at the labour ward
4. Enrolment of HEI at birth and documentation
5. Enhancing improved handovers and

EID focal person

Can be of any cadre, and the facility can decide to have PMTCT only or both

1. Ensure HIV testing according to protocol
2. Report writing
3. Verifying documentation of data sources including enrollment if missed at birth

Section 3: Conduct handovers with verification of documents

- **When writing handovers**
- Pull out all case notes to be documented
  - Collect all files for PMTCT women that have given birth
  - Document patients as you usually do in the handover book
  - The section for statistics includes the total number of PMTCT women who have given birth for each particular shift
    - Indicate the total number of HEIs that you have enrolled in HCC and those to be enrolled

Section 3: Conduct handovers with verification of documents

- **During Handover**
- Read out at the morning hand over
- At the ward
  - Give actual passport books or case files for women with HEI to the nurse allocated on postnatal PMTCT(if not available, give it to the available nurse in the postnatal ward) and mention if women with HEIs are not enrolled in HCC
  - If enrolled in HCC, verify documentation in the HCC register and the card with the nurse receiving the handover
  - Follow the exact instructions above when writing and giving handover at the end of a shift

Section 3: Conduct Continuous professional development meetings

- Duration of CPD meetings
    - Facility should agree when to conduct the CPD meetings, time and rotter for presentations
    - To accommodate facilitating the EEHs intervention to new members of staff
    - To accommodate facilitation of the EEHs intervention to those having challenges

Data Validation

- Ability of nurses to give each other handovers
    - reviewing the documents of HEIs born on a shift
    - deliberate checking of records at the end of each shift or clinic to ensure data quality
- As part of the research
    - Backup data entry on tablets by research assistants and the researcher
    - To compare to the routinely collected data captured through pictures with hidden identifiers

Data Validation

- **Resources required**
- To have a fruitful handover, the following should be available in maternity or placed in labour ward.
  - Pink cards
  - Maternity register, HCC register (rural facility to agree depending on context)
  - Report book
- People receiving handover should verify the documents while the nurse on duty reports from the report book

Section 1: Review of documents

- In line with received handovers, check the following:
  - Women with HEI are enrolled in the HCC register
  - Verify if all files are correctly documented, and all spaces are filled
  - Verify if all booked HEIs for that particular day were tested for HIV
  - File pink cards and HCC registers at the labour ward,
  - Place a folder with both blank and filled pink cards at the maternity
  - File pink cards according to the birth cohort
  - Transfer the pink cards to the ART at six weeks after offering HIV testing and counselling
- PMTCT focal and in charge should orient New nurses and students on the nature of handover handover
  - Enter enrolment and tested HEI figures on a templet excel sheet on the phone (What's up group)

Facility		**Hypothetical numbers, replace with actual data	
	date	enrloment	6wks testing
	1	3	2
	2	2	4
	3	1	1
	4	4	3
	5	2	1
	6	6	3
	7	3	1
	8	2	0
	9	4	2

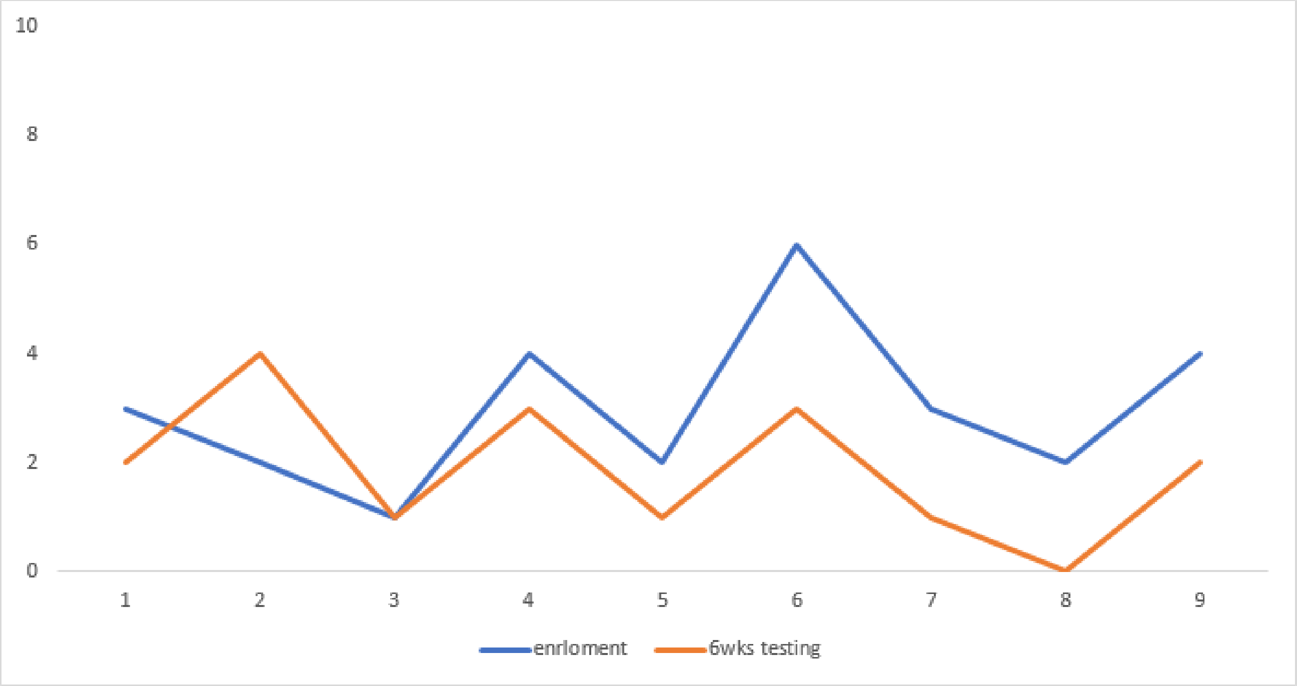

Section 2: Back up data

- Back up tablets for backup data entry for the study
- Orient field workers on the objective requiring data collection
- Orient field workers on the data variables to be collected refer to the CRF
- Pilot the CRF
- Orient the field workers, to take pictures without identifiers in the registers
- List of variables not to capture in the pictures
- List of variables to ensure they are captured
- Orient field workers to enter the data on the pictures taken on a spreadsheet on the computer
