# Supplementary material for: Developing an intervention to improve early infant HIV diagnosis service uptake among postpartum women in Malawi’s primary healthcare using a co-designing approach with stakeholders
Source: PLOS Glob Public Health. 2025 Apr 22;5(4):e0004426. doi: 10.1371/journal.pgph.0004426 (PMC12013899; doi:10.1371/journal.pgph.0004426)
Supplement: S4 Text — (DOCX) [file pgph.0004426.s004.docx]

| **Continuation of Baby** | | | | | | | | |
| --- | --- | --- | --- | --- | --- | --- | --- | --- |
| **Continuation of PMTCT management** | | | | | | | | |
|  | VL result | | Adherent | | HEI Risk status | | 2P |  |
|  | Yes | No | Yes | No | High | Low | 70 |  |
|  | 67 | | 68 | | 69 | |  |  |
|  | H | LDL | Y | N | H | L |  |  |
|  | H | LDL | Y | N | H | L |  |  |
|  | H | LDL | Y | N | H | L |  |  |
|  | H | LDL | Y | N | H | L |  |  |
|  | H | LDL | Y | N | H | L |  |  |
| Page Summary |  |  |  |  |  |  |  |  |

**Additional Variables in maternity register**

Field Number

Note: This part is completed to support healthcare worker

Numbers 67, 68, and 69 stand for variables, viral load result, adherence and prophylaxis respectively. The numbers were used to pilot how the rest of variables are entered on DHIS 2 (Reporting platform) for reporting. However, these variables were not reported on DHIS 2 because this was a pilot.

The letters have different meanings, as explained below. HCW circles one letter in each column depending on their assessment or action.

Viral load result column, H mean high viral load result, and LDL mean low level detected

Adherent column Y mean yes, and N mean No to the mother of HIV exposed infant being adherent to ARVs

The high-risk status column H means the infant is at high risk, and L infant is at low risk

2P is a high-risk prophylaxis given
