# Supplementary material for: Developing an intervention to improve early infant HIV diagnosis service uptake among postpartum women in Malawi’s primary healthcare using a co-designing approach with stakeholders
Source: PLOS Glob Public Health. 2025 Apr 22;5(4):e0004426. doi: 10.1371/journal.pgph.0004426 (PMC12013899; doi:10.1371/journal.pgph.0004426)
Supplement: S5 Text — (PDF) [file pgph.0004426.s005.pdf]

BOOKING SYSTEM REGISTER

Health facility: ……………………………………………………

Date register started: ………………………………………………...

Date register closed ……………………………………………….

Version 1

General instructions

1. This register will be used to book HIV-infected women and their infants for a six-week check-up and all required services.
2. The appointment dates for different services should be the same to ensure that the mother and infant receive all services.
3. Healthcare workers, i.e. nurses and HSA, must discuss the appointment dates before giving an appointment.
4. Consider the availability of resources when giving the appointment date.
5. If one service is not available on a particular date, change the appointment to the date that will carter all services
6. There are three boxes, each with serial numbers 1 to 3, to use to book a mother and an infant
7. If one box is completed, start the next box on the same page before turning to the next page
8. Mother and infant in one box can be booked on different dates depending on the situation
9. Similarly, mother and child in one box can have different appointments dates depending on the situation
10. There are two significant columns for the HIV-Exposed infant and Mother. Write information for each in each respective column

Selected columns instructions

Column	Instruction
Serial #	Running numbers
HCC #	Extract an HIV care clinic number from the HCC register
DBS data	For facilities with the point of care, the machine does not indicate a date on DBS at the booking<br>Only fill in the DBS data on the date you took DBS if POC was not available and write a comment
ART site	Include an ART number on the ART site if it is available

HIV-EXPOSED INFANT								MOTHER							
Serial #	Booking date	Name	Date of birth	HCC #	EPI date	DBS date	POC date	Name	ART site	ART date	Family planning date	Postnatal checkup date	Contact information	Physical address	Comments
1															
2															
3															

HIV-EXPOSED INFANT								MOTHER							
Serial #	Booking date	Name	Date of birth	HCC #	EPI date	DBS date	POC date	Name	ART site	ART date	Family planning date	Postnatal checkup date	Contact information	Physical address	Comments
1															
2															
3															

HIV-EXPOSED INFANT								MOTHER							
Serial #	Booking date	Name	Date of birth	HCC #	EPI date	DBS date	POC date	Name	ART site	ART date	Family planning date	Postnatal checkup date	Contact information	Physical address	Comments
1															
2															
3															

HIV-EXPOSED INFANT								MOTHER							
Serial #	Booking date	Name	Date of birth	HCC #	EPI date	DBS date	POC date	Name	ART site	ART date	Family planning date	Postnatal checkup date	Contact information	Physical address	Comments
1															
2															
3															

HIV-EXPOSED INFANT								MOTHER							
Serial #	Booking date	Name	Date of birth	HCC #	EPI date	DBS date	POC date	Name	ART site	ART date	Family planning date	Postnatal checkup date	Contact information	Physical address	Comments
1															
2															
3															

HIV-EXPOSED INFANT								MOTHER							
Serial #	Booking date	Name	Date of birth	HCC #	EPI date	DBS date	POC date	Name	ART site	ART date	Family planning date	Postnatal checkup date	Contact information	Physical address	Comments
1															
2															
3															
