# Supplementary material for: Developing an intervention to improve early infant HIV diagnosis service uptake among postpartum women in Malawi’s primary healthcare using a co-designing approach with stakeholders
Source: PLOS Glob Public Health. 2025 Apr 22;5(4):e0004426. doi: 10.1371/journal.pgph.0004426 (PMC12013899; doi:10.1371/journal.pgph.0004426)
Supplement: S1 Data — (ZIP) [file pgph.0004426.s008.zip › S1_Data/Designing additional page clean 150823.docx]

Designing an additional page

## Group1

I will share what we have discussed. We have discussed that all registers in all entry points should have this high-risk column added. i.e. ANC, FP, HCC, maternity to cater for all patients where we have identified the person

We should have a key. Then, each column can have different letters (A, B, C, D, E, OR 12345), which will be described in the key.

Once we identify that this woman may have started her ANC late in the third trimester and has been identified as HIV-positive, we categorise her as (A, meaning she is) high-risk. Then as a healthcare worker, we should tick on that column where we will be given or just put the letter. Or the column should have a variable already indicating that High risk, then we can circle. That is what we have discussed.

Chair of the day: Should we ask questions immediately?

Team: No

We hope we are writing the questions, and we will ask them later

## Group3

Our discussion is as follows: we need to add a few columns only in the maternity register. However, we have noticed that there are already existing columns in the maternity register that are already addressing these issues. For example, new positives exist, meaning the woman recently received an HIV test during maternity. And those who started ART in the third trimester already exist. So, we are only adding aspects not in the register. So, what we do not have are the following. Add a column for recent viral load results to document a current result. Whether it is low or high, we need to be recording. The recording will help with the classification. We also do not have treatment interruption in the maternity register, and it's necessary to add. However, we felt that treatment interruption would be based on the ANC period, and we thought it would be easy for nurses offering ANC care to note, so we could use the ANC page in the health passport book where they ask about adherence. So, this could be recorded. We also noticed that in the ANC, there is already space where they ask about adherence issues; we could use that space to document information like this. Apart from that, ART interruption can also be noticed on the ART card. It might be involved for an ART nurse to start looking for the cards to check adherence. We also decided to document the woman's adherence to the health passport book. If we include all that we feel, like all the questions we ask to classify a woman, we will have them. But we have suggested further adding another Column in the maternity register that will now conclude whether the woman is a high risk after the health care worker has completed asking the questions.

After identifying whether the child is a high-risk infant, what treatment are we giving? We noticed that our maternity register already has a column for NVP. Still, we need to add 2P close to where there is NVP to note whether the correct treatment was given to the infant according to the classification.

Chair: Thank you very much. Let us clap for the group

## Group 2

We did not spend much time. we just demarcated the columns

Team (Laughing)

Do not laugh

Our first column was on variables, and we discussed who would be responsible for to document

The first column should be for viral load results. With the new guidelines, we are testing women after undergoing treatment for six months to determine their viral load results. This column could be added to the maternity and ANC registers so we can capture them when they come to the ANC. So, we will put viral load results as a variable in those registers to capture that information. Nurses should be responsible for documenting these columns because they provide the services.

We cancelled something here, but we should present it to you. We include the date when the woman was initiated on ART. We cancelled it because we remembered this was already captured in the maternity register. If the woman has tested positive, we document it in the maternity register in line with the age of the pregnancy, for example, if the woman was tested at 28 weeks and so on. Similarly, in the ANC., if we have circled in the labour ward, it is already an indicator that the HEI is a high-risk, so we think that one the maternity register already has, and we leave it the same way. But at the end, as another group has suggested, we need to add a column for 2p as we only have space for NVP, and the nurse will be responsible for documenting because they are the ones handling the patient.

If you have any questions, the whole group will be responsible to answer

| Individual |
| --- |

Of course, the issue of adherence is already there in the maternity register, but we still thought we might need something to guide us and … will clarify.

## Individual

Of course, we have not yet started the questions. So, it's now time for questions. Let's start with Group 1

From what the group presented, do we have any questions or comments?

## Individual

Of course, I need to understand group one's mention that they would have letter numbers. Still, I noticed that some of the registers already have the letters. Who is responsible for writing the letters to reflect the changes if they are added since the log was created?

Chair: Group one, are you able to understand the question?

## Individual

Because the registers were already created at the ministry, it is impossible to say change today. Our thinking was that we could improvise and that we were there now. We should improvise and start. Being very clear that we will put this letter, this is what it means.

## Individual

That may affect the reporting since we currently follow the variables and the letters when we compile the report.

## Individual

Please note that these letters or numbers are specific. Currently, this number for variables ends on the 31st, or what number again?

## Individual

it ends ending 43

## Individual

Yes, it means whatever we are adding on improvising will start from 44

## Individual

When we document the variables, if we improvise, should we also effect the change on the reports?

## Individual

Yes

## Individual

Won't that affect the electronic reporting DHS II because we do not have those included?

## Individual

What is essential for us to understand is that what we are doing here is just improvising only for this study. For example, the other group has suggested adding other columns. For this study, we have been given permission or provision to assess the variable we may include to categorise the women and the infant just for this study. That means that since we are evaluating these, it is an opportunity for our use. We will not include these when reporting. But you must know how you performed for Urban and rural facilities.

## Individual

Yes, that was my question: if we include these in the report?

Team: For our own sake, urban and rural, we will analyse at the end of the month

## Individual

no, because these are not there in the DHS II because we are doing this for our own sake to understand

## Individual

But for example, the A-one is already there in the DHS II

## Individual

What does the A category state?

## Individual

women starting late in ANC

## Individual

For every woman attending ANC, we categorise when they have started following the ANC, whether it is before 28 weeks, and we also have another column to document if the woman is on ART.

## Individual

That is why we have included two options: letters and numbers. If, for example, we have put the woman as high risk, we thought it was necessary to show why the woman was categorised as high risk. Was it due to defaulting treatment or starting ART late, for example? That is why we included that and added the key. The person documenting that will consider what the letters or numbers mean. For example, the 2 represents this, and the A means that.

Individual: I have a comment. As much as we are improvising, we also need to have the thought that if the issue has shown to work, we can scale out these. I want a clarification. Does it mean the codes the groups have suggested are already in use?

## Individual

The numbering system, yes, is already there up to 43. What is your suggestion? Should those codes start from 44

Team: Yea

## Individual

yes, because code 1 is already there, and so we do not want to duplicate

## Individual

Do we have another question? From their presentation, it seems that some areas are already captured in the current registers, but not all.

Team: yes

## Individual

So, what is not currently captured is the Viral load and 2P.

## Individual

Also, note that the coding should assume the numbers only from 44

Chair: Do we have another question?

Researcher

Should these two additions be added to all registers?

Individual

ANC, MARTENITY, POSTNATAL AND FAMILY PLANNING. Group one suggested, so what do you think?

## Group1

That is what we felt that should be the case because it will be easy and a reminder to focus on these at every point of contact

Researcher

So, if you are reporting, which will be your data source?

## Group1

The data source will be the registers we used to capture the data.

Team. So, does it mean that you will use all the data sources?

## Individual

to clarify this. If the same woman went to FP and then immunisation, you also captured her during ANC. Which data source will you use now when you want to develop the report? Does it mean you need to get all the data sources?

Researcher

For example, what would you do if you would like to note how many women you have as high-risk women for Urban in a month?

## Individual

we are suggesting all this to ensure we do not miss any woman because if we put a specific entry point, there is a possibility that this woman may miss a particular point of entry that, in this case, may be the one that we have designated to capture the risk status. So, we want

Individual

There is another question from the team. Do we also need to add it to the HCC register?

## Individual

Our group reflected on all that and asked ourselves about the importance of collecting such information and the purpose of collecting such information. Now, we reflected on the need to collect such information in family planning, and we noticed that a woman comes for family planning six weeks after giving birth. If we look at the information we are collecting, it will not be valid for six weeks. The reason for classifying the child is that we want to be guided on the prophylaxis to give, which is not offered after six weeks. Then why should we include women in family planning

Individual

Yes, I agree we will not initiate the infant treatment, but it will give us a clue that we missed the baby, and we can work on finding out where we missed the infant

Researcher

I love that we can identify the woman at every point, but how will we handle duplication issues?

How will we ensure that the same child is not counted twice if we have multiple sources?

## Individual

They may be based on the names.

Individual

Let us give an example. If we use the ANC register, the name we Will document will be for the mother, right? If the same woman is a week after reporting to labour, she will be captured again and appear on the postnatal and maternity reports; that's where we are querying the documentation issue.

Individual

But if there is good documentation, the health passport book may show that this woman was already captured. The HCC number should also be documented in the mother's passport book because documentation is paramount.

Individual

From what I understand, does that mean that the nurses at the labour ward should look at whether the woman was already classified and should not include her in the maternity register based on whether that information already exists in the health passport book?

## Individual

That means that the woman will also appear in the postnatal report

Team: NO

## Individual

Maybe the woman can just be documented in the register but not be included when reporting

Individual

Or maybe we should add another column in maternity to indicate that the woman was already captured in ANC?

## Individual

The maternity register is the most critical register to include here.

Individual

We can have the nurse document in the postnatal register that the woman was interrupted and in the health passport book

we can write in the ANC that the woman interrupted and may have a high-risk child

## Individual

where is this documentation taking place?

Individual

They are stating that we are documenting the mother in ANC. While in postnatal, we will document the child

Team: laughing

Individual

No, at the postnatal, we document both mother and infant. That is why we have discussed the issues about handovers on the initiatives. So, the healthcare workers should pick "How many infants are exposed, etc.?"

Individual

I have heard that explanation, but where are we noting that the woman interrupted?

Individual

ANC

Individual

yes, that is why we are saying all the questions are for both the mother and infant

Individual

do we only know about the interruption in ANC?

Individual

No, but this woman has just given birth, and we want to establish whether she is a high risk or not, so the question about the interruption, we will check during her ANC since this will be our first encounter, and we will not about ART status from her pregnancy.

Individual

Those must be different codes. Are you telling me that we will also not ask about the infant? To determine and then initiate the infant on medications.

Individual

Okay, let me remind you that this discussion aims to make sure that we do not duplicate the same person that should not be counted twice

Researcher

If we also document in the maternity register, at what point will this be filled?

Okay, let us take one question that we use to identify a high-risk infant. Let us use what we do not have already in the maternity register

Okay, never mind my question. I think it will further confuse the discussion

, but why did we not consider the postnatal register

Team: the postnatal register is complicated and may not be a perfect register to count on

Individual

So, what will we do so that we do not duplicate documenting the woman or client

Team: we should consider documenting the woman only at one point of entry

Team: may be in the maternity register

## Individual

Should we only document the woman in the maternity register alone? And disregard ANC?

## Individual

In the maternity register, we can rely on the interruption of the ART variable to also learn the mother's ANC information because the nurse will know how the woman was taking her ARV by asking about that history during her ANC.

Individual

We are failing. Group Two

Team: no, we are not

Researcher

At what point will this be documented if we let this information be collected at the maternity?

Team: we document after the woman has delivered a baby, hmm when the woman comes to the maternity

Individual

What is on the ground?

## Individual

We document the register as soon as the woman has given birth

Individual

So, I want us to state what we do in our facilities. If we have a client and we have admitted them to the labour ward, don't we document them in the maternity register?

## Individual

no

No, we document only when the woman has delivered a baby

## Individual

or we document when we are referring to the woman

## Individual

The register we used to document every woman at admission was phased out.

Individual

There is a reason I am asking this. I am not just asking for the sake of asking

Researcher

I am also surprised by the responses

## Individual

okay, so a long time ago, we used to have an admission book and maternity register. So, in the admission book, we documented all admissions, including preterm labour and other conditions, if we were admitted at the facility. Now, those that have delivered, we were documenting them in the maternity register

## Individual

so, we document them in the maternity register if we are referring them or if the woman has given birth.

Individual

If Urban and rural, you can do that. I will not talk to more

coordinators. Have you heard what the team has described?

## Individual

Yes, but what we know ever since. If we admitted a client to the labour ward, we had three registers: admission, maternity and postnatal registers. In the admission register, we documented a woman we had assessed. We saw that she was not due to give birth but was put in the waiting ward, and we could generate an admission number from that register, which was later transferred to the maternity register. We were also documented in the postnatal register when the woman had delivered. But now, because we had register and resource challenges, we only have a maternity register. As such, if we have a woman in our ward, we assess her, and if the woman is not in labour, we document the woman on the admission sheet and have the woman wait in the waiting area. But when the woman has delivered, we now document the woman in the maternity register and if a woman has a complication. We are referring to QECH. We also document them at that point and refer them to QECH.

## Individual

I'm afraid I must disagree with that process

## Individual

It appears that the two sites where the study is taking place are handling the women using that process, so let us leave the way things have been explained if there are other issues coordinators will discuss s.

## Individual

I have this question so: assuming that we have a woman at the ANC and we notice that the infant she is going to deliver will be a high-risk exposed infant, and when we document the woman when she is admitted at the maternity, what do you mean that we are going to duplicate the infant as we want now to have the infant initiated on 2p

## Individual

That question is confusing. Is it following our discussion, or is it an independent question? We are discussing what happens when we document a woman in a maternity register.

## Individual

Regarding duplication, another member asked if we document the same woman at ANC and Maternity, are we not duplicating these clients?

## Individual

ahh, okay, so on the duplication, can we discuss how to fix that?

## Individual

So, that is why I asked how we are approaching to avoid duplication.

## Individual

So, from what we were discussing, the team was suggesting that if we identify a woman that will have a high-risk infant in ANC and then document in ANC, then later the woman gives birth, and we document in maternity that the baby is high-risk and initiate

2P, there isn't any duplication there.

## Researcher

Now, the question is also coming at the end if we are analysing our data may, be at the end of the month. If we want to check how many high-risk infants there are. Or even for our own decisions as a facility, which data source will we use? Then, a response said the facility would collect data from all those entry points. Then there was a question asking if the woman has been to more than one entry point, won't there be a possibility to duplicate?

For healthcare workers knowing on each programme that they had so many women with high-risk HIV-exposed infants, I guess that's okay, but if you want to have the picture for the whole facility, what will be the approach? Which register are you going to use?

## Individual

I think the best data source, in this case, is the maternity register

It's good for our programme-specific area to capture the high-risk data. I believe that for the report and to have the entire picture, we can have one to avoid duplication

## Individual

So, we have agreed that maternity is the best, not so?

Team: Yes

## Individual

Okay, let's improvise and add the columns with pens for the variables that are not there.

## Researchers

Please point out again the numbers where the registers stopped. Do we have a viral load again?

Team: (Several people explaining)

## Individual

Viral load at six months and establishing adherence. Group three noticed that the variable of asking a woman if she is on ART is already available in the maternity registers, which is why they removed it. However, the only outstanding items are these two viral load results and adherence, although adherence is not fully captured because adherence is subjective to establish. Because they tell you that I have taken all medication correctly even if they have not because they understand they will be given a small supply of medications. So, there are chances that this variable will give us false information. 1) we may categorise more as high risk, yet they are not or categorise missing some that are indeed high risk

## Individual

In addition, the focal person will need to examine the statistics of these variables. For example, how many women have high viral loads, and how many are reporting good adherence? We must know how the focal person can determine that they had such many non-compliant or non-adherent clients.

## Individual

Determining adherence was what was challenging in group three discussions, and that is why it was left hanging

## Individual

If we take the ART master card when we look at the pill count, missed doses like four plus pills. Doesn't that answer adherence?

## Individual

Yes, that is what it intends to answer, but what we are saying is very subjective because people now understand that if they are given medications today, they calculate how many tablets are supposed to remain with them and come with them to the facility and if they accurately report they know they will categorise that they have poor adherence

## Individual

of course, sometimes they still at least tell you correctly

## Individual

Of course, it is unfortunate that sometimes our clients give us false information. Had they given us the correct information, we would not have problems.

## Individual

How will we handle those saying that I did not come to my given appointment because I went to the village, there was a funeral, or someone was admitted to the hospital?

## Individual

Those are the ones that usually fall into the category of poor adherence

## Individual

I am sure we will still meet some who will tell us the truth, but we will still meet those who will give us false information that somehow, we pick through the appointment date and the pill count.

## individual

So, what do we agree? Do we still leave adherence as the criterion?

## Individual

I was asking about the VL issue for the person who started ART some time ago but does not have a viral load. How do we handle that person when categorising the infant?

## Individual

Policy person member (Phone call)

As in the routine ART programme, we will inquire from the clients. We ask for a pill count, and we take it from there. The objective way of knowing about adherence is through the viral load. Still, if we do not have it, we know that asking about adherence and verifying medication uptake is critical.

## Researcher

So, can we confirm non-adherence subjectively?

## Individual

Yes, that is what we even do in the ART program. When any client comes, we ask them what they are remaining with. If using the J2 it is even better because the J2 helps calculate how much the person stays with. It also produces a report on whether adherence is expected or below, so it is essential still to determine compliance. The objective way of knowing about adherence is through the viral load. Still, if we do not have it, we know that asking about adherence and verifying medication uptake is vital.

## Researcher1

We asked the policymakers to ensure our actions align with their recommendations. This means that we will still be based on the subjective reported information, so according to this information, it is still essential to ask about adherence, and we need to maintain it. However, the information we will get is subjective. So, from the questions, we will need three variables. Viral load, adherence and interruption

## Individual,

Do we need to add other variables?

## Individual

There was also a mention of 2p.

## Individual

So, should we include 2p in the register? Because of the master cards we are using now, I think they have 2p, but not so.

Team: some of us have seen the pink cards:

## Individual

oh, so the pink cards are out. What I have seen is the reporting form

The team has informal charts

## Individual

So, group three, we are done?

Of course, we have included a column for adding 2p in the maternity register and ANC register.

Member: now that we give Niverpine during ANC, do we also offer 2 P during ANC?

Team informal charts: we can't give them in advance. Others suggested that it should be given

Individual

2P, we should give in advance the same way we give prophylaxis for Niverapine. We also need to give 2P if we think the child will be a high risk

## Individual

okay, as much as NVP, we give in advance, and we give education according to body weight. Does it mean that if we have administered 2p and the baby is underweight, we cannot give NVP? Does it mean that we will withdraw? I am asking because there is still a lot of knowledge gap as a lot have not been trained in the new guidelines about 2p, and being able to withdraw, will that be effective?

## Individual

That is true; we are supposed to give in advance, and if the baby is born as my colleague mentioned, then we need to verify the weight

## Individual

Let us give the prophylaxis in advance, but we will advise them not to provide the medication until advised at birth.

## Individual

Now, we are not trying to change guidelines. For something universal for Malawi, and if it's not part of the intervention, it needs to be done as it is. If people are recommended to get nevirapine before delivery, 2p must also be given.

## Individual

Similarly, Niverapine is according to body weight, and we give the actual instruction when the baby is born.

## Individual

We instruct that the mother should keep the medication for the baby, and we provide the education. Still, actual administration during birth happens when the baby is born. Both prophylaxes are weight-dependent.

## Individual

Do we have a column for prophylaxis in the Maternity register?

Team informal charts its already there, but there is a need to add 2p

## Individual

Because we are determining what we give the woman during pregnancy, we feel the ANC register should include an indication of whether the prophylaxis we are providing is NVP or 2P. Do we have any more questions?

## Researcher

Yes, I have one question. Someone commented that there should also be a provision to document adherence status in the health passport book.

Team: It is already there

## Individual

So, does it mean you just added it there? It is already there, although it is subjective

## Individual

In the health passport book, it asks if the mother is on CPT, and we say yes, let me finish,

but before circling yes or no, we are required to ask how she is taking the medication because if she is not taking her medication, we are not supposed to ask

## Individual

Now, I am looking at what happens on the ground. How many people ask for that information? Now, because we do not adequately circle, if the woman is not adhering, there is still a need to comment on top of that page about poor adherence. Because it is true that in the book, there is provision for that, but it does not always happen as you say it happens

## Individual

That is why here we are discussing that we should reflect on the identified gaps when we go to the sites to teach healthcare workers that we need to highlight the problems we are discussing

## Researcher

But also, this we will share with the M and E officer. Maybe it may guide the upcoming M and E development materials

## Individual

I think for this activity, we are done

## Researcher

Can I conclude what we have discussed to verify if what I have captured reflects the discussions? We have agreed that some of the questions we use to assess if an infant is a high-risk exposed infant are already available in the maternity register, and we do not need to add variables for those. We are only adding the variable for adherence, which will be asked by collecting subjective information from the mother. This will be based on the pill count, appointment date and the information given by the mother, and it will be up to the healthcare worker to decide. We have further agreed that we will add some columns for ART interruption, viral load, and actual classification of whether the child is a high-risk infant. Is that what we are all agreeing on? And so, should we include the code you mentioned or not?

## Individual

You should also add the column for 2p

## Researcher

Okay, in the column for 2P, the best variable could be prophylaxis, so the one providing services should just be writing. But since we will be using the current register, there will be a need to include a 2p column. But for future registers, there will be a need to just put the variable for prophylaxis

## Individual

Including the antenatal register, as well

## Individual

We are done with this item, but next on the agenda, there is a need for us to discuss data management

## Researcher

This is what we are just finishing now, not so.

## Researcher

The other one is linking with the referral facility. So, is going to lead, but since she is not around, we can go to the sustainability:
