# Supplementary material for: Developing an intervention to improve early infant HIV diagnosis service uptake among postpartum women in Malawi’s primary healthcare using a co-designing approach with stakeholders
Source: PLOS Glob Public Health. 2025 Apr 22;5(4):e0004426. doi: 10.1371/journal.pgph.0004426 (PMC12013899; doi:10.1371/journal.pgph.0004426)
Supplement: S1 Data — (ZIP) [file pgph.0004426.s008.zip › S1_Data/Formative minutes.docx]

**EARLY INFANT DIAGNOSIS SERVICES INTERVENTION DEVELOPMENT WORKSHOP IN BLANTYRE DISTRICT**

**Introduction**

In 2019, Suwedi-Kapesa conducted a study, " Evaluation of Early Infant Diagnosis (EID) of HIV Services in Blantyre Malawi", to assess how early infant diagnosis services are provided in the district. In 2020, the study's findings were disseminated to the extended district health management team (DHMT) members and other key health workers from the study facilities, and a copy of the presentation was shared with the Prevention of Mother-to-Child Transmission (PMTCT) Coordinator.

Having learned about the challenges of the EID program from that study, we engaged stakeholders in a formative workshop to validate the problems and develop solutions.

**DAY: 1**

**DATE: 5^th^ August 2020**

The meeting was opened with a prayer.

One coordinator chaired the meeting, and there was a self-introduction. This was followed by welcoming remarks and a request that all the participants be active and attend all workshop sessions. The workshop logistics and proceedings were explained, and any concerns were accommodated before starting. The objectives of the meeting were outlined.

**Objectives of the EID Intervention Workshop**

- The main aim is to develop an intervention that addresses key challenges in the EID programme for up to six weeks.
- Overview of EID study
- Validation of the findings
- Analysis of EID challenges
- Solutions
- Literature of EID intervention
- Com-B model
- Application of the Com–B model in line with knowledge and experiences
- Reflections

**An overview of the study findings per summarised slides**

**EID challenges faced in the facilities**

Following the presentation of the study findings, we had group discussions, and stakeholders discussed whether the findings portrayed their experiences in the sites and went further to add additional challenges summarised below:

- Negligence of health workers to enrol HIV-exposed infants in HIV programs at birth
- The assumption that women who receive HIV care at another facility will have their infants enrolled at that facility, despite guidelines stating that they must be enrolled and discharged if their mothers receive ARVs at another facility
- Knowledge gap to some Health Workers.
- Lack of coordination.
- Lack of privacy in rooms and leaving files in the hands of the clients.
- Distance leads to women reporting late at the facility.
- Lack of knowledge by the clients.
- Knowledge gap between the support staff and HCW.
- Lack of integration of services required by both the mother and infant.
- Poor documentation and filling.
- Fragmented Infrastructure in facilities.
- Turnaround time of results for facilities that have no Point of care machine (POC).
- The default rate is high due to self-transfer, especially in urban.
- Those trained in POC machines, some are not HIV counsellors, and women are offered HIV testing for their infants without counselling
- Regular checkups at 6weeks were stopped
- There is a poor flow of clients from under 5 to HIV care clinics.
- Late provision of HIV care services to clients at the facility.
- In return, clients report late to the health facility to refill their drugs.
- Bad attitudes of health workers toward clients who are HIV positive during deliveries.
- Health workers refuse to provide services to clients when they realise that one is HIV positive during childbirth.
- 13% of Health workers are not trained in EID in BT.
- When new standard operating procedures for managing HIV-exposed infants are released, health workers are not oriented.
- Health workers in the facilities shun away from EID mentorship because there are no allowances.
- Stock out of registers.
- Failure to identify women with HIV-exposed infants at the under-five clinic or outpatient department.

After discussing the above-said challenges, the group suggested the following solutions without first reflecting on the theory.

- Training of providers to offer integrated services.
- There is a need to train health workers on EID.
- There should be a way of harmonising mentorship within the programme and partners.
- Facility-based orientation on EID.
- Timely requesting of registers at facility and district level.
- Intensify health workers to enrol HIV-exposed infants in HIV care
- Identify key people to supervise and lead the implementation

NB: Partners highlighted that they are already using phone calls and SMS to remind people to come for their appointments and call those who have missed their appointment

**A question during the discussion: do we have a focal person? If yes, what are the qualities?**

They indicated having an EID focal person rather than a PMTCT focal person and considered the following attributes for the EID focal person.

- They should know about the programme.
- They should be able to manage to write a report.
- They should be able to manage to analyse the data.

NB: The EID focal person is usually a health surveillance assistant who mainly follows up if a child has been tested and there is proper documentation in the registers. They also lead in compiling reports for the program. They are not a nurse and do not work in the labour ward, and it is challenging to monitor the enrolment of an infant in HIV care. He is usually a person in a low cadre as compared to nurses and clinicians who play a more significant role in the management of an HIV-exposed infant. They indicated that all the focal persons were appointed based on their performance, and there was no formal training.

They indicated that they currently do not have a PMTCT focal person who can oversee the entire program from the child's birth who is supposed to be trained and a qualified PMTCT provider. Who can either be a nurse or clinician

- Enrolment should be done at maternity with nurses and supporting staff.
- Screening for postnatal checks at 6weeks on EID should be done at maternity and under-five clinics.
- Negligence should be addressed by having supportive supervision and mentorship and conducting monthly staff meetings led by the PMTCT focal person, not the EID focal person, as there are power dynamics.
- There is a need to allocate qualified nurses on duty if the focal person is not available to lead in addressing the challenges of staff on duty.
- The challenges of service integration: some services should still be provided in one room, like EPl, ART, FP and EID or close by rooms.
- Check health profile at every point at OPD maternity and under five.
- Wake up the PMTCT programme in the health centre by ensuring active coordination as it is dormant.
- There should be in-service training at the Health Centre on pink files and registers. In addition, the orientation of all new staff at the facility.

**Sustainability of the programme**

Participants came up with some factors to help sustain the programme the group discussed.

- Those trained should brief the rest of the staff because the partners cannot train everyone.
- One to one counselling is very important and can help to sustain the programme.
- Privacy is also essential; the client should be involved when doing everything.
- Give adequate information to the clients before discharged.

**DAY 2.**

**6^th^ August 2021**

The second day started with a word of prayer. **Then, the behaviour change wheel COM-B model guides the thought process for deciding what behaviours need to change. In summary**

**Behaviour change wheel COM-B model**

C - *capability*

O – *Opportunity*

M - *Motivation*

B - *behaviour*

- **Behaviour**

People must have the capacity, opportunity and motivation to perform a behaviour.

- **Capability**

Capability makes one engage in a particular behaviour.

- **Motivation**

These are fundamental drivers and automatic processes.

**How to change a behaviour**

- There should be an analysis of the determinants of the behaviour.

We also went through a wheel that explains behaviour change, and after a long discussion, we broke into groups.

We worked on defining the problems. We looked at what is recommended for health workers to do in their service provision according to the Malawi PMTCT guidelines. We explored to understand what they were not doing based on their experiences and the study findings. The focus was on defining the behaviours that needed to change, and below were the identified problems:

- 1. Not enrolling HEIs in HCC
  2. Not offering HIV test to HEIs at 6 weeks
  3. Inadequate coordination among the health workers
  4. Inadequate HIV testing counselling and documentation of test results

We worked on identifying and specifying the target behaviour. At this stage, we looked at the list of behaviours listed. We explored which behaviours we could target by examining what the behaviours depended on as barriers to the desirable behaviour, as outlined in **Table 1.**

**Table 1. Health workers target behaviours to be changed and barriers to optimal behaviours**

| **Identified problem** | **Identify target behaviour (barriers)** | | |
| --- | --- | --- | --- |
|  | **Capability barriers** | **Opportunity barriers** | **Motivation barriers** |
| 1. Not enrolling HEIs in HCC | 1. Inadequate knowledge of enrolment of an HEI in HIV care 2. Inadequate knowledge of documentation and filing | 1. The assumption is that women who receive HIV care at another facility will have their infants enrolled at that facility 2. Unavailability of under 24 months’ cards and HCC registers at the postnatal ward 3. High workload when there are many births | 1. Negligence among health workers who feel the work will be done by someone else 2. The attitude of health workers to the client who is HIV positive during deliveries   Improper |
| 2. Not offering HIV tests to HEIs at 6 weeks | 1. Failure of health workers to identify HEIs among postnatal and under-five clinics  2. Inadequate knowledge of documentation | 1. Poor flow of care of clients  2. High workload that sometimes is increased by health workers' shift arrangements  3. Returning clients home without HIV test after waiting for PoC HIV testing (other days, there are more women to be tested for PoC than the machine can handle, whilst other days, there are entirely no women to test  4. Specific HIV and EID clinic days and time  5. Assumption that women who receive HIV care at another facility will have their infants tested at that facility | 1. Increased waiting time for clients, who are prompted to leave the facility without other services like HIV testing 2. Health workers reporting to work late |
| 3. Inadequate coordination among the health workers | 1. Undermining the abilities of the EID focal person, who mainly was a health surveillance assistant and was not directly working in the labour ward and was of low cadre to nurses and clinicians who usually enrol HEIs in HCC | 1. Provision of fragmented health services due to infrastructure and poor flow of care | 1. Health workers feel HIV care for PMTCT women and infants is only for people who are in quality improvement teams 2. People in quality improvement teams only meet to discuss their projects when there are allowances to support their meetings 3. Health workers stay away from EID mentorship due to a lack of allowances |
| 4. Inadequate HIV testing, counselling and documentation | 1. Inadequate training of HIV testing services councillors (HTS) on the use of PoC machines 2. Inadequate training of lab assistants who test HEIs with PoC on HIV counselling 3. Poor filling of records | 1. Moving around cards and registers when providing care 2. Stock out of registers 3. High turnaround time for results for facilities that do not have PoC |  |

3. We worked on identifying what needs to change and specifying who will perform the target behaviours, what needs to change, and where and when they need to make the changes. How often and with whom ? Therefore, the health workers in their group expressed the following key elements to be changed:

1. Active patient tracking (using identifiers/ a label from the Labour ward and providing information to the mother on where to report for subsequent visits to identify a woman at first encounter. Then facilitates that the mother-infant pair receive all services without being in a queue for each service.
2. A booking system for HIV testing for infants in facilities that use point-of-care machines to avoid increasing waiting time and turning clients back according to the machine's capacity for each day.
3. Strengthening leadership in the management of mother-infant pairs through the introduction of a trained PMTCT focal person. The facilities previously had an EID focal person who was primarily a health surveillance assistant (HSA), who was not working in the labour ward and was of low cadre to nurses and clinicians. The EID focal person would have challenges understanding implementation gaps in the labour ward where HEIs are enrolled. Furthermore, it was difficult for nurses and clinicians to be led by a staff that does not work in their ward or clinic and is of a lower cadre to them. The need to strengthen the leadership is not replacing quality improvement (QI) teams that work on different quality improvement projects in the facilities but will further support effective meetings of the quality improvement teams if the teamwork of all staff is improved. Health workers expressed that most QI teams do not meet in the facilities without funding to support their meetings. This is because the QI teams were initially given allowances and refreshments to support their meetings.

The health workers and members of the management team present during the workshop showed commitment to support the facilities with periodic supportive supervision and request reports from the facility in charge, who will be working hand in hand with the PMTCT focal person whom they proposed that it has to be a qualified trained nurse working in labour ward/postnatal clinic to mentor other health workers, monitor documentation, and lead in facility meetings to review performance or encourage the QI teams to work on significant challenges that are being faced. They highlighted that negligence among health workers is a big challenge that may be solved with good leadership and teamwork.

1. Introduce data validation daily by the already existing staff/ to be monitored by the PMTCT focal person supported by the in-charge or any nurse assigned on the task allocation utilising hand-over meetings.

After examining the suggested changes, we examined the interventions that have been implemented from the literature to give stakeholders insights into what has worked elsewhere. These include the use of instant text messaging (SMS) to remind mother-infant pairs of appointment dates, mother-infant pair (MIP) clinics, restructuring the flow of care, provision of centralised care, active screening, and quality improvement initiatives.

One of the partners at the study sites communicated that they are already supporting the facility with Airtime to send messages to women who have missed their appointments. They also deployed some expert clients to assist in providing health education to clients who have defaulted from care. Therefore, health workers suggested that from the interventions presented, they would also opt to include screening at the under-five clinic, and they would first be looking for the label that will be indicated at the labour ward, whose details are laid out in **Table 2**.

4. After the workshop, the researcher worked on identifying intervention functions, behaviour change techniques, and delivery modes.

**Table 2. Specification of the change, who to perform, why, when and where**

| **Aim** | **What** | **Who.** | **Where/When** | **How to identify the actors** |
| --- | --- | --- | --- | --- |
| 1. Active client identification and tracking | 1. Indicate unique labels or identifiers in health passport books for PMTCT women and actively screen for the identifiers or HIV status at each encounter with any client  2. Providing information to the mother-infant pairs on where to report for subsequent visits after birth (nurses’ office at the postnatal clinic)  3. Once identified, channel the mother-infant pair to the nurses' station to facilitate delivery of all the services required by the mother, including offering an HIV self-test if the status is unknown and referring for confirmatory if positive to continue with the delivery of care | All nurse-midwives, clinicians and support staff | The antenatal and postnatal clinic, labour ward and under-five clinic | Meeting and training with all the staff |
| 2. Improve motivation of health workers, teamwork and compliance with PMTCT guidelines through strengthened leadership | 1. Placement of under24months cards and HCC registers at the labour ward, 2. Development of task allocation on a shift   3. Improved handovers with verification of documents,  4. Equipped PMTCT focal person,  5. Support from the DHMT and health centre management | 1. All nurses and support staff  2. PMTCT focal person, Nurse in charge or assigned nurse  3. All nurses  4. Trained Nurse midwife  5. DHMT members and health centre management | Labour ward | Meeting and training with all staff and consulting nurse in charge to select the focal person with the support of the PMTCT program coordinator |
| 3. Booking system for POC HIV testing | 1. Make available a book for booking POC HIV testing  2. Assign a date for HIV testing in line with other services not exceeding the capacity of the POC on a particular date | All nurses and support staff | At one-week postnatal check-up | Meeting and training with all staff |
| 5. Improve data management | 1. Daily improved handovers at the labour ward with verification of documents  2 Daily data validation of HEIs enrolment and HIV testing at 6 weeks.  3 Back up computers for backup data entry for the study | 1. All health workers 2. ART data clerks, PMTCT lead person and all nurses 3. Research assistants | 1. Labour ward, ART clinic  2. Labour ward and ART clinic during the study period | Meeting and training with all staff  Training research assistants |
