# Supplementary material for: Developing an intervention to improve early infant HIV diagnosis service uptake among postpartum women in Malawi’s primary healthcare using a co-designing approach with stakeholders
Source: PLOS Glob Public Health. 2025 Apr 22;5(4):e0004426. doi: 10.1371/journal.pgph.0004426 (PMC12013899; doi:10.1371/journal.pgph.0004426)
Supplement: S1 Data — (ZIP) [file pgph.0004426.s008.zip › S1_Data/Refining transcript.docx]

# Refining patient identification and tracking

**NB: We anonymised participants and their positions to protect identities in conversations. All group comments are labelled as group views, while individual opinions are labelled as individual, representing varying perspectives.**

## group 1(Presentation)

This focuses on women and HIV-exposed infants from birth to six weeks.

We focused on unique IDs for women living with HIV, where healthcare workers need to document a unique label in their health passport books to identify these women quickly. We already know that previously, we used to write PMTCT, and women used to remove these pages as it somehow discriminated against these women for others who could read their health passport books. So, we need to discuss this further because the unique labels may lead to discrimination.

Another point is on assessing women living with HIV to identify infants at a high risk of acquiring HIV infection. On this point, we have not evaluated these women mainly because most healthcare workers have inadequate knowledge despite the training being done. This should be intensified during the training for this intervention so we can start implementing this.

On the prophylaxes, we need to intensify that it is not only NVP; there is also 2p. Mainly because most people are not implementing this due to the problems that we indicated in our EID experiences

On identifying and tracking women and infants exposed to HIV through handovers during reporting. We agreed that we need to make all the changes. We agree on what was discussed in the formative workshop to help us identify and track women with infants exposed to HIV.

On measures in place to identify infants exposed to HIV at the weighing area, it had several points on what healthcare workers should do, and we felt all these were important. And we agree with all these.

Slide 38 focused on checking HIV status in all health passport books for all women. So, we further clarified that only trained people can distribute an HIV self-test kit. We have agreed that we need to sort health passport books for infants according to age.

On slide 39, bullet number two, on weighing infants, we felt that this needs to be intensified because we even need the infant's weight to give the prophylaxes.

Slide 40: we all agreed to the contents, and we did not change anything

On slide 41: the bullet number needs to be intensified for the healthcare workers to implement

## Individual

I want to add to the issue of patient identification. There was a suggestion that healthcare workers use PMTCT as a sticker to identify the women. We felt that it might bring problems as most people understand it, and when they see it, they usually know the HIV status of the person. And already, for the women that are HIV positive, if we write that on HAART, they remove that page even on the page where we document HIV status where there are two ticks they usually remove because they do not want to be identified. In addition, they sometimes change books because they do not wish to be identified, so as a group, we thought we needed to discuss and find a way how best health care workers could identify the women. We felt that we need to think critically to avoid women feeling that healthcare workers deliberately expose their HIV status by learning from previous experience.

## Individual

I want to give an example that we use at the nutrition services. We use. So, the same letters, for example, Z for those that are HIV positive and those that are HIV positive, we use numbers like 1. So, the same might apply where we can put a code and then a symbol that we can agree as a program what to indicate if positive and what to suggest if negative than using the words HAART and PMTCT. Learning from the nutrition services, we know that if one is indicated, the person is positive.

## Individual

The new symbols may work. Otherwise, People are already used to the word PMTCT meaning HIV positive. And they go and delete. So, this needs to go by the facility. So, for example, what can the urban facility decide to use that the staff will agree, for all the staff to know? It should not go by the entire district.

Informal charts

## Individual

This should not be decided at a facility because many clients move across the facilities, including the referral hospitals. If the idea is to identify and track the women, then this must be known to all healthcare workers regardless of the facility.

## Individual

The symbol should be decided at the district level. For example, if someone accessed health services at facility A, then they have gone to B, and if, for example, they are using -1, there will be a need for healthcare workers to have a common understanding of the -1

## Individual

The -1 won't, people, think it – is talking about the status.

## Individual

It is just an example.

## Researcher

Do you mean it can be anything people can decide, a number or letter?

## Individual

I want to give an example to support that the symbol should not be decided at the facility level. There was a patient that had different meanings at different facilities. For a long time, when HIV was not common, one NGO was using numbers, and various facilities were deciding their own. One facility used any number below 5 to mean positive, while another used any above five to represent positive. At these two facilities, this client's status was changing and did not reflect the client's status, but this was because they had a different understanding. And there was mismanagement.

## Individual

What do you write for nutrition services?

## Individual

The guidelines for nutrition services are apparent and instruct what to write if one is positive or negative. So, the negative one is (…) (this has been removed for privacy), the positive one is (…), and the exposed to HIV but not confirmed is (….)

## Individual

So, the clients do not know about those symbols?

## Individual

They do not know those that know are the healthcare workers.

## Researcher

Let's discuss this further. Is this necessary for us? Or why are we suggesting the ID? I hear you are stating that women change books, right?

The book a client has used this time may decide not to bring it again because they have specific books for specific services. One other reason why the symbol may be necessary is for healthcare workers to avoid asking the women in the queue about their HIV status. so, if that symbol is not available, that should already give a clue to the healthcare worker to put aside those books to explore at a better place in detail if there are other books at home or if the woman has never received HIV testing.

It may also be essential to consider who should look for that symbol among healthcare workers. We have said that health surveillance gets in touch with them, mainly with these infants when weighing. Could they look for that symbol? We must also reflect on what happens if women know that symbol because healthcare workers may publicly mention the sign, which may quickly beat the system.

So, it is up to us. So, should we leave it that facilities should discuss?

## Individual

We are learning from what the nutrition services do, and having such symbols in the guidelines helps. I suggest that we agree and share because if we leave it up to the facilities, we may not know how that feedback and scaling may work. We need to try to have it ready.

## Researcher

That is a very good point. So, where do we put this symbol?

## Individual

If we put it where HIV status is documented, people may relate easily.

## Individual

This information is aimed to be utilised by health care workers, right?

So why can we not put this information in front of the passport book?

## Individual

Usually, the first pages are not secured enough because sometimes the pages are lost, and the most preferred pages are those close to the middle. However, the middle is where the actual HIV status is documented.

## Individual

What about the (….) page? Where do we document the antenatal details of the mother? It is close to the middle of the book and has more information registered there, which may be difficult for someone who does not understand all that information. For example, there are details about hypertension, syphilis, proper HIV and delivery plans, and much more information. It may be difficult to note quickly.

## Individual

Hiding information?

## Researcher

What do the rest of us think?

Team: agree

## Individual

So, if we do that, what are we achieving? Is it that the mother should not know?

## Researcher

What is our primary goal?

## Individual

We do not want to miss any woman by any chance.

## Individual

That is true because even if the woman has come with a different book and if the healthcare worker did not find the symbol, it means the healthcare worker will have an interest in learning more in addition, if they found that the woman is living with HIV , they will document the sign without showing in the book in the manner that the client can understand that they are HIV positive. Ideally, the electronic system would work better. However, because of limited resources, we still want to have such information appear in their health profile books to help us manage them better and enable us to explore further if we cannot identify such information. So, the primary reason we need to identify. But if there are other suggestions, please bring them forward. Because at the end of the day, we need to ensure we can identify the clients

## Individual

Okay, now it means one person already knows about their HIV status, right? So, it means that the objective here is to identify so that any provider that may encounter that client should know about their HIV status. I do not know, but we have tried this as partners indicating something in the passport books. We have been stamping a logo showing PMTCT in the health passport book for the mother, which was serving as an identity. However, we were explaining to the mother about the logo and why we were doing that. Most women were okay with the initiative.

## Researcher

what were the outcomes of the subsequent hospital visits? Were they bringing the same passport books?

## Individual

Okay, when the woman knows about that documentation in that health passport book. That book will not be used again to access other health services.

## Individual

The women would say that they knew about my status. Mind you, they do not want to be recognised.

## Individual

Another thing is if I am assisting a client and do not see anything regarding HIV status in the health passport book. I would quickly look to see if there is a sign that we are talking about. And if I still do not see that. It will already prompt me to note the need to explore further on such a client. And if the status is unknown, we may explain to the client that she has a right to test after giving the woman information. On the other hand, If I stamp PMTC in the health passport and explain to the mother whilst I know that next time she is coming, she will bring a different book, will there be any progress?

## Researcher

Lately, we have seen an example in the study where, when we are looking for an ART number, they tell us that they are using a different book from the one they use when accessing ART.

## Individual

And others do not even remember what their ART number is

## Individual

Even now, with the new guidelines, we recommend knowing the viral load to assess risk status. Going on the J2 without knowing the ART number is quite different.

## Individual

It is also essential to know that with this initiative, the most important people we are targeting are those women who do not want to be identified or are changing their health passport books. Others can tell you properly about their ART history. But for this woman who does not want you to know, will that work for them if you tell them that I have known you and stamped in your book?

## Researcher

Let us hear everyone's input. Should we notify the clients that we have put a symbol that we want to identify them quickly to provide comprehensive care?

## Individual

They needed to know because we are doing this for their benefit. Once we tell them they are other people, they understand. In other circumstances, they may even tell you not to put it in this book because my husband is unaware of my status. So maybe we can consider what you suggest to those who do not want.

## Individual

How will the husbands understand the symbol?

## Individual

It would help if you did not think the clients usually hide their HIV status from us healthcare workers. They mostly do not want their fellow women, husbands, relations and even their workplace to know.

## Researcher

We need to know and understand that we are not meant to provide services without informing and seeking consent from the client.

This symbol will only assist in quickly identifying the client and tracking to ensure that we provide care. However, we need to explain to the client that care, including HIV testing, should not be a secret. Please think of the notes we write after providing services to the clients. Do we go page by page, reading our notes with them?

So, if we identify that there is no HIV status checked, we will still be giving information and enquire if they are interested in being tested without forcing. You will include that label for tracking so that when she meets another healthcare worker, she should not go through the same process. And by that, you are now talking to your fellow health worker that you met such a person, and there is something that you have already started doing about her HIV status. So, by this, we are not saying we need to be secretive and offer HIV testing without informing the client or enrolling the infant in HIV care without the consent of the mother.

## Individual

When testing people at the HTS, do you still use NR or R and circle it?

## Individual

No, we do not

## Individual

I think what we are discussing here is very important. We note women who do not have documented HIV status or because they have changed a book, and we refer them again to HTS, and they stand in the line, and that takes a lot of time in the process of providing the services. If we have that identifier, we can track the women easily and save time.

## Individual

The other reason to reflect again is when healthcare workers weigh the infants at the postnatal. Suppose that platform is used to identify these women. In that case, it will help reduce their time at the facility because nurses provide services on a first come, first serve basis. By the time they realise that the woman they are dealing with is mostly HIV positive, it is too late to have them go for testing of the infant. If there is screening even when weighing infants, it will be quick to ensure they are sent early to the lab. So, I feel it is one way of helping to identify and track the women and for the healthcare workers to coordinate and provide the services fast. I think we can use this ID in several ways, which is why we are bringing in the aspect of how best the unique ID could be used at birth, one week and six weeks.

## Individual

On the issue of HIV self-test, it is good that the group already reflected that we might not need to offer HIV –self-tests to clients already at the hospital, but instead, we can offer to test them and provide the self-test kits for the partners.

Researcher

I have another question for group one. You indicated that we need to intensify this. What did you mean to strengthen this? Like how we should intensify

## Group 1

We want the health in charge to support healthcare workers to check the babies' weight because we see most babies coming at one week without birth weight. So, the weight of an infant must be weighed and documented. Sometimes the healthcare worker checks the weight, but they do not document it. And sometimes, they discharge the infants after postnatal check without checking their weight. So, this needs to be reminded to ensure that healthcare workers check and document weight.

## Researcher

Should we consider all the points you have suggested that we should intensify, as in charge of reminding the healthcare workers?

## Individual

The other intensifying was on the assessment to find the high-risk infants. Because we learned during the discussions of our experiences that despite the new guidelines being rolled out and people being trained, only one health centre here is providing 2p to high-risk infants, so it is an important point, but we need to remind healthcare workers.

## Individual

There was also another point about the appointment dates for vaccination and HCC.

## Researcher

We have another group that they have discussed how to handle appointments, and we will pick it from there.

## Individual

I want to add to what another member has said about 2P. Others say they are not implementing because they have not been supplied with the 2P. When intensifying, we need to let healthcare workers know that it is the same 2 P that we have been supplying to HIV-positive paediatrics, and we will not have a unique formulation no. We will not have a particular supply.

## Individual

We had deliveries of the 2P in the facilities to ensure that it was adequate.

## Individual

Another facility said they never had the 2p, but when they learned of the new guidelines, they requested it, which was supplied.

# Refining patient identification and tracking

## Group 2

We did not make many corrections on slide 45, which also came, but we discussed it yesterday, and I will not go into details.

There was again another issue of providing self-test kits to women who do not know their HIV status. But we already discussed it yesterday. We also reflected that it may not be possible because not everyone was trained to offer HIV self-test kits.

Again, we feel few women might not know their HIV status, especially these days because HIV testing is intensified during antenatal. Hence, we thought those few women, once found, should be escorted to the HTS room to be prioritised to receive HIV testing than offering HIV self-test kits.

On page 46, during the formative workshop, they discussed the requirement for instructing healthcare workers to verify information in a health passport book on page 2. The information was to Assess for a high-risk status, book for HIV testing and prophylaxis, and enrolment HEI in HCC. So, we discovered that this information is unavailable in all health passport books. The information was only available in the blue books that the government printed, but now they are no longer there.

We recommend that the healthcare worker review these but may not find them on the suggested page.

On page 47: there was an issue requiring women living with HIV to be prioritised when they are accessing immunisation. The point was indicated that the nurse should advise the woman to go in front and meet the health surveillance assistant at the immunisation clinic. Therefore, we reflected that all health surveillance assistants cannot have a positive attitude if a woman asks them for immunisation without being in the queue. Or should we escort them for to receive the immunisation quickly? We are not sure what we think of the rest of us.

## Individual

The best may be escorting them. The maid or the expert client

## Group 2

That is what we thought rather than them going alone

We found pages 48and 49 to be kay

On page 50, we know that integration is quite a challenge because of the limited space and sometimes the providers. The situation is that providing all the services in one room is the best. What hinders us from doing this is space and limited health workers. So, to ensure that the woman does not miss other services and does not spend a lot of time at the facility, we need to intensify escorting the women from one service point to another.

## Individual

Providing all services under one roof is ideal

## Group 2

Yes, it is ideal, but most of the time, the challenge is space and healthcare workers. for example, if there is one nurse to conduct a postnatal check-up

, then do family planning, and then also check issues for HIV. With the high number of women requiring these services, it becomes too much and challenging for one nurse to provide the services. So maybe we can consider that wherever they are going for the other services, they should be assisted quickly

The last point was on counselling, where it is recommended to provide post counselling after one hour if using POC and six weeks if using DBS. So, we were discussing the standard time of expecting DBS results we learned that it is 14 days. So, we decided to include what is standard even though we do not usually have the results by 14 days.

## Researcher

And what is the duration?

## Group 2

Within 14 days

On slide 53, there was an issue of initiating the baby on ART if positive by taking the baby to the ART clinic. And we added that there is also a need to take a confirmatory test, which is the current recommendation.

## Individual

Thank you very much. That was group 2 reviewing the patient identification and tracking initiative at one week and six weeks. Do we have additions from the group members?

## Group 2

No, that is all

## Individual

Do we have comments from the rest of the members?

I have a question. There was an issue of escorting, recalling from the initial discussion during the formative, where there were suggestions that we should escort the women from one point of service to another. So, there were two issues that others raised then. 1. On privacy and 2. Feasibilities of escorting the women. For example, when nurses provide services, how easy is it for the nurse to find a support staff to escort a woman? And indeed, if the nurse has managed to find the support staff, how many women may be accompanied if we have managed to find six clients? Now I remember back then, people reflected on what was happening, and others mentioned that they gave the woman an HEI pink card to go with it to the lab for POC. Sometimes when the woman has gone for POC, the staff there may not even recognise that the woman may require other services. They end up testing the woman and inform her to sit on the bench to wait for results after one hour while the immunisation and family planning are running and closing close to the lunch hour.

The tracking was coming in to facilitate the woman's access to other required services like family planning and immunisation during the one-hour wait for results after the infant testing. Now, learning from that initial discussion, we need to reflect on whether it will be possible to escort all the women we will identify from one service point to another.

After refining this, we aim to train everyone at the facility. We may have staff turnover issues, but are we sure we will have others who will say they do not know? The other point to reflect on is the attitude of the HSA.

So, in this case, we have two options. Giving a woman a health passport book that we have documented the sign on the ANC page, asking the client to show the HSA, and taking the support staff to escort the woman, which one do you think is feasible?

## Individual

For the urban facility, considering the context. Escorting may be challenging. Maybe at the rural facility because they have a few women who are HIV positive with HEI. While at the urban, we may have about 8-9 women, and for the maid to escort all these may not be realistic. The best way is to advise the woman on the services she is supposed to receive, family planning, infant testing, and postnatal care. So, we may inform the woman that she may go for another service after testing the infant. It is like family planning because it takes one hour for the results to come out. Because most of the time family planning clinic usually finishes by noon. If the woman sits for one hour waiting for developments after lunch when she goes for family planning, she will be told that she is late and will be asked several questions about where she has been and may end up not receiving the method. So, the best is to advise the services they are expected to receive at the facility step by step. So, in the morning, nurses should take the PMTCT women aside and give them the information. But this will require these women to be assisted early at the postnatal for them to go with the infants for testing at the lab, and while waiting for the results, they need to go for family planning and then go back for results. Using the maids may be challenging, and they may sometimes leave the women on the way.

## Individual

I want to add that we must indeed come up with the ID we are talking about. It may help. What if the woman we are offering care to did not have the infant tested using POC but was tested using DBS, and the client must leave after testing? That means this woman still needs to access the other services quickly, so maybe the ID may be necessary for the healthcare workers to recognise such a person easily if every healthcare worker knows.

Individual

I think the nurse at the postnatal is the critical healthcare worker to have this process going. Because that will be the first place where these women will first report. And so, before providing postnatal care, the nurse needs to identify these women, and that ID needs to be included, and the woman needs to be given the information. Otherwise, if the nurse at the postnatal will not do this, the whole process will be messed up. I want to provide an example. Last month, one woman left the facility going home without the results because she felt that the results were delayed. So, should we say the woman was told she would take one hour?

Individual

As much as we have the ID, we also need the nurses to explain to the women to be aware of what is happening today.

Individual

I hope the note-taker is taking notes

Individual

So, what Is the way forward?

Individual

I think that we will rely on the identification and explanation and not the escort. Because this woman has come at one week and six weeks once the ANC nurse has identified her early, we need to include the label so that when the woman goes to access other services, she should be easily identified.

Individual

In other circumstances, relying on the ID alone without an escort is still tricky. If you think of how clouded the clinics tend to be, where will the client start to go to the front? Won't people question the client?

Individual

We agree that we will train the providers at the health centre in these two facilities. If the providers know these procedures, if the other clients accuse the client, the providers should be in a better position to respond on behalf of the client.

Individual

Reflecting critically, you need to note if the client was already within the facility and, for example, was reviewed by the clinician or maybe later referred for testing. When she is back, she does not stay on the same old line, and mostly they make a new queue with people that the clinician has already seen; now, they are queuing for a review. So, I feel it is quite the same if health care workers are oriented, and if there is that issue, the same explanation may be given that they were already within the facility and were referred for other services.

Researcher

I want us to be on the same page. So, in this group, you were uncomfortable with the client walking straight in front seeking services, right?

## Group 2

Yes, because it sounded as if the woman would walk to the front, and we thought even the HSA would be surprised why the woman was leaving the entire queue to go to the front.

Researcher

So, should we leave it and expand the item as people suggest?

Individual

Okay, but you still need to add that the ID should be used. The postnatal nurse can open that page and advise the woman to show the HSA that page so that it should be easy for the HSA to identify the woman and escort.

# Refining booking system

## Group 3

We reviewed the booking system, and the first point discussed during the formative was that the nurse or healthcare worker must document the number of MIPs to be tested on a particular day up to the number the facility POC machine can test daily. We decided to have nurses or support staff document the MIP scheduled for testing on a particular day and the number of infants to book on a specific day. Five might be a good number as much as the machine can test up to 7. Still, we reflected on scenarios where they might be infants that are HIV positive and may require a repeat test, and it may be difficult if we book all seven at once as the number may exceed, and sometimes the machine produces errors.

If we have booked five on that day and there are still infants, then we need to book them for the following day, and we found this point to be good.

Another point indicated when the booking should be made at birth, one and postnatal at six weeks. We agreed with this information, but we thought there was a need to consider transferring those from other facilities. For example, other facilities test HEI using DBS, and they may come to our facility without results, or some infants may come from another facility. At the same time, they are four weeks, and we thought the point is it is not fully covered.

We further added that for a facility that has no POC, we can book as many as possible because there is no limit.

Another point is that we reviewed a POC booking register the one used to book clients. The record has the following variables: name, HCC number, booking date, EPI appointment date, Mothers ART appointment date, Mothers ART site, serial number and POC appointment date,

So, we reflected that the serial number should appear before the other variables, just like how different registers are designed.

We reflected that we should include phone numbers for the clients to trace them if they were booked but did not turn up. We can use the phone number to verify her access to another facility.

We also suggested that we should add a column for comments. If the child did not come, we could put a comment that we followed up, and these were the findings.

That is what we discussed.

We are booking at birth. The nurse must have a booking register at the start of the postnatal clinic. We agreed on this point.

Document HEI in the booking register regardless of where the mother collects ARVs. On this point, we had a long conversation.

We thought we should only consider booking women who only collect their ARV at the facility and not book those who receive it elsewhere. But we also gave another example of one of the facilities where we are working. The facility offers a test to women from other facilities that provide ART but do not have maternity services. So, these women, although they receive ART at another facility, they have given birth at our facility. We also track them and offer them the HIV test at six weeks, and we transfer them out at six weeks.

And we thought it would be perfect to book these women and document in the comments section whether the infant will be tested at the same facility. So, we agreed to book the infant regardless of where the mother collects ARVs

Fill in the following variables, name, HCC number, booking date, EPI appointment date, Mothers ART appointment date, Mothers ART site, serial number and POC HIV appointment date, FP appointment data, and We agreed that all these are good.

If the booking date is complete, check with HSA and align the following appointment data to the EPI appointment date. Verify booking appointment capacity with the EPI appointment date. And change the booking date if the dates are different to ensure the dates are aligned. And we agree with all these as we have already discussed that we should aim at having the services provided together.

Complete booking means having five HEI booked for HIV testing, and we agreed that it is okay.

Aligns next appointment date with the mother, and we were in agreement

Booking at one week:

We agreed to the point where at one week, nurses need to verify if a six weeks appointment date was given.

Suppose was given check if it corresponds in the booking register and if it does not correct the date by following the steps at birth. We were also in agreement with the following.

If the next appointment date was not given, follow the steps at birth, and we agreed well.

Booking at Six weeks

Mark all tested HEI and give names to expert clients not coming for HIV testing. Transfer all pink cards to the ART clinic.

So, we did not add anything to the booking at six weeks.

Individual

So that was group three, and they presented on the booking system. Do we have any comments or questions?

Individual

I want to ask whether the maximum number of clients or HEI to be booked depends on the facility. Is the capacity of the POC five to seven everywhere?

Individual

I think this depends on the available POC machine to test the HEI. If the facility has one POC, then that number suffices. If the facility has two or more, that number will increase.

Individual

When you say machine, does that mean the gene expert?

Individual

The PIMA ones

Individual

So, the facilities here are only at C and D?

Researcher

We have A, B, C, D, E

Individual

But only C and D will pilot the intervention

Researcher

Do you have any suggestions?

Individual

I just wanted to understand the determining factors for how many clients to book in a day, so I guess it is the machine's capacity.

Individual

The considerations are that the sample processing time is one hour, and if the infant is HIV positive, they repeat and then consider working hours because it processes one sample at a time.

Individual

Why are we booking five? I thought we had other facilities that send their infants for testing, for example, QECH. Won't we put a buffer for those HEI? I think this will also depend on the health facility because, for C, we sometimes have no infant on a particular day, but we may have four or more on another day. So, I think it is important for the facility to look at the trend on how they offer the HIV test, but I do not think we should schedule up to five a day. So, A may want to look at how infants come for testing to determine the numbers.

Researcher

In that case, may we have a maximum number? As much as the facility may decide, we can set the limits by looking at the various factors. And we can state the factors? Such as the actual limit will depend on the trend of clients turning up.?

Individual

For me, I think five is still on the higher side because there was another day the machine kept giving errors for the first HEI and mind you, we need to note that testing does not start on the dot at 7:30 or 8 AM. because that mother goes for a postnatal check-up first. They may begin to go for testing after 9. so maybe we should put three a day, giving room for the walk-in from other facilities, and these that may just come, we can't say we should send them back.

Researcher

Whilst we are reflecting on the numbers. Based on the POC capacity, it is also important to reflect on the other services offered and that sometimes the facilities have specific clinic days. Remember, we aim to have these women receive most services at one appointment. I have also seen some omissions in the variables of the register. We did not include family planning.

Individual

I remember that when we were discussing the MIP, we saw that we only provide HIV testing and ART as much as we are thinking through the booking. We must consider all available resources, even for facilities without POC, with specific clinic days. So, all the women that we are booking need to consider that as a facility, we will manage to offer them all the required services the day we give them an appointment.

Individual

My worry is if we put five and we are sending back two. We should consider where they are coming from; sending the two back may not be good. Collecting DBS and having them come another time for the results is essential.

Researcher

I think it goes back to when we are booking. So, it appears we are booking at birth and one week. For those born at the facility, we can book them at birth. And this is not done at six weeks. At one week, healthcare will just be verifying to check if the date is appropriate, and few clients are booked at once for the six weeks.

So, if you have seven women at birth, for example, there is ample time to adjust the numbers, distributing them so that not all the seven come on one day at six weeks.

Individual

I think the challenge that may arise is doing this manually. There may be a mix-up of dates. Someone may duplicate the date, and we need to expect this unless we do this electronically so the system can note that the date is full.

Individual

It is true that an electronic system would help in those areas. But because of limited resources, we are still evaluating what can still work.

Individual

I want to ask the urban facility where they conduct their postnatal check-up/ is it the labour ward?

Because I am thinking of facilities with different places for conducting postnatal check-ups and deliveries, you may find that by the end of the clinic, they may book more than five clients.

Individual

Our facilities use the same building for deliveries and postnatal, so we will not have difficulties. Maybe rural?

Individual

We also use the same room.

Individual

I have a question: do you mean that even the facility that does not have POC needs to be booked? Are we booking to reduce women's time at a facility? If yes, then why book the facility without POC

Individual

I think a facility could agree as well because we need to remember that we are not only booking to reduce the waiting time or the time that the woman spends at the facility, but we want to facilitate that the woman should access all required services at a particular visit. Let me give you an example. At our facility, we provide family planning three times a week, and when we book these women, it is best to give them appointments on the days we do family planning.

Individual

And when do you do postnatal daily? And how do you handle those that do not need family planning? Does it mean you will give them an appointment on the day without family planning?

Individual

Yes, but we are also learning that all those services should be offered if one comes.

Individual

I would also like to agree that it is important to book even the facility where we do not have POC because the booking can also help to facilitate how many numbers of healthcare workers should be on duty to take the DBS to avoid having only one person who is taking the samples for a long time.

Individual

Looking from afar, taking the two facilities, urban and rural,

These two have specific dates. To some extent, we cannot say you should provide family planning daily based on their routine and set-up. But if they are boking manageable numbers, a postnatal nurse cannot fail to deliver those services to the few women booked. So, the booking we are using them because we cannot have all the available resources starting from human infrastructure and the POC machines, to offer everything in one place. So, our appointments should still facilitate the woman's access to all the required services. So, we have MIP, yes, but we have heard the experiences that the dates are not managed well. If we have manageable numbers of these women who require extra care, we may work amidst limited resources, and it may be possible for healthcare workers to coordinate.

Reflecting on urban, we do have a HSA that comes to maternity to offer immunisation, and at this point, I am sure the HSA already is giving out appointments for six weeks, or if they are not, I think it is easy for the HSA to discuss with the nurses and because they are already at one place. In that way, we are addressing coordination issues, having a lot of women in one day and even the resources.

Researcher

But it depends on our capacity and context., what works better for Rural?

Individual

Individual

We can manage. We do not have a problem. If there is one client, we can give immunisation, family planning, and HIV care.

Individual

Do you do immunisation daily at rural? Immunisation and family planning?

Individual

No, we do not do it daily.

Individual

Should you provide all the services when you book a client?

Individual

Usually, these women come on Thursday. We offer family planning and EPI services.

## Researcher

Okay, we assume we have trained healthcare workers and started the interventions. Our booking does not fall on Thursday, can we then offer all these services?

Individual

The best person to respond would be the nurse in charge because she must now know the nurse she has at the facility. If a woman comes on the day they have booked, will they manage to provide all the services?

Individual

Will it be possible? When responding, consider all the factors at your facility, the number of healthcare workers, how you can do your shifts, the EID program, etc.

Individual

Can I now respond? Our clinics will consider when the other services are offered. For example, we will not book a woman to come on a Monday because many things are happening at the facility. If anything, we will give them an appointment on a Tuesday because we know the woman can receive some services, including family planning, DBS, and EPI. After all, on Tuesday, EPI is offered. We already designated it as a MIP clinic on Wednesday and offered these services; on Thursday, we also provided many services, and Friday is for ART. The only difficult day is on Monday. It can't be possible.

## Researcher

I am listening with interest because all these show that the booking system can be used at the rural hospital because it also acts as a validation tool to check if all planned services have been offered and are reflecting what happens at the facility.

Individual

Now, a point was raised by one of our partners: what if there is an overbooking event? Can we not book the others on another day because they are coming from far away. At that point, I did not hear the conclusion. Because the woman can stay the whole day waiting that after that woman, they will test my child. Perhaps we can also have no electricity and still have the woman wait without testing

Individual

The way I know, the POC is already helping us determine how many women we can manage to offer the services to. And in these events, if we booked five and maybe two more have turned up when we know that with our resources, it will not be possible to test. It is important to tell them in good time and let them go than for them to come back. Once we know in the morning because maybe those booked are already in, we need to tell them, or else we can discuss with the staff at the lab if they feel they can be operating morning hours on this day.

Individual

Yes, even at F, sometimes we send back the women when we see the machine won't manage to test.

Individual

It is possible to avoid turning back the women, and the number should be three, as one of the members mentioned. We can book three at birth and expect two or three to come from other facilities rather than booking a maximum of five. And for those coming from the referrals, we will still accommodate them. Then, booking three is ideal.

Individual

Are we booking three because these facilities have a small catchment population?

Individual

This depends on several factors. I will give an example of an urban facility. Some days, the facility has no clients; others may have four at once. The number keeps changing, with some days being overwhelmed. If we split these numbers, we may have two or a better number each day. And we have this urban facility that we consider if it is a high catchment.

Individual

We are also reflecting on the capacity of the machine. Even if we have so many women. The machine takes one hour to process one sample, and then testing seven women means seven hours, and sometimes, when there is an error, it means restarting the process. It will no longer be at seven hours, and because other infants may be tested positive, they may need confirmatory testing, which means that one child would require two hours. And during lunch, the healthcare workers would also want a break.

Individual

But you do not go for lunch when you see a high workload.

Team: HMMMM

Individual

I do not think it is a reason enough to send people back because you are on lunch, especially when we know how the machine operates. Because one can take the sample and whilst waiting for the one hour one can have their lunch.

Individual

The way I understand the booking is based on the available resources because even if we have a facility with a large cohort requiring that we test 10 infants a day if our machine can't test 10 a day, what will we do? So maybe it may be necessary to split them looking at sometimes they have no infant on a day, sometimes they have five, and others coming from other facilities. I think it is also necessary that we also accommodate those that are coming from other facilities. Because those whose infants are born at the facility are already booked at birth and one week, but those just coming randomly are the ones that we are worried about, saying what if they exceed the number the POC can handle? So that is why our booking we are putting a smaller number to accommodate what may come.

# Refining strengthened leadership

## Group 4

The total number of women living with HIV identified on a shift and how many infants have been enrolled in HIV care must be included when reporting for proper follow-up. We also agreed on the point of facility mentorship based on the identified gaps by the focal person and the in charge. Still, we added that the facilities should also use CPD sessions to facilitate mentorship of the healthcare workers. We also added that during the handovers, if some healthcare workers are not familiar with the filling of the cards, the nurses should teach each other so that they can document adequately next time. And if there is an HEI during the handover enrolment, prophylaxis should be verified.

There are three areas to strengthen leadership.

The focal person should lead the reporting of the HEI, i.e. knowing how many HEIS were available, getting prophylaxis and enrolling in HCC.

Coordination of PMTCT women in all departments to ensure that all services are provided, like ANC and POSTNATAL. The focal people should know about these and initiate meetings for healthcare workers to follow up on them.

Page 66: appointment of the nurse midwife who will work hand in hand with the nurse in charge as a focal person to lead the team of nurses in PMTCT services and comply with the guidelines. We have not changed anything.

Develop task allocation: we have agreed on all these.

Appoint an equipped focal person (what kind of interest)

So, we added the interest that the focal person must have an understanding of PMTCT requirements from ANC to Postnatal

The follow-up bulletins were not very clear. It appeared as if the focal person was already there and at the same time as if we were appointing the focal person, so I think there is a need for clarification. Because there was also a mention of the qualities of the focal person

There was also a mention that some women just came to give birth. The focal person needs to support the nurse to have these women's infants enrolled in HIV Care and transferred out.

PMTCT focal person is not replacing EID focal person. Are we able to see the difference there?

So, we separated the duties of the EID focal person as follows

Work within charge, and all nurses

Ensure identification of EID clients at the postnatal clinic and ensure that they are enrolled in EID. We also felt this might be cross-cutting even for the PMTCT focal person.

To ensure infants are enrolled for day one and one week, not always at six weeks.

EID should ensure that pink cares are correctly filled and also track the milestones for the HEI if enrolled and offered HIV testing

Ensure mentorship for EID services

Now the PMTCT,

Nvp supply is to be given at first contact no matter how the pregnancy is

Timely reporting

Guidelines

So, we are looking at the focal person as aware of the PMTCT guidelines and passionate about ably tracking the necessary services to be offered to PMTCT women.

The focal person must work hand in hand with the nurse in charge. We all know that the nurse in charge is an overall person who oversees whether all services are offered accordingly. Things will not work if the nurse in charge isn't interested or leaves everything to the nurse.

Individual

So which duties are for whom, and what's the differentiation?

Individual

Cross-cutting duties for EID and PMTCT, focal person duties, are as follows:

1. Work with the nurse in charge
2. Entire identification of infants

## Researcher

I think for me; it is the difference between the two? What is the difference?

## Individual

Were you able to capture the end of the focal duties? That there should be ensuring that the pink cards are filed accordingly?

Individual

Yes, even that they should be enforcing that the HEI are tested according to the milestones

Individual

So, should we remove the cross-cutting duties?

Individual

Not really, but it is important to be clear what are the roles of each who is doing what.

Individual

So, about the PMTCT, have you captured about ensuring testing of women at ANC, supplying of prophylaxis

Individual

Yes, I have captured all that.

Individual

The way I look at this, the PMTCT mainly focuses on supporting EID services at the maternity, working with the in charge to ensure all is well and delivered.

The EID focal supports and coordinates the services now that the infant is six weeks and has been tested.

Individual

So, will the EID focal person not coordinate and support services at birth, like enrolment?

Individual

She will, but the person at the forefront is the PMTCT focal person.

Individual

Why are we demarcating the PMTCT and EID focal?

Individual

I think it is good to note the background. But remember, there is a point that we are also saying that a PMTCT focal person is not replacing the EID focal person.

So, we wanted to differentiate who the EID and PMTCT focal persons are, so we are also isolating their roles.

Individual

If one person can do all these, is it not possible?

Team, it is possible.

Individual

I want to explain what we do at our facility. We have an EID focal person

Individual

Before you continue, what profession is your EID focal person

Individual

HSA, even according to the roles and responsibilities it is suiting, and with our work, we work hand in hand with the HAS. The EID focal person is a HSA, and they are the ones that champion looking at the pink cards, checking that filling is done accurately and also supporting the reporting. So, if the EID focal person is a nurse, things will not work well. And to conclude, the PMTCT focal person has to be a nurse or midwife because they will be present in the labour ward, where enrolment occurs and can spot most problems and provide support. In comparison, the PMTCT focal person could be anyone.

## Researcher1

From our experiences, there are other facilities where nurses accept that they cannot enrol an HIV-exposed infant because they have a lot of work, for example, from what Facility F mentioned.

## Individual

Yes, the nurses say they are busy.

## Researcher

And if we take that facility, EID focal person is also an HSA. Now to ensure that infants are enrolled, you see that the HSA works hard to enrol infants when they come at six weeks because he cannot work at the labour ward and does not know if an infant requires enrolment at birth.

Individual

At Urban, we had an EID focal person, but the program was performing poorly than it is now. And we thought we could have a nurse.

## Researcher1

Okay, I see that this is now context-specific. If we have EID focal person as a nurses' things work, while in other facilities where we have EID focal person as a HSA, it does not work.

So, the question is, in facilities where we only have EID focal person as an HSA That looks at both at birth and six weeks, how is the enrolment?

## Individual,

So, if registration is not done at the maternity, the registration is poor, unlike if the registration is done at the maternity.

## Researcher

I want to recommend both of these facilities because, after the initial finding from the study, they have improved many things. I will give you an example of Urban. They now have polythene, where they put cards for those enrolled at birth.

Individual

We still have gaps the delays, and when the infant is sent to the lab will no longer meet the nurses now, there are different healthcare workers.

Individual

For example, a lab person or HAS

## Researcher1

Do we expect the work one did at births regarding NVP administration? Do we hope that the HSA, now at six weeks, should ask the mother how it happened and then document it?

So, the rural facility has shown strong coordination in line with the EID services required at different milestones and the professional providing such services. So, the PMTCT focal person should be the nurse, knowing that the nurse fully understands the consequences of missing a woman in the labour ward and the nurses she is working with. And if there are any challenges, let the two coordinate.

Individual

Individual

That is why we, as managers, suggested separating the roles and having some still cross-cutting.

Individual

What did you discuss as the differences?

Individual

What are the challenges we are identifying if we have a nurse as a focal, and what are the challenges we are identifying if we have a HSA as a focal? And others reported different findings in the various facilities where other nurses would leave things and not do because they do not know. And they would say I have stopped working on this.

Individual

## Who says that? The focal? How, then, did she qualify to be the focal person?

Individual

## That is why we are saying that we need to select someone equipped.

So, for a focal person looking at things more seriously in the labour ward, it must be a nurse and work hand in hand with the EID focal person at six weeks.

Individual

I have now started understanding these things and can separate the qualities and the roles where we say that the PMTCT must be a nurse, not so. We are changing and confessing what we have not done well. I also overheard someone there saying.

Individual

Before we have someone summarising these things, we need to know that PMTCT and EID are one program. So, EID is a continuation of PMTCT, so we must consider this when working. Now there was a specific response saying other facilities are working well where there is a HSA as a focal person. If that is working, let us leave that like that. While if things are not working well, then a trained nurse should lead as the PMTCT focal. So, in the beginning, the nurse can help well, while when we reach 12 months, the HSA or HDA can perform better as a focal person. So let us know that we do not necessarily have to change where things work well. Let us leave the same HSA focal person; we can only change where things are not working well.

Individual

I need to agree with what she has just mentioned. Like at Facility Z, things do not work well if we leave this to the nurse or clinician, but if we leave it up to the HDA, things go well.

## Researcher

What do you mean everything goes well?

Individual

What we mean is that when a woman comes with an infant at six weeks, they will ably test the infant and document and also enrol in HCC, while if we leave the register at the maternity, the register is not documented at all. Even that pink card is wrongly filled. And because she is also a supervisor, it means she knows.

Individual

And I would like to add. When we say that things are okay, we mean that the outcome is good, starting from the ANC up to birth up to testing and if there is a HSA who is a focal person supporting up to testing at 24 months.

## Researcher

I think the other thing we need to remind ourselves of is why we are discussing this issue. We can't refute that there is an improvement in HIV testing at six weeks. Still, if you remember, while we were sharing the results, even if I share the current data, you will note that enrolment at birth is a challenge, and if HEI is enrolled in HIV care majority are enrolled at six weeks, and few at one week. If we evaluate HSA, they are good, and they are active. Now the challenge is, as much as we are noting an improvement in HIV testing at six weeks, how do we handle the challenge of enrolment at birth? Should we say that the HSA is equipped enough to lead and support to ensure all HEI are enrolled in HCC? We do not want to spoil the leadership no. But where they only have a HSA as a focal person, should we leave, that enrolment should occur at six weeks? I will give you an example of Urban, which has greatly improved. Initially, you would be challenged even to have access to the registers….

And with the current plan to improve handovers, we should also report if we have enrolled an infant or been given prophylaxis. Do we aim to say we should invite the HSA to be there at the labour ward to support these initiatives? We do not want to destroy leadership, but we acknowledge that they are never at the labour ward as much as they are there, supporting that testing at six weeks should be possible. And whether you like it or not, at six weeks, HS A can't test an infant without enrolling in HIV care, and they will still enrol the infant and when we are looking at our enrolment will see if enrolment is good, but that has effects on prophylaxis uptake sometimes. So, as nurses, will we accept that even if we miss these women, the focal HSA will capture the HEI at six weeks? Do we care about the enrolment milestones and the prophylaxis? I believe the background is put forward. I will give you an example of F. We piloted the study and had 100% of infants enrolled at six weeks and 0% at birth.

Individual

Who is a PMTCT and EID focal point? You have explained well, but sometimes you find another nurse who does not know these things and the HAS who is very conversant. It is not that we are disrupting the system. The point is to find a person who will be helpful.

Individual

We also need to know that the PMTCT focal person is not coming in to take overpower. But this is suggested because of the gap in maternity to sort out those problems. Because even at the ANC, the HSA does not include the labour ward. And things have been going wrong even if outcomes improve at six weeks because the enrolment is done at testing. So, this PMTCT is coming to support the nurses who have difficulties understanding and following up on HEI. To enhance fellow nurses

Individual

I remember discussing this issue with the people there during the formative study. We agreed that there must be a PMTCT focal person to support nurses and midwives in the labour ward and postnatal, and now, at six weeks, we can have either a nurse or HAS to take over from six weeks, and these two can coordinate and support each other.

# Refining data validation

## Group 5

Our corrections are in selected places like slide 74, where nurses must give each other handovers and review documents. And so, we proposed the required three documents to be at the maternity. HCC register, pink cards, and we said these need to be at the maternity

We also added that if there are students and new staff, we need to orient them, and we thought in our report book, we need to include the need to add the variable for PMTCT women.

We just collected on what we are referring to the cards … we should be calling them pink cards and not under 24 months to avoid confusing healthcare workers.

On a point for verifying if booked MIP have reported for testing, we added that the nurses allocated to the postnatal ward or labour ward should do the verification. And we thought that if the postnatal nurse has missed, the family planning nurse should help to verify and equally the HSA from the under-five

Place a folder and blank pink cards. These need to be at a place that is accessible and also orient the students and all health care workers.

Excel sheet … we said this is a good idea but may require every nurse's orientation. But we must be vigilant enough.

Individual

Of course, I considered using an Excel sheet on What's Up, which will be available to all nurses, right? I think I was not able to understand this one. It appeared as if it would be entered somewhere, and I felt that the responsibility of leaving the data entry to every nurse might be a challenge.

Individual

Of course, it is good to still have all the nurses oriented so that they know why they are asked for that data.

Individual

I have one question for people that we are in here. Is it possible to have the HCC registers in the labour ward?

Individual

For urban, I think it will be possible may be Rural.

Individual

Rural, will it be possible?

Individual

We can only do the pink cards, but the HCC will be at the ART because we had challenges having it at the labour ward. When we enrol an HEI, the nurse walks to the ART to enrol, gives an HCC number, and pairs the infant with the mother, and we get help from HAD and HSA.

Individual

Weekend how do you do it?

Individual

We give each other hand over and sometimes retake it from the ART to document. The weekend we write pink cards, and the infants are enrolled in HCC on Monday.

Individual

I would like to understand what the gap was.

Individual

1. nurses have a different attitude. Other nurses could write while others could note, and infants ended up being enrolled at six weeks with anyone offering services at six weeks. If the focal person was not around, then the numbering was not done in order, and there were a lot of challenges. So, we did a QI project, and as a team, the change idea was to change the location where the register was placed. It is when we agreed. And if we are to change this may bring problems

Individual

So now that you are saying that the register is at ART and a nurse enrols the infant and walks to document, is it, not the same that each nurse can write the way they understand and even not walk to go and enrol the infant?

Individual

For urban, is this arrangement okay with you? Because what we will agree on is what we will take as the decision and train the rest of the healthcare workers.

Individual

I have still noticed that the rural facility has a challenge. As they said, the register stays at the ART during the weekend, and they open pink cards and documents on Monday. I am unsure what challenges they face because missing infants is possible without enrolling them, and also the mix-up of numbers mainly since the cards are compiled, and enrolment is done later. So, I am not sure.

Individual

So, we do handovers. And we have no challenges because we document everything on the card.

Individual

Does this mean that the infant collects the number at the next visit?

Individual

We do not document the HCC number in the book of the infant.

Individual

I want to give an example of a situation at the Urban where maybe another woman gave birth at a different facility and came to the Urban at six weeks. People at the lab come to the maternity to register the infant.

Individual

Our aim of putting the HCC register in the labour ward was not to miss a lot of HEI, which we were when the register was at the ART.

Individual

We need to discuss

How to handle the differences?

Individual

Suppose the rural facility can't have the register at maternity and see that they can enrol HIV-exposed infants at birth. I don't know the problem the register to be at the maternity. Let me give an example of our facility. We used to have the same problem. Others would register the infant, whilst others will not. And if we transfer the register to ART, there would be confusion because of that difference. But if we all catch up and register all infants and the nurses can now carry the cards to ART, meaning they know about enrolment, then I do not see a problem with having the register at the maternity.

Individual

The other reason we are having the register at the ART is that we have others that we refer, and they go straight to the ART, including the transfer-ins. What about those that go straight to the ART?

Individual

But if you compare infants that are coming as transfer and those at birth, you will see that the majority are the ones at birth, and those that are just coming would still be quickly enrolled from maternity.

Individual

Researcher, can you please conclude how we will sort out these?

## Researcher1

I am not supposed to conclude because you are the owners.

I want to add this. How does a client move when they come to access health services? I added a flow chart which we observed in this facility. Suppose a woman is coming for a postnatal check-up at six weeks. I noticed at the urban that most women could start at the postnatal, and maybe very few started with the under-five. It means that the first encounter is postnatal. While from what we are hearing from the rural, the first encounter is the ART. But we also know that these women will require six weeks of check-ups. So, it may be good to ask ourselves which one may be the best entry point, which has to be agreed upon by all the HCWs. But at the end of the day, we can't force the facility. It will take up to what will be agreed, and that has to be known to everyone because we may end up having something that does not work.

Individual

Hmmm, let's leave the things that are working.

## Researcher 1

What happens in the rural at six weeks? Where does the woman go? Do you have the pink cards at six weeks at the maternity or postnatal? Or do you have the HCC?

Individual

So, the women start coming for postnatal, and we refer them to ART after assessment.

## Researcher

Do they go without a card?

Individual

When a child is born, a nurse opens a card and brings the card to ART the following day or the same day. And we pair the card together with the mother's file. When the infant is six weeks, they go for a postnatal check-up. After check-ups, they go to HTS. And the expert client looks for the mother's file and infant, and after that, the nurse comes again to the ART to complete CPT, and then the child goes.

## Researcher

Considering their context, maybe they can use the pink card for verification because that is the document they have

Individual

Maybe for this study, we can transfer the card at six weeks.

Individual

I am not working at the urban, but I want to mention something

. The HCC used to be at ART. But we tried moving the register to maternity to open the pink card and HCC because enrolment is not only the pink card. Upon enrolment, the pink cards remained there for six weeks when the infant came. And for the nurse doing postnatal offers the pink card to go for testing at the lab, and after that, they go to ART to receive CPT and other services, and what happens to the transfer in people from ART comes to maternity?

## Researcher

I think they can validate using the pink cards but during training this can be discussed with entire facility being clear on care pathways

# NGO EID experiences

Individual

We do not do ANC but provide HIV TESTING, ART, and CERVICAL cancer screening. So, for ANC women who come to access ART, we offer intensive education on caring for the pregnancy and the baby. Because we do not do ANC, we are challenged that we do not have prophylaxis for these women. When these women have given birth, some come with pink cards at six weeks. Others do not, and we keep caring for them until the infants are discharged at 24 months. The challenges that we have met are that some health facilities ask these women to stop accessing ART from us because we do not give prophylaxis and register where they are attending ANC.

And the other challenge is that those women that come to us as referrals come with pink cards that do not have a unique ID.

Individual

That is true because we received pink cards without barcodes while ago. So, it was the national system error, not necessarily a district. So, we were told that they would distribute pink cards, which would replace the existing pink cards the entire Malawi.

Of course, we appreciate what you have shared with us, mainly focusing on patient-direct care. But we also know that you support other activities for the EID program. Like you support the technical working group and are the only partner who has consistently reported at the DHO on the project they are working on and supporting the technical working groups.

## Researcher

More questions will come when discussing issues of sustainability. We appreciate that they support the TWGs.

Individual

We mainly work in two health facilities, B and M, and we follow pregnant women until the birth of the infants. We followed 1666. When we remove those that have transferred out about 70, we remain with around 1500 something and no longer follow new women. We want to finish the ones we have been following to check if they will be tested at 6, 12, and 24 months. We have also initiated male involvement for pregnant women, whether inviting them to come with the woman at the next appointment or talking to the husbands to ensure they understand the requirement to prevent HIV transmission to the child. We identify these women using a stamp, which we put a sticker with an ID of MPTCT because we do not follow all the women accessing care at the facility, so we use that stamp to identify those we are following.

Individual

That is what NGOs can do. But I also would like to add that their project is almost ending. They are no longer enrolling new women, and they are only follow-ups. Initially, they were also able to support the district TWGs, but since the project came to an end, they have also stopped supporting the TWGs

## Researcher

I think there is a point I missed you were following women from ANC.

## Individual

So, they could enrol a woman in their project, and if the woman gave birth automatically, that infant was also enrolled in the project. They could also follow the infant for up to 24 months.

## Researcher1

So, I want to know if the project was at the facility. Do you have your criteria?

Individual

We did not disturb how services are offered because we looked at sustainability issues. What we did was to train the healthcare workers for them to know. Now, what used to happen when a woman comes for ANC? For those who were HIV positive or already on ART, we could enrol them in the program, and we could work with them to ensure that they come to the facility for all four visits. We aimed to ensure good adherence and then see how best to support the baby. So, they were going through routine care, and we could only come in if there were gaps. For example, if they tell us they did not receive prophylaxis, we could invite them to the facility.

## Researcher

Okay

Individual

In addition, after they oriented the volunteers, we were also oriented as district coordinators and were aware of it. It also showed a significant impact. It was good, but we would like to know when and at which sites this will be rolled out.

Individual

For now, we would like to complete and learn the outcomes from this then we can consider rolling it out to other facilities. However, this is also dependent on the availability of funds. Because we have both lessons

Individual

Good morning again. We do research, and we do not follow a programmatic approach. We are studying neonatal transmission and actively working with the EID program because we need to confirm HIV transmission and enrol the baby.

So, the part of EID we focus on is the treatment side. Of course, we also have other PMTCT studies. So, the studies focus on cures. We identify infants that have HIV during the first three days of life, and we try to find out and confirm if that transmission occurred while the woman was pregnant. We initiate the baby on ART, which is aggressive, to see if we can functionally cure the baby as a functioning study because this has not been proven before. The concept exists if one gets the transmission, and the person is initiated to ART immediately. Maybe after two or three years, we will interrupt the ART and see if the baby can naturally control the HIV. And that is what we call a functional cure. And the babies we identify are those at a high risk of infection and those we identify during pregnancy or even breastfeeding if we identify them early. So, we aim to save the children from taking the medication up to 60. So, you are working on identifying the infant early, and we are focusing on what to do when we identify the infant early, something different from what is happening now. So, we are evaluating if we can cure the baby if we identify them early. And we support the DHO with testing. That is why earlier, I asked what platform you are using to test. What we have in our lab is the gene expert. Sometimes, we are asked to support the testing because we can run four samples simultaneously with the expert. And occasionally, we are asked to help if cartilage is out of stock.

Individual

Any comments

Individual

Of course, what they have said is very accurate. Sometimes, we have so many samples that we need to process even during lunchtime, and they support us with lunch allowances, and we work hand in hand.

Individual

I want to ask how many children are enrolled in the study.

Individual

In one study, we enrolled about 23 over a couple of years because finding infected babies within 48 hours of birth has been challenging. Our criteria are not to identify infants after some weeks. And the fact that we are having difficulty identifying these infants shows us a good thing because it means PMTCT is going well. When we find the babies, it indicates that there is a failure. Or sometimes, this happens when one is tested early, and they are negative, but they are later seroconverted, or the woman is not taking ART. And we enrolled 29 infants. Unfortunately, the results of one study we finished did not show that it was effective compared to the standard of care.

## Researcher

What is the catchment? Is it the entire Blantyre? And how do you identify these women?

Individual

Yes. We do not have permanent staff in these facilities, but when we go around, we sensitise the healthcare workers about all the studies. One nurse goes around weekly to remind them they are doing this, and we get a call maybe once a month.

## Researcher

So, does the nurse go around all the facilities once a week?

Individual

Not all the facilities. But the nurse goes around to around Five facilities.

## Researcher

I am asking because the Blantyre district, in the experiences pointed out, to be challenged with mentorship and supportive supervision, including transport. And knowing that there is a nurse who moves around, maybe we could reflect if they could be joining the nurse at one point.

Individual

That is a good idea. Because we have someone who already goes to these facilities, joining it is a good idea.

## Researcher

I hope program managers can follow that. You will be able to link with NGO4, and we could explore further how to do this when we are exploring sustainability issues.

Individual

On identification, there was also another nurse we called when we had an infant that tested positive, and she could further collect the details.

Individual

To recommend NGO4 and even the DHSS recommends the EID program regarding timeliness and completeness of reporting because of their support. Because they support the program coordinators with airtime to track reports from the facilities

Individual

Yes. I remember we identified that as one of the challenges during the TWG meetings, right? It's a communication challenge.

## Individual

So are yet to develop new interventions, which we will agree on in January.

So, we support the EID program with mentorship, psychosocial support, and default tracing. If there are issues with the woman's willingness to start ART, we will let you know. We will also let you know if we are to create other new interventions.

## Researcher

So, you were doing mentorship and supportive supervision. Did you stop? And if you quit, what were the reasons? And if at all you have managed to evaluate the impact of the mentorship on the EID program

Individual

No, we did not stop. It was just an issue with funding challenges. So, we have two forms of mentorship: facility-based and district-based. We cannot give the entire picture of the district but an example of the urban facility. We can proudly say that we have supported. Checking if the prophylaxis is available and if they are on the prophylaxis

Individual

I would like to agree with and support what they are saying. We cannot talk about EID without NGO1. They have helped us even with quality improvement, and we are doing well because of them.

Individual

We have different support from NGO1 with nurses stationed in facilities and district officers. Of course, they are not in all the facilities. We note that the facilities with NGO1 staff benefit greatly when we compare facilities that do not have. So, mentorship was happening in two folds. As the district, we would go to different facilities, and those NGO1-based staff could also do facility-based mentorship. As already discussed with previous support, we could have mentorship, but now they do not have funding, and it stopped.

## Researcher

I would like to know if we have the NGO1 staff available at both facilities, urban and rural.

Individual

At the rural, we have one that comes, but we do not have a facility based.

Individual

Out of the 52 sites, NGO1 does not support all the facilities. They also have other sites that they do not help. So, please note that we expect you to shop from the list of needs the district has, not only doing what your project is planning. And we still look up to your NGO for support. We do not want the allowances but even providing us with transport would greatly help the district. In addition, we also used to have data review meetings. It assisted each facility in analysing its data and coming up with solutions.

## Researcher

Okay, Now, from what the rural facility has said. The mentorship that was stopped is where you pick the coordinators, but are you still going to the facilities as the partners? If yes, they can go. Is there a way of coordinating to pick these coordinators still because they say they do not need allowances? If what they are saying is the truth, it means the transport is still there.

.
