# Supplementary material for: Developing an intervention to improve early infant HIV diagnosis service uptake among postpartum women in Malawi’s primary healthcare using a co-designing approach with stakeholders
Source: PLOS Glob Public Health. 2025 Apr 22;5(4):e0004426. doi: 10.1371/journal.pgph.0004426 (PMC12013899; doi:10.1371/journal.pgph.0004426)
Supplement: S1 Data — (ZIP) [file pgph.0004426.s008.zip › S1_Data/Sustainability Transcript.docx]

# Sustainability

## Group 1

**We have expressed ourselves according to how we understood the question. We understood if the researcher is currently supporting the implementation of the interventions because it is part of the study regarding resources and other requirements. If she has finalised her study, what could we do to ensure that the intervention continues, and do we see the intervention being sustained?**

The facilities should be able to own the implementation of the interventions. We should take the intervention as our own. How? We need to appoint a focal person both for PMTCT and EID. We also need to form a quality improvement team to ensure that we can still identify clients and not miss providing services to HEI at any point where we must identify the HEI. And if there are any issues, we should ensure that the team should be leading in addressing the identified problems during the implementation. We will also need to source some hardcovers and registers from the MOH, DHO or implementing partners, which we have among us. We have A, B, C, D, etc, even though they are hard to support. But at least when we are challenged, we can still approach them to help us. So, for stationery issues, we can source from DHO and other partners working within the facilities.

We have heard that all the healthcare workers in the facilities where the study is taking place will be trained. Or will be fully equipped with the knowledge and skills to implement the interventions. So, we have staff turnover; those who may be trained now may be moved out of the facility, and we may have new healthcare workers. How are we going to sustain the implementation of the intervention? So, we will have continued orientation for new staff joining us. We will have mentorships and supervision for our activities at the facilities.

We have heard that some of our partners have stopped supporting and conducting supervision and mentorships because of limited funding. But our coordinators have requested the partners to at least assist them with transport only to go to facilities for supervision and mentorship, even without lunch allowances. So, we can take that one on board and use it as an opportunity to continue supervising healthcare workers and mentoring the new staff.

There was also another point on what we perceive as the intervention's benefits. We aim to ensure that we cover 100% of the identifying clients. We do not want to miss any HEI. Sometimes, we miss them, but with this intervention, we want to identify every HEI and track and manage it accordingly.

That is where we stopped. My group members can add.

## Individual

Should we give a platform for others to ask questions, or can we have another group present since we are discussing different issues?

***Silence***

## Individual

Do we have a question or comment?

## Researcher

I loved what group one discussed. They have been honest about some of the points they have owned the intervention. Group 1 has noted that they will be challenged with resources and mentorship, in addition to explaining that they have already started suggesting where and how to source some of the requirements. I also remember that at a certain point, program managers from Blantyre DHO highlighted that they also have needs, and the partners must shop from them. The goodness is that we indeed have partners here. What are some of the inputs from partners here? How do you look at this? And, from this group, they may also need support for the programme managers to visit different facilities. Any inputs?

## Individual

Can you please repeat this?

## Researcher

Group one feels they can sustain the intervention but may need further support for stationery and transport for supervision. I remember one of the partners, when they were expressing their experiences, mentioned that they have a nurse who goes around different facilities each week to collect results for HEI. What about leveraging that one to work as a team so those aiming to supervise could join that nurse?

## Individual

So, what is needed is stationery and what?

## Researcher

They may need transport to go to different facilities.

## Individual

I need to clarify about the mentorship we were referring to here in group one, which staff at the health facility can do independently. We considered that all healthcare workers would be trained for this intervention, and then we thought if we have staff turnover. For example, suppose we have 100 healthcare workers trained at the urban facility. In that case, we will sustain this by training, orienting, and orientating those new staff coming to the urban facility. So that everyone else coming should be oriented just like we are doing with other programs.

## Researcher

So, do you need support with that

## Individual

So, how will we sustain that we will orient new staff and those not doing well? We will continue with mentorship and supervision to ensure they do the right things. So, with the within supervision and mentorship, do we need transport for that?

## Researcher

I do think you will need transport within. But overall, the program coordinators may need to supervise the implementation.

## Individual

Hamm yes yes

Those aiming to supervise could join that nurse, which partners said they have, and she moves around.

## Individual

I think we can support the transport

## Researcher

I think the program officers and coordinator need to follow up on that with the partners, and there is also an issue with stationery

## Researcher

We have taken note of that

(Background noise)

## Group 2

**We had four items to reflect on 1: active identification, booking system, strengthened leadership and daily data validation.**

All these things are okay, and they can be sustained. Even after the study, they can be implemented without any problems, and it is possible. But only tracking might need funding on patient identification and tracking like it is all the time. When we come to the booking system, we may have a problem with the stationery. But we can approach the partners like the other group described. Because sometimes we already have stationery challenges. We feel it is doable to strengthen leadership, but this requires commitment and teamwork in the facilities. Good leadership in our facilities. And on daily data validation think this we can do, but we need commitment and willingness from the staff. But this will be doable guidelines in overall ART. The ART clerks hear about the changes.

However, we think the challenge is the funds we may need to trace the women and stationery, but we are glad that partners will support the stationery.

(Probe on how we can strengthen leadership and commitment)

The commitment of healthcare workers can be achieved if they are well supported—for example, if there is mentorship and availability of resources, for example, reporting forms etc. There is also a need to be inclusiveness of other healthcare workers rather than inviting the focal persons alone to meetings. In leadership, there must be openness, especially when funds are available to support activity at the facility. Meanwhile, when there are funds, they consider certain cadres, and others vanish. There is also a need to appreciate healthcare workers doing well, for example, having a get-together. Lastly, lower cadres like EID clerks are the ones who work a lot in EID, and these are not considered to be oriented when there are new guidelines.

## Researcher

**Could you clarify about tracing?**

## Group 2

You had given us active identification and tracing

## Researcher

**T**eam, may you help clarify again what we agreed on for the first initiative**,**

## Individual

It is tracking, which will be within the facility where we will use specific labels to identify and track participants.

## Researcher

Will we need funds to do this**?**

**Responses from several:** No… it is active identification and tracking

## Group

Tracking will still be part and parcel of this. If we have booked women in the booking system register and they are not coming, we will still need to follow them, and we may collect their details in the booking register. We should be able to follow them; it will be difficult if we do not have funds.

## Individual

I am supporting group 2. We may have defaulters, and it is possible that tracing will be needed

It is true that despite the intervention, we may still find other women who may still decide not to come, and we need to trace them to bring them back into care.

## Group 2

We will still need funding to support the healthcare workers who trace the clients.

## Individual

Let's clap for group 2, and may we have group three?

***(A delay in having group three present)***

## Group 3

We were discussing implementers and population characteristics, looking at our three initiatives.

The first thing that we reflected on is attitude. We know that most healthcare workers have negative attitudes. Those with negative attitudes toward them should change or implement some of the initiatives we need to pair with other healthcare workers in a good mood to motivate them. In addition, for the people doing well in implementing the initiatives, as the facilities, we need to acknowledge them and appraise them. Not necessarily by giving them something but even verbally. For example. So you are doing well, and the person will feel good and likely continue the implementation.

I think all the groups that have presented previously have also mentioned supervision. It helps to ensure that gaps in implementation are corrected and that if there were other areas that were well done, the healthcare workers should continue doing well.

In addition, on issues of meetings, there should be variations on who attends the meetings. So, for example, not having one person attend meetings every time, like one member attending an appointment today and again attending the meeting tomorrow, while other healthcare workers are available at the facility. So, if there are rotations, healthcare workers will be motivated knowing that they are equally considered when it comes to meetings and may have a positive attitude that they should also work hard to implement the interventions.

In addition, there is a need for on and off onsite mentorship and supervision. It is possible for the healthcare workers at the facility, including those in charge, to arrange with other Healthcare workers within the facility to conduct mentorship. The in-charge can find healthcare workers she feels are performing well on the initiatives to mentor other healthcare workers within the facility. In addition, we also have people from the district. Still, we can start at the facility to support each other, and the rest can come after we have already begun mentoring and supporting each other.

Intensifying health talks in the community, focusing on the population we are serving. It is essential for the people we are working so hard for to know what is required of them. You can agree that sometimes some women do not understand the need and milestones for monitoring their child. You would note that other women have opted to stop bringing the child to the hospital independently, not that the healthcare workers have advised the woman. So, if we intensify the talks, it will also help them understand the need. Thank you very much.

## Individual

Do we have questions or comments before we give people from the referral facility to present?

## Researcher

**I am looking at how practically you would identify healthcare workers with attitude to support them.**

## Individual

There is a question on how to identify healthcare workers with attitudes to pair them with someone doing well.

## Group 3

Those who don't mind identifying women who are HIV positive or initiate the HEI to be tested, some of them talk about it in the open that they don't associate themselves with being responsible for such roles. Some openly say others verbalise that they do not have adequate knowledge of some procedures.

Even those in charge know the people they are working with. Those who do not want to work

## Individual

## Even documentation, you could see how they write notes for the clients

## Individual

We know each other. You know that if there is such a person on a shift, all the infants that are born from a woman who is living with HIV will not be enrolled in an HIV care clinic. Even when you ask them, they will express that they were busy, or they may document but with a lot of incomplete data

## Individual

That's very true. Currently, someone in charge calls the staff who are not doing well and discusses some of the challenges they are failing to address so they are aware of them.

## Individual

Do we have any other comments or questions? If we do not have let's proceed to the next

Presentation

# Experience with EID services at Referral

## Individual

I will give you an overview of the PMTCT program at the referral facility. We have 13 sites: antenatal, family planning, the whole charkha—that's labour ward, postnatal, gynae, hmmm, chatikha nursey, paediatric, admission, and A and E. So, these are the sites where the primary health facilities you refer patients to us are not. So, they?

**Team:** Yes (background noise)

## Researcher

Some are saying that they did not get what you said

## Individual

We said we have 13 sites: postnatal ward, antenatal clinic, A and E, antenatal ward, labour ward, family planning, NRU, paediatric, special care ward, paediatric nursery, Mercy James, and 1A. So, we have obstetrics, gynaecology, and paediatrics, the areas you usually refer us to.

**So, we were also discussing the importance of testing women when they have come to give birth so that we know their HIV status not so?** So that is what we have discussed a lot. We are currently answering a case of the referral hospital, primary health facilities, and district health office. Because the problem happened at the referral hospital, you will realise that the issue involves all of us. So, for this woman, the woman was attending antenatal at W Health Centre, and just for you to note, we are still following this issue. The woman completed her antenatal care at the W health centre. However, it was showing in her health profile book that they had not checked her HIV status until the woman gave birth right at the health centre.

In the maternity register, similarly, there was no documentation of HIV status. The woman was referred to the referral hospital. The primary health facility documented that they referred the woman due to post-partum haemorrhage. Then, the woman was cared for and was discharged when she was well, but note that even here at the referral hospital, we did not check her HIV status even though she had babies as well. Three months later, she returned with one of the babies she had given birth to and was seriously sick. The woman had given birth to twins. Since she came with an ill child again here at the referral facility, the woman was then tested for HIV and was found HIV positive. However, the woman denied the results that they were not hers because she indicated that she had an HIV test wherever she was accessing services while pregnant and was okay. However, when we explored the documents, we noticed that the woman was not offered an HIV test. While we were processing all this, the baby died in the paediatric ward, but the woman told the hospital that the death was their fault, and she was not accepting that the baby had died, and the hospital should take responsibility. The other baby was also HIV positive, and she mentioned that she would bring the second baby to the hospital to start ART after burial. But she, later, did not get the baby here. You can see that we are having problems. And most likely, we may also lose this baby. And if that baby is not found to be put on antiretroviral therapy at any time, she can die. And if you think of that woman, it also means that there is a possibility that she may continue to transmit the infection because she also denied being on treatment, but we are following up. So, we are all to blame. HIV testing services are very important at any point. In the latent phase, we think that healthcare workers at facility W could have tested the woman without waiting for active labour and tested the woman after delivery. Even here, when the woman came to give birth when she was referred were also supposed to test her.

# EID services

## Individual

**So, I will share with you how we currently provide services.** What is done here is we have partners, i.e. G, who have HIV diagnostic assistance that assists us. Initially, we had Q, but now we have G. The Q only supports us with health promoters previously referred to as Expert clients. So, our maternity register is placed in the labour ward and the HIV care clinic registers at the postnatal. Sometimes, the register is also placed at the antenatal only during the weekend. That's when we leave it at the postnatal because it is open 24 hours and accessibility is easy. The one who documents in the maternity register after the woman has given birth is a nurse midwife. But the HIV testing services room is within the labour ward. At any point in all the sites I have mentioned, they all have HIV testing services. This means that at any point when the woman is in the latent phase of labour, the woman is offered HIV testing. On the aspect of documentation of the HIV status, it is always documented in the passport books, and if the nurse has not updated, even the HDAs can update. They review the column for the HIV status in the maternity registers, and they update it. Suppose the HDAs are not trained that the status is also documented in the maternity register. There is a gap in that case because they need to know that it is also part of their work to see that the tested woman has an HIV status documented in the maternity register.

# Challenges with EID

## Individual

However, we still have challenges, especially during weekends. Sometimes, we have a low turnout of HDAs who provide HIV testing services because it is a weekend, and the nurse is not trained. You Would still find some gaps in the maternity registers. There are few not done, which is because they may have given birth during weekends, and some beg that they want to be discharged before the working day and are discharged, and they go without an HIV test. Remember, we also respect patients' rights. Sometimes, there are issues of documentation that the HDA has documented in the HTS register, but the result was not transferred to the maternity register. We are sharing all these to ensure we are not missing any women. In the latent phase, we must understand that we must offer HIV Tests. That way, it will help to ensure that if a woman requires a referral, they should already have a recent HIV test. That will help the referral facility have a few women requiring HIV tests. On the same note, you also know what the referral facility is like, especially at the labour ward, (Mwana agwiritse) there is a lot of work.

The other thing I would like to share is that if the woman is in the active phase, even the guidelines indicate that we should defer testing and offer after the woman has given birth.

Now I will talk about HCC, HIV CARE CLINIC. Most people are confused about documenting in HCC, but it is straightforward. As we said, the register is supposed to be placed in the maternity at the postnatal ward. So that every infant that is born and exposed should be enrolled in HIV care, we can enrol HEI. However, there are still some comments we have heard even here that you still see women that you referred to are not enrolled in HIV care. Sometimes, we have managed to initiate the mother on ART but have done nothing to the infant. That is very possible. Usually weekends, we do not have health promoters supported by Q, who typically support enrolling the HEI in HCC. Our midwives are there, but we have attitude issues. I am not very sure why we nurses are like that. I am not sure how we were groomed. Others know correctly, but at the back of their minds, they are money-oriented because they were not officially trained and did not receive allowances feeling it's not their responsibility. They do not document and do not provide or dispense the nevirapine. However, I like to mention that we are not sure who our children will marry. They may get married to these same kids we are failing to care for. Our children do not mention what they are doing behind us; tomorrow, it may still be our responsibility.

The team: agreeing

So we need to change our attitude. In your presentation of experiences, you further indicate that sometimes these women do not receive adequate prevention information and are sometimes given inadequate prophylaxis. And because of the insufficient information, we find the women explaining to us when they are referred that they have been taking their antiretroviral therapy together with the baby's nevirapine" They do explain that I have finished all the drugs, both nevirapine and ARVs, while they are still pregnant. Since we already give them in advance the prophylaxis. We all need to own these problems because they reflect our district, whether at the referral or the primary facility. These are our problems. Even when our performance is being shared, whether a referral or primary facility, they refer to all of us as one and show that we are all failing.

On transfer-ins. even if the women had given birth here, we have noted that you can test the infants when they come back to your facilities for the facilities using DBS. You can try them, but do not follow what the register is saying. If 12 weeks have elapsed without receiving results, we must repeat testing the infant and will keep providing other services to the infant for up to six months. We need to remember that we call this early infant diagnosis, right? For the facilities with the point of care machine, it is good to test at the proper milestone, and it's easy because we will know the resulting test at the right time. We wanted to discuss these things so that we need to share the information with our colleagues when we return because we work as one.

Have I kept my time?

No

Okay, but I wanted to share this: We work as one. I have also seen and recognised the different partners. It is good that we work as one.

## Individual

Thank you a lot. Can we give them a hand?

Do we have any comments on referral presenters?

## Researcher

I have one question: you also mentioned that you can test for HIV in women before they deliver.

## Individual

HIV status yes

## Individual

The guidelines recommend that when the woman is admitted to the labour ward should be tested for HIV; however, most healthcare workers we tested at the postnatal. But it is essential that we need to be testing because it does not restrict that you should not test before delivery.

SO lastly is feedback and a way forward.

## Individual

It does not matter at what point we test women when they have come to give birth, whether before or after giving birth, but we need the ascertain their HIV status before they leave the hospital.

However, our fault is that we wait to test them after birth

## Individual

We can arrange with HDAs to ensure that we test before delivery if they are in the latent phase;
