# Supplementary material for: Developing an intervention to improve early infant HIV diagnosis service uptake among postpartum women in Malawi’s primary healthcare using a co-designing approach with stakeholders
Source: PLOS Glob Public Health. 2025 Apr 22;5(4):e0004426. doi: 10.1371/journal.pgph.0004426 (PMC12013899; doi:10.1371/journal.pgph.0004426)
Supplement: S1 Data — (ZIP) [file pgph.0004426.s008.zip › S1_Data/sythesising problems transcript.docx]

**Synthesising the problems**

# Experiences in the identification of clients

## Group 1

This is group one. We first discussed where we can identify clients within the facility. We identify our antenatal care clients as pregnant women starting their antenatal care. We are also identifying other women at the labour ward and the OPD for those who are not well and are consulting the doctor, and these are also referred for HIV testing and counselling. In addition, before we discharged those women, I mentioned them in the labour ward. We referred them for HIV testing. For women accessing family planning, we can also identify those that are HIV positive in addition to the under-five, where infants are weighed. Testing the infants before receiving other services is also recommended for those requiring nutrition services. We identify other clients who just come to access HIV testing out of their wish at the voluntary counselling testing room and during the village clinics.

And in terms of identifying these women, we gauged that we may be at 90%, but maybe we have even underrated ourselves. Still, we think identifying HIV-positive people, we are doing well and only having challenges in other areas.

Regarding successes, we identify these clients and refer them to get tested from most entry points. At the ANC, we know that it is a rule that each pregnant woman must have an HIV test. In addition, women are also required to be tested after giving birth. For each child seeking nutrition services, we ensure that the child is offered HIV testing.

Should I continue?

**Team: Yes**

# Challenges in EID services

## Group 1

We further discussed the challenges that we are meeting. Sometimes, the facilities receive students, and supervising them is difficult and inadequate, leading to some clients being missed. In addition, the information that we give to the women or guardians of infants exposed to HIV for them to have the infants tested sometimes the information that healthcare workers provide is inadequate, and that compromises how the mother complies with the requirements to have the infants receive HIV testing.

Sometimes coordination among healthcare workers is. For example, there are challenges when referring women to a tertiary facility. For instance, after the woman has given birth, healthcare workers at that tertiary facility should create an HIV-exposed card (pink card) and document the services provided. Still, usually, this does not happen when a woman is referred. At this referral hospital, we have seen that their focus is just having the woman giving birth and establishing enrolment of the infant in the HIV care is left for the primary facilities to handle.

The other challenge is if the parent refuses… ahh If the client has been tested. If the outcome is positive, it is sometimes difficult for some to accept starting medication, usually if the parent refuses to begin medication for herself. It also becomes difficult for the parent to accept that the child is an exposed infant, and it is hard to enrol the infant in HIV care.

Sometimes, in these facilities, people leave their roles to provide services to these clients, thinking that the focal person will do the roles alone, which shows that there is no teamwork. Sometimes, parents of these infants give incorrect identifying information, including the address where to find them. For example, people from facilities F and k will provide you with an address from Namoyo. Very disjointed, and sometimes during our review, if these women did not come to the facility, it became challenging to follow up, including at 12 and 24 months.

**Chair**: Are there any additions from the group?

## Individual

we mentioned the literacy level from group one, and sometimes, for these women to follow instructions, it becomes difficult because of their level of education and sometimes the woman's nature and ability to do things.

**Chair:** is that all?

**Chair**: I want to find out how we should approach this. Should we answer the questions now, or can other groups come back to this later after other groups have presented so we can ask the questions together?

**Team**: let's keep and ask together because there may be similar issues that the other groups may clarify

**The chair**: it is now open to the group not for questions but additions.

**Team member**: the comments will also reflect what other groups have discussed as well.

**Chair:** so, we should also not share comments, right?

**Team:** no

## Group 2

## Individual

Have we already appreciated them? Let us clap for them.

Member yes, we already did

**Chair: it** does not overdose

Before the next group, what group did we have?

**Team**: Group 1,

**Chair**: and what's the next group? Group 2

## Individual

Before that, let me recognise the district nursing officer in our midst and ask her to give us the opening speech. Over to you, our district nursing officer.

## Individual

Good morning, all. I will not speak a lot. Let me thank researcher for organising this meeting to discuss our work, and I thank you all for accepting the invitations and showing that we are interested. And this is also shown in the group work. I can see that we are honest in our discussion. For example, one group they were able to indicate that they feel they are not working as a team, and it is very important to reflect on solutions. I do not want to take much time. I realise that our program is quite long. I wish you all a good training.

## Individual

we thank the matron for encouraging us. The facility must understand that the work is not just for the focal, and the focal must know that this is not a bedroom for them and that there is a need to work as a team.

I will not speak a lot. Let us move forward. And let me invite Group 2

**Chair**: Okay

## Group 2

you will bear with us because we have done everything. We were not told that we needed to focus on one aspect.

**Team:** that's great

Team: informal charts

# Challenges

Group Two: we have discussed everything. On enrolment of infants exposed to HIV, we feel this is happening at birth, six weeks, 12 months, up to 24 months,

Of course, the challenges we noted are that some healthcare workers are not independent when working. They depend on others because they lack knowledge on enrolling an HIV-exposed infant, compromising the infant's enrolment. In addition, some healthcare workers can enrol the infant at birth, and when the infant comes at six weeks, the healthcare worker providing services does not ask the mother if the infant was already enrolled in the HIV care program. They start to enrol the infant again by filling in the pink card, which sometimes comes as a duplication. Sometimes, the infant has one to three cards—inadequate knowledge of the mother on what is required. Sometimes, healthcare workers cannot provide the necessary information to guide the women on what to do. Instead of health care workers educating the woman that she should come again at six weeks for HIV testing and the following milestones, the woman is just discharged without information.

Incomplete documentation most pink cards and registers have incomplete data because healthcare workers cannot document, and that's equally a challenge. The next is on students when they come to the health facilities. As healthcare workers, we cannot explain how to provide services, including early infant diagnosis, and this leads to the students having to provide services in the way they like or know according to what they learned. As a result, we miss other children.

On testing, we are doing well. We can take the samples at six weeks and send and even at 12 months. We can collect samples and send and equally at 24 months. The challenges that we found are. We cannot pair the mother and infant on the appointment dates. For example, we can give the mother to come on the 25^th^ while the infant is asked to go on the 23^rd^ or even a month later. Another issue is that results usually arrive late, especially at six weeks. We have a POC machine in Blantyre as a district, which is only available in four facilities when we have several (28). So, for the facilities without the POC, the results come late when we send the samples for processing, and the turnaround time is long. For the infant to receive results after two weeks, the results come after two months or three and compromise the care we give.

There is also an issue of waiting time. The room where HIV testing services are offered is usually crowded, and there is usually a long line of clients because we have several clients from different departments. At times, a mother with an infant wait in line. If we offered services for the mother and infant together, we would not have this challenge.

We also have a documentation issue. Sometimes, these infants are well tested, but there are challenges to having the information transferred to the pink card. Sometimes, if we have supervisors, they find that many pink cards do not have the documentation of results at all milestones, six weeks, 12 months and 24 months.

On the issue of new guidelines, we know that nevirapine is administered to children who are at low risk, and 2p is given out to children who are at high risk. This is very true, and these are in the guidelines. However, the challenge is that just a few people were trained, and because of this, most healthcare workers cannot provide the services accurately according to the guidelines. In addition, if I have gone for training, even if I inform my friends, they will say he went to eat the money alone 45 000pin and that person it means won't be involved in the implementation. However, even those who were trained, some were trained but unable to implement, and they sometimes started asking people who were not trained.

On the issue of assessment. The guidelines explain that the woman should be tested at so and so on times. Because of that, most women who are even pregnant are not assessed for the same reason about lack of information sharing.

# Identification and coordination

Identification and coordination are happening well in the labour ward, antenatal care, and family planning postnatal, including at the TB.

The challenges are multiple health passports. Some women have special or different health passport books for ANC, family planning and ART. They aim to hide their HIV status. When we ask them, sometimes they answer differently. Ultimately, we realise but very late that the woman is HIV positive and has four to five passport books. In addition, as healthcare workers, we are unsure if it's necessary, but we are often quick. We do not check the health passport books. We start providing the services without finding out the HIV status of the woman. That's another challenge.

Another issue is documentation, sometimes, we have the correct health passport book, but we do not transfer the information from the health passport books to the registers even if the data exists in the health passport, and we fail.

# Mother-infant pair

Mother, infant pair and other services. All women, if not most of them, who are HIV positive and have infants are given a particular date to come and receive the services. We aim to provide services to the mother and infants comprehensively together. In addition, we combine the hospital cards for the mother and the infant, and we have no problem with that. Our challenges are that some services at our MIP clinic are unavailable. A woman can come, requiring family planning methods. The same woman may need a consultation with the doctor, the infant may not be feeling well, and the infant may require HIV testing. But you will find that the people providing services at the mother-infant pair clinic will only provide ARVs and check viral load, and we saw that must be a challenge.

There are also issues with giving appointments and dates to the mother and infant. There are also issues with poor documentation. If someone is reviewing, the cards will find documentation issues.

# Interventions

As I said, we currently have interventions in Blantyre and point-of-care machines in four sites. In addition, partners are supporting us in several ways. We have expert clients and ASOs who help in following up. We also have mock reports that guide us before the end of each month to determine how many infants we missed in that month.

The challenges are that we only have four sites out of 28 facilities in Blantyre, which are inadequate. In addition, the resources to ensure that these POC are working there are on and off, affecting adequate testing and available results. We further noticed that partners are only in selected facilities, which we feel is a challenge as other sites are not supported in EID—inadequate mentorship and supervision. We have adequate supervisors and mentors but resources for them to use to go to different health facilities to supervise and mentor there are not available, and we are failing.

For group 2. I should stop here.

**Chair of the day**: Thank you very much. Let us clap hands for group two.

**Team**: Claps

Chair of the day: If there are any additions from group two?

## Individual

On testing, there was another point on the mispairing of dates. We explained that.

Sometimes, the women have been given medication for a longer time with the infant, and later, the child misses the date for testing, so that is what we are trying to say.

**Chair**: Okay, so you are oversupplying the medication, right? Without considering other required milestones for testing?

**Chair of the day**: do we have other additions?

## Individual

The pink card is demarcated in months when healthcare workers are required to document, so it appears that it is still difficult for other providers to write on the right provided space. That also challenges some health care workers who may be reviewing such women to not the actual age and milestone to have the infant tested. For example, knowing when the infant has reached 12 months for HIV testing.

## Individual

Okay, I would like to add another point relating to the same. Sometimes, the same infant may need to receive immunisation, but the days for that infant to receive the immunisation are not yet due. Still, at the MIP, the infant is required to come. Somehow, we appear to burden the mother, which is very common for many women. Somehow, we do the MIP on Monday, for example, at Urban, and it is a challenge for the vaccine. They are required to come on a Thursday.

**Chair of the day**: thank you, group three.

**Team members**: clapping

## Individual

It may be time for a break before we have group three present, and the time has passed. I did not want to interrupt, but to save time, serving the food now and continuing the presentation while we have snacks will be fine.

**Chair:** Alright, we have someone bringing the snacks. Please forgive us for that. That is the plan on the agenda; there have just been a few delays.

**Chair:** group three

## Group 3

Group three: we have documented a few things. So, group members will add.

**Team:** Informal charts

Infants exposed to HIV enrolled in HCC. We were saying that enrolment is happening at the facility. For example, we had people from different facilities and others from rural and urban. An example from me from an urban facility is that we have had challenges enrolling HIV-exposed infants on day one at birth in HIV care because many nurses complained about having much work and for them to start documenting pink cards when the child is born. They felt it was adding more work. Just last month, the nurses had a meeting that they should be enrolling infants at birth. Although this has started, it is not 100% as some infants are not registered and are missed, so we are now enrolling the infants when they come at six weeks. While at rural, they indicated that they enrol infants at birth or check-ups. So, the main challenge is on day one, and enrolling these infants is challenging.

# HIV testing

On HEI testing, we feel this is happening very well; like at facility B, we also have a POC machine, and we can test them at six weeks. Those from rural, although they were able to collect samples, can collect the samples, and at least now the sample results are coming on time. However, we cannot test these infants strictly at six weeks at the required milestones, as most women come when the infant is three months old. Sometimes, this is because the information is not given accurately, or the women fail to capture it. So, some usually report that they should come when the prophylaxis nevirapine is finished, and if the nevirapine is completed when the child is four months old, that's when the mother comes.

However, even when the women come, sometimes the providers fail to identify the women. For example, other women come alone to the health facility and leave the infant at home, and sometimes the nurses do not inquire to find out and sometimes ask if the mother is breastfeeding. They report that yes, but they do not bother to find out about the infant.

We also feel that we have challenges at two years as we see that more infants are defaulting. Somehow, we see that at six weeks, it is better, and at one year, it is better, but at two years, it is worse.

# New guidelines

We are aware of 2p and nevirapine on the guidelines, and in our group, we had no nurses. We were health surveillance assistants and ART data clerks. We only heard that nurses went and got the training. We heard that nurses made initiatives that the facility should have the 2p. Still, I am not sure if they are now prescribing the 2P and for those from other facilities, they indicated that they do not know or maybe few people alone were trained on the new guidelines.

# Patient identification

On patient identification, we were saying that we identify them at different points, for example, antenatal and maternity; when the woman is being discharged, they are recommended to be tested. At the under-five, the HSA checks if the woman is HIV positive to check if the woman that has come is positive or not. We check, including at the OPD and postnatal. However, we still have challenges in all these points; although we can identify some, we know we are still missing a lot. For example, like us, HSA at the under-five, we are too quick and focus only on immunisation. It also depends on who is there; although all HSA have been trained on EID issues in this district, our focus is usually on immunisation. In addition, at the OPD, we miss most of them. Of course, guidelines talk about PITCT, but we miss most, and we still have challenges.

Mother and infant pair, since our group had people from different facilities, the practice appears to be the same. The mother's ART card and HIV care card for the infant are filed together. However, the challenge is that sometimes, people who come for the drugs are not the owners. They send guardians, and sometimes because of that, healthcare workers are unable to follow the milestones or sometimes a mother comes alone without the infant, and this depends on the nurse who is providing the services they do not ask about the infant. Later we note that the child defaulted, but the mother is still on ART. And that is a challenge that we are missing infant testing but are providing services to the mother. But we think MIP is there and even having specific dates for MIP services.

That is what we discussed if members from the group have additions.

So, we are remaining with the women's group.

## Group 4

women for us women that are breastfeeding group 4.

# Enrolling HEI in HIV care

## Group 4

On enrolling the infant in an HIV care program. We know that the infants are enrolled in the program when they are six weeks of age, and the challenge is that we do not receive results for HIV tests for the infants in good time. Sometimes, we receive the results after some time. For example, I went with my infant on the 20^th^ to have the child tested. I did not hear the results the same day, and I will go again on the 20^th^. I am also likely not to be told the results of learning from my previous experience. We go and receive the medication and go back home.

Another thing, although we did not write, we are not given nevirapine to administer to our babies all the time. For example, I was not given the medication for this child that I have, while I was given at seven months pregnant for my previous birth. However, I was going for my ANC but was not given the medication for this baby. When I was due to give birth, they referred me to QECH, still without the nevirapine, and here I was asked after two days when the baby was born if I had started giving the baby the prophylaxis. And I informed them that I did not have any. This happened when they called all women in one room, and it appeared that we, as women in that room, were all HIV positive. They asked me again, and I told them that I was not given, and they asked me why I did not remind the nurses. Then I wondered that, ahh, so I was supposed to remind, and so I was not given.

**Team:** laughing

But later then, I was given after two days. As a person, I thought that was a mistake, but I did not speak.

Team agrees.

## Individual

Accessing medication: as for me, I can receive the drug together with the baby. For example, we were both given a date to go on Tuesday, when the infant was tested for HIV, and I was also given the medication. We are, however, concerned with the way they handle us. We are unsure where the healthcare workers spend their night before coming to work to provide care to us. Usually, they come while drunk and shout at us. Sometimes, they tell us that they are unable to identify our files and sometimes ask us to sit somewhere for them to look at our files. We go home very late, around one or twelve noon, despite going early to the hospital and asking us to sit somewhere, and we don't even know what they do.

During the actual delivery, nurses are not around. We ask our guardians to call the nurses or sometimes beg the guards to call the nurses. Another issue is receiving results late; for example, my friend has an older baby than mine, and she is the first one who had the infant tested, but up to now, she has not received results but is giving the infant Bactrim.

When it is time for delivery, and we go to the nearest primary health facility, they tell us to come to QECH for delivery, ask us to find our own money, and tell us that transport to get to QECH is 15000 or 10000. And usually, you have not left anything at home by that time. As for me, I was lucky that my neighbour was close by. They are the ones who tried to get to QECH. My mother came and asked if there was an ambulance, and they indicated that there was no ambulance. Due to the limited time, we have discussed this so far.

Team: laughs

**Chair of the day**: we thank you for your input, but we are willing to give you more time to share your experiences.

Individual

in addition, sometimes, when they provide medication to us, healthcare workers are usually drunk and do not pay attention to what they are doing. They also shout at us if we miss the appointment date, and they speak in the way they want. In addition, when we are giving birth, the nurses are not there. The guardians are the ones that invite nurses to come whilst the baby has already been delivered. For example, the baby was already born when we called the nurses.

**Chair of the day**: Maybe some of you have remembered something.

Members. No, we have not remembered.

**Chair of the day**: The refreshments have now arrived, and I thought that while we are eating, we can finalise, and hope people document their questions.

## Individual

I have questions for groups two and three on the issue of guidelines, just that some of us did not reach that far. Both said that most healthcare workers were not trained. Usually, the practice, even with other programmes, is because of limited resources; not everyone is trained, or the training is done gradually, that few are trained, then the next lot is also trained. I am unsure whether we have an ART coordinator, but the facilities here now are trained. I am curious if any efforts were made for those trained to share the information concerning the new guidelines with the rest of the healthcare workers at the facility. Because it is recommended that after people have gone for the training, there should be an information sharing when they are back. For example, others use CPD and tell colleagues on group forums. They may not have the complete information that the person who went for the training has, but with the bit of information we want, they are likely to know critical things. For example, if we know such a woman with issues likely, one may have a high-risk exposed infant. So, we were trained where we are all coming from, so have we shared the information at the facility?

## Group 2

As for group two, we even mentioned that yes, few people have been trained, but challenges that there are; even if some people present the information, people usually want the incentive as well and usually that affects the implementation because they feel they did not benefit knowing that others received money to get the information. In addition, when there is training, there are different opportunities to capture the information and even those that went for the formal training, some the people that went for training do not fully understand the content, and when back, they do not share the information because they are not sure if they will share the correct information. Another issue is that some partners also take it up to orient the healthcare workers in the facilities, and sometimes those who went for the training leave it for partners to be responsible for training or teaching the rest of the healthcare workers at the facility. As a result, the knowledge is not shared.

**Chair of the day**. Thank you very much. I also had the same question and appreciated the response, so from there, we need to reflect on what we should do about this. Remember we have shared slides, and when discussing what we had already suggested in the previous workshop, we need to reflect then what we can do now. Because there are new guidelines to be implemented and as few people were trained, it is also possible that not everyone can go for training. We aim to implement what we are discussing here, but we cannot realistically involve everyone in how we should address these gaps. So, when discussing the next slide, we should reflect on how some of these issues will be sorted out. For example, people have an attitude requiring money for training. How best can we handle this?

**Chair of the day**: Are you satisfied?

## Individual

Yes, I just wanted to know what is happening when few people have been trained, and other people in the facilities are claiming not to know about the guidelines.

**Chair:** we also have another question here.

## Individual

I have a comment. Firstly, I want to acknowledge that in the recent training, very few people have been trained because of limited funding.

But we still expect that the few that have gone for the training should orient the rest of the healthcare workers because we cannot have everyone attend the training. We still need people on the ground to work, and our country is in an economic crisis, and funds are inadequate. Even the partners have limited funding. So, I would suggest that we should reinforce that those who have gone for training should orient their friends when they return. The next point we have noticed is a big gap, and I am repeating that at the ministry when they are making programs or policies, we previously used to have specific training for early infant diagnosis and not broad ART as it is now. If we look at how these infants are managed, we will note that most of these infants are managed by the HSAs, which we take as ART data clerks. But even if we look at the current numbers of these ART clerks that have been trained, they are very few. Is it not possible that we should implement as we used to previously? Apart from having training integrating PMTCT, ART, EID, ETC, can't we still consider having programme-specific training? And these can most target the support staff who are our back borne in implementation, for example, here in Blantyre. Simple things like feeling the Cards, usually nurses and clinicians are not available full-time at these clinics, but these people are always there. We need to appreciate the work that people are doing. Even if the nurses and clinicians have been trained, I can challenge you that people are not implementing the new guidelines. We have the resources, and 2P is there, but nurses are not screening and providing the 2p to high-risk infants. So, I wish if there are possibilities, we should still maintain the program-specific training and then give a broader picture of how the integration comes in; maybe things could change.

Of course, I am not very sure, but group two talked about mentorship; it is very accurate that the year is now ending, and I can't lie that we have supported or mentored any facility as part of EID. We have not done the mentorship because the partner keeping that activity, in their budget was not financed. Of course, even the one type of mentorship that supports integrated services that integrate different programs, although I am not well conversant with the Comcare application, I am unsure which indicators monitor EID. Because the idea is that whenever there is that kind of mentorship, there should be a person from HIV to support and mentor healthcare workers in those aspects. So, because the DNO and the coordinator are available may, will guide.

## Individual

**T**he indicators on this integrated tool for supervision are there, asking how many infants are enrolled, tested and have their results. It also looks at how many women are tested at the ANC and how many start ART at the ANC. They included those that give us a picture of how the facilities perform regarding HIV. However, there are still challenges that we have noticed with the integrated supportive supervision tool. It has a very long list and requires a commitment from the people doing the supervision. If no person knows or works in HIV, there is a possibility of missing them. And not all coordinators from all programs are present.

## Individual

Okay, thanks for clarifying.

Of course, there was also an issue that a woman and infant are given different appointment dates, and these women do not fail to go to the under-five, and usually, the healthcare workers that we have at the under-five are these HSAs. Then, the woman is also given another date to come to the facility for HIV services, and this is not good because we are burdening the woman to go to the hospital on several occasions. Now, this brings me back to the same point. We are sure that most women do not miss the under-five. Usually, there is the same HSA that is key to helping us to identify these women, and that's where there is a need for good training so that if these people are also providing services at the under-five, they should be checking at the under-five if the woman is living with HIV in the health passport books and if they have had the infants tested. These people require good training.

**Chair of the day**: Thank you very much. I hope we are noting the information. There are suggestions on training and the need to support the support staff, considering that they are primarily engaged in supporting the management of the clients and usually encounter clients.

**Chair of the day**: Do we still have other questions?

## Individual

I have a burning issue: Even though we have more program coordinators, we also have a facility in charge. If we look at family planning, currently, we are recommending that women coming to family planning should know their status and that they should be tested. We now have a hiccup. If we reinforce the testing of these women, most women will opt not to come for the family planning, and we will lose out on the women coming to the facility. And of late, we have realised that we are identifying many infants exposed to HIV that have now been infected but primarily identifying them through family planning. But we are also aware that when we intensify testing in family planning, more women will stop accessing family planning, yet at the same time, when we test these women, we later find more infants exposed to HIV. There is some conflict. When we leave the woman without recommending the testing, it will give her freedom. Still, we are harming the infant and the mother together in case they are HIV positive, and when we recommend, we may even lose the coverage of family planning. However, almost 80% of infants that we find through women at the family planning are usually living with HIV. So, we should still be reminding the healthcare workers that they should still recommend the testing, not just leaving that the women will stop accessing health services.

**Chair of the day**: Thank you very much for that outstanding contribution.

## Individual

As the other member has explained, we need to realise that when deciding at the senior level, we need to note that every manager makes favourable decisions for their program. If the communication has not been given very well to implementing people, we confuse people. I also remember when I used to work at K health centre, we used to say that everyone accessing family planning services should be tested. One, it was delaying the process, and we also noticed a reduction in the coverage of family planning. So, we must consider how we provide the service carefully. No, we can't say that we have reached that point of mandatory testing. Still, we saw that, to some extent, some healthcare workers took it as compulsory testing. Women who also accessed family planning took that as long as they came for family planning, they thought it was also automatic that they would be tested no matter what. So, I feel it is essential to package our information. The women should understand why we recommend the testing to those accessing family planning. Workers from the testing room used to open the books of the women in that area, and everyone knew that people were researching HIV status. And so, if we have not appropriately explained, one program can suffer, and so for us who are looking at HIV management and those looking at family planning, we need to be speaking to one another.

**Chair**: thank you very much for that input

## Individual

I also want to agree with the previous speaker that there is an issue of cervical cancer and family planning. It appears that women are being forced to be screened for cervical cancer, failing which, they will not receive family planning methods. So, we have noticed that there is also much confusion, even for women. For example, you find women making much noise whilst waiting for the services, and when we ask what the problem is, they will notify us that we have been told that we will not access family planning if we do not go for cervical cancer screening.

**Team:** Laughing

## Individual

I remember when those women said that I went to cervical cancer screening to enquire about the issue. The nurses indicated that we were only recommending them to have screening, but the women said no, they were forcing us. And they told us that we were not receiving the family planning if they were not screening us. I went to the family planning to understand. I noticed that every woman receiving family planning had cervical cancer screening, so I thought everyone was interested. But you would see that. Indeed, the healthcare workers were somehow coercing information that there would be no family planning if no screening were done. So, they need to work together and see how best the data is packaged for the clients to make their own informed decisions. But you also note that partners influence this because they have targets to show that their program is working well, i.e.…...)

## Individual

That must be done at the top level.

## Individual

We have also noted the requirement for women requiring HIV services that every woman who is accessing HIV services also needs to be screened for cancer.

## Individual

On that one, I will also reflect on the people who come to supervise us. I will not mention who came to supervise us, but they said facility D was not doing well on the screening when they arrived. They said that for every woman, before you assist them with the service they have come for, you must send the woman for screening, and they shouted at us a lot. They indicated that cervical cancer is killing many women, and they said that healthcare workers have a role to play when providing services, so this kept us on a fix.

**Chair:**

I love the conversation because there is a need for women to be assisted, a balance that our work should be practical and the women to be helped effectively. While respecting that our clients are autonomous, they have a final decision at the end of the day. I have loved this element because we also have active client identification and tracking, which emerged from a formative workshop. I may speak at the end to accommodate others who still have questions about the presentations.

## Individual

Just a comment on the new guidelines on identifying high-risk exposed infants and the coming in of 2P. It appears there is a shortfall in the communication. We have seen that other people are crucial and could have been prioritised to be equally trained. When these people receive the orientation, they do not see the benefit, are not focal persons of the programme, and do not receive any money. And there is a need for a mechanism if there is a mistake. One should be tracked and answerable. If we can develop that mechanism, we may help people avoid shortcuts in their work. The other issue is what if we have TOR and we paste in so many places that others who have not been trained should be guided and read as reference materials?

**Chair:** Thank you very much. We should reflect on these vital and critical points while refining the intervention.

## Individual

I want to echo group four. They talked about the delays in accessing blood test results and late enrolment of infants exposed to HIV, and I feel what they have said is the truth, and it is happening. And sometimes, that occurs because of the system, and people on the ground are the ones who appear to be at fault.

**Chair:** maybe the … manager can share your thoughts on the testing system

## Individual

Of course, we have challenges indeed, but sometimes I think providers take advantage of that the system has gaps because sometimes results are sent back to the facilities, but for the providers to give the results appropriately, it is not done quickly.

Of course, previously, we have had a stock of cartilage used to test infants, but we have been trying to source from other districts. So, it is the same way the challenges are with Viral load. You may find that we have printed and sent so many results, but we hear here that it is also challenging for the facilities to process giving back the results to clients. So, it is sad that the moment they hear that we have a stock of cartilage, the provider does not follow up to check if the samples are processed and if the results have arrived at the facility.

## Individual

Maybe the … manager wonders why we usually ask her about results; these are the issues we face. We encounter other women who had to cross rivers coming for their appointments and are eager to know the results for the infants on that day. It is always challenging, so we keep pushing you.

## Individual

Still, we want the manager to explain further; when she indicates the providers, who is she referring to? Is it we who collect the samples? Or those that deliver the samples. Because as for us, we only collect the samples, pack them, and give them to the riders for health that transport them to the lab for testing. But usually, it even mostly takes two months before we hear back the test results. As opposed to previously, when we were sending samples to facility Y, it was not even taking two weeks so they would collect samples this week, and we would receive them by the following week. But now, as how one of the women has said. For the sample taken on 20^th^ December, we should expect results after 20^th^ January 2023. The challenge is that we can't tell a client to come next week for results, but in a very hopeful manner, we inform them to go again at their next appointment with the hope that the results may return. For example, even if we take the sample logbook for Facility H, you will find many gaps. Could you clarify what you mean by providers?

## Individual

Before the manager comes in, I want to agree on one point: sometimes, we, as providers, contribute that results should come late. I am saying this because we can collect a sample today and pack it on Monday, and we should not expect the sample result to come out fast because we have delayed it. Had it been that our practice is different, when we collect samples today, pack them tomorrow, and the raiders person comes on Thursday and finds them packed and carries them. So, we need to be reflective. We, as providers, also contribute.

## Individual

To add to what has just been explained, when looking at it, we should not start pointing out that it is a problem for the lab or the facility. As far as I know, it is our problem because sometimes the lab can process and share the samples with the facility. But at the facility, do we have an establishment or system to say when the results should reach a certain point and how the results should be handled? And who is supposed to handle them? And who is entering them on the pink card to ensure the results are ready for the mother at the next visit? So, it is the whole system, and this should be reflected in care pathways.

## Individual

**E**ven though I have come in late, I have followed. These days, our focus is more on ART, and we have left behind the children. Blantyre district is quite Big. We have facilities A, B, and C, as well. But for example, facility B is quite a big facility. I did not expect these people to collect and send samples for processing. They need POC. We need to fight that. We have seen that we used to fail when we were using molecular labs and sending our samples elsewhere, and sometimes, when we have run out of cartilage, we can buy ourselves. We reach out to the partner to help us. But these will keep pulling us down; imagine when we go to big meetings, they put Blantyre district just like that, forgetting that we also have other big facilities like private hospitals, which offer services to more clients, just like the central hospital. If you go to District K, you will find that most facilities have POC equally M, but they put them on a high rank. In one of the presentations, I also saw that enrolment should be done on 0, not six weeks. I hope we corrected each other. The woman doesn't understand the testing milestones if we miss birth enrolment. Usually, they do not know when they will need the infant again at the facility, so enrolment is paramount and discussing with clients.

## Individual

Whilst you are there, the other groups also reported that those who gave birth at the referral hospital are not enrolled in HIV care, and they go to their respective facilities without knowing what to do, and sometimes they come even late for the six weeks’ check-up.

## Individual

It is good that you mentioned we have a referral presentation tomorrow if you have seen the agenda. I was also asked to bring maternity and HIV care clinic registers for us to discuss and understand what is going on. I also heard among us that somehow, we leave support staff without training. Those actively working here are the support staff, and those you see come without a pink card if they are off that service is closed.

## Individual

sometimes, we also have other women that come at one week whilst they have not administered medication, and when you ask them, they inform you that at QECH, they did not teach me.

## Individual

It is cross-cutting because we also receive women referred from the health centres without prophylaxis given during their ANC.

## Individual

on that one, a lady explained that she was not given the prevention when she was referred, and she stayed for two days without receiving it. It means she was referred from a facility without medication, and even at QECH, she was not given the medicine.

**Team,** we need to look at the whole system and find solutions.

## Individual

Before the chair comes in

**Chair**: okay, I do not want people to start saying that time is up

## Individual

We deliberately invited coordinators because we identify women living with HIV from these areas and programs, so there have been issues, for example, having a woman living with HIV delivering by her self-meaning there are no preventive measures taking place, increasing the risk of women to acquire the HIV infection including women being shouted at that is why we invited these key program coordinators as well. Because, just like the EID program, we can't identify these women. Indeed, if we look at Blantyre district, there are very few infants that we enrol in HIV care program right at birth and very few women at one week. So, if we miss such women without giving them nevirapine at the ANC and check-ups, we also miss the infants.

**Chair:** Thank you all for your input. I think it was good that I just gave an overview of what I found in the study, and I am sure you know the context and the experiences of how this happens, which is a good thing, which I think it is good because we see a need to address the gaps.

Several issues have been clarified that I also had questions on. In group one, there was a point that they feel 90% they are doing well in identifying women. I would like to know what identifies these women. You mentioned where these women are identified, but I would like to know who identifies them.

Group one, are you able to remember:

## Group 1

Group one: so, I will give an example of under-five; it means HSAs are the ones that identify these women. HSAs sometimes can screen in the passport book, checking the HIV status of the woman, and if somehow, they have noted that the woman is positive, they take them aside. Nutrition is a must; in short, we have mentioned several places, so different providers in those places are the ones that identify such women, labour wards, and midwives.

**Chair**: Here is a reason I have asked: from what we observed in the study I explained, we saw that most women were not identified. This nurse retired, but she can be my witness; even now that we are collecting the baseline, we still meet many missed women. I will link this point more with what another member explained: here in Blantyre, we are not enrolling infants exposed to HIV in HIV care at birth. If we are asking why, you will note that healthcare workers are not actively identifying a woman that this woman is living with HIV, and we should enrol the infant in HIV care. Regarding how testing is going, it is good that we have also seen how facilities with point-of-care machines are handling the problems. I would want us to have a best-case scenario from those facilities to make a case like others have said to lobby for more POC in Blantyre by showing that we can have several successes with POC. Few people may agree with me because they have also been studying with me and have been on the ground to note some of the issues you have even pointed out. But if we also focus on POC and MIP, as you have said. Of course, for some people who were there in the focus group discussions in the facilities but also at the formative workshop, you are all witnesses that we were able to ask six women to come for testing, and because of the capacity of the point of care machine we were sending them back to go again for testing the following day. But when we scheme through the rest of the days in the week, you would note that there was usually no one again coming for testing while we had put several women on one date, but later on, we were also sending them back. I am also pointing out these issues to what you have explained today as part of your experiences. So, if we are talking about the point of care machine, do we only refer to giving the same date? You have well put it that even other services, including family planning. So, we need to reflect. Should we say it has to be the same date for a woman to collect ARV and test the infants only? We should also reflect on the issue of the capacity of the POC. If we have returned that woman because we have more women to be tested with the POC, for example, six women, should we say we are doing MIP? We should also remember that the POC can also have challenges, and if the women came for testing, testing usually takes one hour for the result to come out. I thought those issues would come out, and sometimes a woman is sent for testing at 11, and maybe the machine should produce an error or even an HIV-positive result requiring the infant to be tested again. What do we reflect on this? The time the mother spends at the facility. And waiting for the result of POC processing, how are we integrating this woman into other services, for example, family planning on this day? So, different questions are focusing on looking at our context: do we think MIP would be possible because all services are provided in one room? And if yes, which room is that? If we still do not have the resources, what could we do to provide better MIP? I hope you can understand the thinking I have, right? I would even be among the people recommending POC, but how best are we using it?

**Team**: Yes

If you look at the program, we also discuss sustainability issues and reflect on scaling up. But at the end of the day, we need to tell our story to show that we have closed all the gaps and that there are no loopholes and the impact. I hope you get what I mean. I am pointing these things out because I did not hear them clearly in the discussion, the problems are mentioned but we can further reflect on them. Still, I am equally recommending you all that you have done an excellent job of pointing out what life is like out there and most things you have mentioned. I have also seen these in the preliminary study, so I am pointing them out because I did not hear them more in the discussion. So, for our POC and MIP, we have said that the other challenges are that the day the infant is offered testing and the day that the infant is receiving immunisation are quite different days, and we also heard others saying that they have specific days for MIP. And course, this does not usually happen, but what do we do if we have seven infants coming, all of them on an MIP day, but our POC machine does not have adequate capacity? Do we still give all of them that appointment? If all seven have come on that day because it is the MIP day, will we be able to give all the services? Because we already know that our POC will not have that capacity with that number because there are other days, these can agree with me that we can have women six. So, I want us to reflect on our context, and we also need to remember what the …. said; as much as policies are made at a high level, there is a reason I came here for a discussion. Still, I decided to understand and discuss with you the ones who understand the context better. So, what will we do as much as the guidelines are there? I have noticed a few things that vary significantly with different contexts in Malawi. For example, some facilities have POC, and others do not.it is not one size fits all. So, when the guidelines come to the district level, can we reflect on how our facility is going to adopt the guidelines to ensure that at the end we meet the guidelines in the end? So, I am putting all these things forward. I do not want to impose anything. If you feel some of these things are important, let us explore how they should be done. I am not refusing that other facilities that do not have POC; yes, it is true when we are planning for POC. When the POC goes there, they should already know how best to utilise the POC. I hope you get what I mean.

So, all these issues we need to reflect on, yes guidelines say we need to identify women at birth, so the question is, are we identifying women at birth? If we are, why are we having low enrolment of HEI at birth? And if we can identify women at six weeks, now the question is, why are we having other women not tested? Unfortunately, I do not see the actual testers from the labs but from the health centre. I am sure we will get their input. What time do we send the women to the lab for testing, and then they must wait one hour? Then, the same woman has to factor in their need to receive family planning. Also, how do we look at issues of women moving in all those directions queuing? How do we define MIP/ is it just about dates? And we recognise all these now that we do not have proper infrastructures, but do we have other opportunities to provide integrated services? For things to move forward at the end of the day, we will have another important issue that we also need to consider, are we there to provide service, right?

**Team:** Yes

'We also need to reflect on the measure that the person is an autonomous being because sometimes it happens. I will give you an example of the facilities we provide each other handover. We need to reflect on how we engage everyone taking part in the handovers to refine what was suggested at the formative workshop. Because we read we had so many women and so many births, so many women living with HIV women, etc. and then maybe the others are not listening, others are on the phone, etc. if we can create a platform of working together if we hear that there is PMTCT woman then another nurse already is checking if the woman is documented in the HCC and another nurse is looking at the cards and working as a team. Not all the time's nurses on duty enrol the infant; others say they were busy, and others say they thought those on a day shift would document the woman, as one group has reported. But those people gave each other handovers but still missed the women. So, I have been reflecting on these because we discussed them during the baseline workshop and reflecting on current experiences as you have done now so that we can still understand the context.

Other challenges, like documentation and even the same handovers, are compelling. Suppose someone is reporting that there was a woman. For example, they are doing very well. In that case, they keep a pink card in the maternity, but at times you still find that other women were missed, but at the same time, handovers were done at that platform. I agree that these nurses should know how to document. I am not refuting that HSAs can't support it, but if a woman is giving birth in maternity and the nurse is the one delivering. Do we need a HSA not even there to come and document? If we leave it like that, we may have many errors.

I am sure that even on that handover, if the nurses support each other well, the documentation issue can be noted, and those having challenges can be supported by others.

Because of time, let us reflect.

There are issues of training. We cannot afford to train everyone, but what do we do while we are in that context?

It's the same issue of documentation and identification. I will give you an example. As part of the study, we are using ART numbers to avoid duplication because we anticipate that women can change health passport books, but it's a struggle to identify the ART numbers.

Sometimes, you identify a woman who will present a different book in agreement with what the other group presented, one just for family planning, so it is true what you are saying. Amidst women changing books, what are we going to do? Others said electronic books were going to be ideal, but because of the resources we do not have, what will we do?

The groups have discussed a lack of adequate mentorship and supportive supervision. Given our lack of resources, will we sit down? Our coordinators are very instrumental. They have found ways to improve reporting, but we still need supervision. Is there anything else we can do?

I am just pointing out that when we discuss, we should reflect on that so we can go in our groups and build on the key gaps from the discussions and what we discussed in the formative workshop.

Even those available in the formative workshop should support the discussion now and give context for some of us who were not there. I will share all these reflective questions based on the gaps you have said from the formative workshop and study findings.
