# Supplementary material for: Developing an intervention to improve early infant HIV diagnosis service uptake among postpartum women in Malawi’s primary healthcare using a co-designing approach with stakeholders
Source: PLOS Glob Public Health. 2025 Apr 22;5(4):e0004426. doi: 10.1371/journal.pgph.0004426 (PMC12013899; doi:10.1371/journal.pgph.0004426)
Supplement: Attachment — Submitted filename: Review_points.pdf [file pgph.0004426.s010.pdf]

Journal : PLOS Global Public Health

Manuscript title: *Developing an intervention to improve early infant HIV diagnosis service uptake among postpartum women in Malawi's primary healthcare using a co-designing approach with stakeholders*

This interesting research study uses co-design to identify context-specific determinants and design appropriate implementation strategies. I enjoyed reading and learning from your manuscript. Below are some comments and suggestions for improvement.

Abstract

Overall, the abstract does not entirely clarify the context of the collaboration mentioned, the details of the setting, or the stakeholders. Please add this information to help the reader better understand your study.

Line 31: To be more apparent to the reader, please specify who is underutilizing health services after birth.
Line 32-34: Clarify the context in which this study was conducted. Why did this collaboration happen(is it part of an ongoing study?), who are the stakeholders, and what kind of EID services in Blantyre (in health facilities?, public/private?, and how many)?
Lines 41-46: When specifying the challenges, clarify the subjects to whom these challenges apply. For instance, who suffers from stigma or has clustered work shifts and minimal supervision? It would be a mistake to assume the readers can guess these.
Line 46: the abbreviation HCW is not defined anywhere in the abstract; please correct it.

Introduction

Overall, reduce the number of abbreviations to provide a smooth reading experience.
I recommend adding data about EID coverage in the study setting.
Line 66: it's unclear if the estimated 72.3% and 82% are related to postpartum or ART care retention. Please clarify.
Line 82: define MRC
Line 76-86. The information in the paragraphs suggests that the present manuscript responds to a previous study's findings. If that is the case, this should be clearly stated so the reader can understand the context of your study.

Material and methods

Overall, the content needs to be reorganized. I recommend first describing the program/intervention and clarifying the study setting and design. The results section is too long; it would benefit the reader if the information could be further synthesized. I couldn't read the content of the figures in PDF format; the quality was poor.

Lines 92-94 read: "*We co-designed the intervention to be evaluated at one urban and one rural study site, selected based on location, catchment population, and the presence or absence of POC machines*."
I don't understand this text. Does "we co-designed the intervention to be evaluated" means "our study was conducted"? The way it is written, it seems that this co-designed intervention will(in the future) be evaluated at one urban and one rural study site. Were the workshops conducted at these two sites? Are the stakeholders from these two sites? Please clarify the setting of the present study.

Lines 93-97: How many urban and rural primary health facilities exist in Blantyre? You mentioned that they were selected based on location, catchment population, and the presence or absence of POC. Please explain more about these selection criteria so we can understand how you chose your study settings.

Lines 105-118: I am confused. It states, " *Our program theory*…". What program has been referred? I suggest describing the program first and then describing the theory. If

your “program” is the same as your “study,” I suggest harmonizing the language to avoid confusion.

Line-120:123. In this section, please describe only the study designs. For instance, a descriptive study design of the stakeholder analysis content and a qualitative study design for the stakeholder’s discussion are used. Information about the intervention steps should be removed and added to a section describing the intervention/program.

Lines 126-141: The transition to the “recruitment and enrolment of stakeholders” section is weird because the workshop and its participants were not previously described in the paper. I recommend creating a section at the beginning of the methods section to describe the program/intervention(which seems to me to be the workshops) so that when we read the setting, study design, and all other sections, it is clear what we are talking about.

Lines 154-167: I recommend moving the information about the workshops' characteristics to the new program description section I suggested above. In the data collection section, please describe when the data was collected, who collected it, the instruments used, and the data types collected.

Lines 169-193: Again, this is part of program description. I recommend moving this information.

Line 240: I suggest using the exact wording as **lines 224-227** to facilitate the information flow. Instead of “client identification,” I suggest writing “Theme 1: enabling client identification”. The same goes for the other four themes.

The results section is too long. Please summarize it as much as possible.

Discussion

Lines 582-588: As mentioned above, the selection of study sites needs to be clarified. I am confused about what you identify as study sites, the description of the workshops, and the discussion surrounding these sites.

Line 586-594: By reading these lines, it seems that the EEH was tested in the two study sites. Is this the case? Or have these conclusions been extracted from the participants of the workshops? Please clarify.

The only study limitation cited in your discussion is the exclusion of one group of stakeholders. I encourage the authors to consider other possible limitations to conducting such a study.

It would be essential to focus your discussion on the need for researchers to use co-design approaches to design context-specific implementation strategies. Besides the findings drawn from the workshop participants, what have you learned as a researcher when applying a co-design approach? What advantages and disadvantages can be pointed out?

What implications do your findings have from a research and policy perspective in Malawi?
