# Supplementary material for: Developing an intervention to improve early infant HIV diagnosis service uptake among postpartum women in Malawi’s primary healthcare using a co-designing approach with stakeholders
Source: PLOS Glob Public Health. 2025 Apr 22;5(4):e0004426. doi: 10.1371/journal.pgph.0004426 (PMC12013899; doi:10.1371/journal.pgph.0004426)
Supplement: Attachment — Submitted filename: Comments.docx [file pgph.0004426.s009.docx]

The manuscript describes an important topic in relation to early infant diagnosis of HIV. Detailed information was provided on how this study was conducted. A few comments

1. Introduction could be strengthened with data on HEI cascade within Malawian contexts. Such as Numbers of HEI born at a particular time frame. Of those, what proportion were brought in for testing (say at 6 weeks). Of those what proportion got tested. Of those, what proportion received results. This information could set a stage to see the gaps the authors are trying to explain they do exist. This data was not provided besides qualitatively explaining existing gaps. Quantifying the magnitude of the problem would be beneficial.
2. Did Malawi adopt the WHO EID testing, or it has its own guidelines? How is the testing scheduled? Do the authors considers all testing before 18 month and EID? Or there is a time limit this is being referred? Defining it somewhere (introduction or methods) would be helpful
3. Line 74-76,There is a need to disentangle testing by age. If I am not mistaken PCR is as stated 4-6 weeks, then at 9months on is antibody testing. If the intention was to only consider PCR, there was no need to use 18- months threshold, else states other testing modalities after 6 weeks.
4. 4 POC in 28 facilities is difficult to achieve effective infant HIV testing. Compounded by the time it take to test single sample. What did not come out during stake holder’s discussions is the use of dry blood spot and sending samples to central lab. Would you consider this as option in your recommendations? How feasible is this?
5. Stating HIV positive women as in line 78 is considered stigmatizing. Can change to women living with HIV throughout the manuscript.
6. Analysis section was all about qualitative aspect of the project. Table 1, however, has quantitative data. It was not described in the analysis section. For example, while it is intuitive to think the numbers reported after median are lower and upper quartiles, not every reader is aware of. It would be appropriate to detail analysis section and document in the table what do those numbers mean.
7. A little difficult to follow certain information. For example, supplemental file S2, what do those H, Y, N, LDL mean? Having a footnote would be helpful.
8. If formative research was aimed at validating validate Suwedi-Kapesa’s work. Do you want to say something about this validation in your discussion or at least concluding remarks?
