# Supplementary material for: Developing an intervention to improve early infant HIV diagnosis service uptake among postpartum women in Malawi’s primary healthcare using a co-designing approach with stakeholders
Source: PLOS Glob Public Health. 2025 Apr 22;5(4):e0004426. doi: 10.1371/journal.pgph.0004426 (PMC12013899; doi:10.1371/journal.pgph.0004426)
Supplement: Attachment — Submitted filename: Response letter.docx [file pgph.0004426.s012.docx]

Dear Dr Joel, Msafiri Francis,

On behalf of the research team, I would like to thank you and the reviewers for your thorough and constructive review of our manuscript. Your valuable feedback has enabled us to strengthen our paper. Please find below our responses to the queries raised with line numbers for reference to your suggested edits. As requested by the editor, we have submitted two versions of the manuscript: a marked-up copy and an unmarked copy. Our responses to the editor's queries are presented after the reviewers’ comments below.

| **Reviewer1** | **Response** | **Section revised** |
| --- | --- | --- |
| 1. Introduction could be strengthened with data on HEI cascade within Malawian contexts. Such as Numbers of HEI born at a particular time frame. Of those, what proportion were brought in for testing (say at 6 weeks). Of those what proportion got tested. Of those, what proportion received results. This information could set a stage to see the gaps the authors are trying to explain they do exist. This data was not provided besides qualitatively explaining existing gaps. Quantifying the magnitude of the problem would be beneficial. | We have provided an overview of EID uptake at six weeks in Malawi from 2020 to 2023.  We have also provided data on infants exposed to HIV cascade, focusing on the findings in the setting this study is building on | Introduction  Line 81- 82  Line 86- 87 |
| 2. Did Malawi adopt the WHO EID testing, or it has its own guidelines? How is the testing scheduled? Do the authors consider all testing before 18 month and EID? Or there is a time limit this is being referred? Defining it somewhere (introduction or methods) would be helpful | Malawi has adapted the WHO EID testing, offering POC in a few facilities and using near POC testing in the rest of the facilities. This has also been clarified in the methods  We have defined that our focus is on 6 weeks of testing | Line 82-84  Line 99-100 |
| 3. Line 74-76, There is a need to disentangle testing by age. If I am not mistaken PCR is as stated 4-6 weeks, then at 9months on is antibody testing. If the intention was to only consider PCR, there was no need to use 18- months threshold, else states other testing modalities after 6 weeks. | Thanks for this suggestion. However, the WHO has updated the testing by age: PCR is extended to children under 18 months, starting at 6 weeks and 9 months (WHO 2021).  Malawi briefly tried the 9 months but returned to serology testing  We have added that for 9 months the recommended testing is also PCR. Our focus is on the first six weeks | Line 78-80 |
| 4. 4 POC in 28 facilities is difficult to achieve effective infant HIV testing. Compounded by the time it takes to test single sample. What did not come out during stake holder’s discussions is the use of dry blood spot and sending samples to central lab. Would you consider this as option in your recommendations? How feasible is this? | Thanks for this addition; the discussion now emphasises using DBS where POC is impossible.  However, there was an oversite not including this finding. Stakeholders reflected on avoiding sending back clients and recommended using DBS, and we have added this  It is feasible and was also recommended in the previous study we are building on. | Line 609 -610  Line 406 |
| 5. Stating HIV positive women as in line 78 is considered stigmatizing. Can change to women living with HIV throughout the manuscript. | Thank you very much for this observation. We have worked on this, including the HIV-exposed infant as recommended by Waters et al. 2022 on recommended HIV terminology | Throughout manuscript |
| 6.Analysis section was all about qualitative aspect of the project. Table 1, however, has quantitative data. It was not described in the analysis section. For example, while it is intuitive to think the numbers reported after median are lower and upper quartiles, not every reader is aware of. It would be appropriate to detail analysis section and document in the table what do those numbers mean. | Thanks for this observation. We have added that we analysed the demographic characteristics of stakeholders using Excel and Stata 14, describing the frequencies, proportions, and median (the number that divides the data set into two halves, 50% below and above it).  And added in the results the overall median and age range | Line 237-239  Line 262-265 |
| 7. A little difficult to follow certain information. For example, supplemental file S2, what do those H, Y, N, LDL mean? Having a footnote would be helpful. | Thanks for this comment.  We have added the meaning of the letters in the footnote | S2 |
| 8. If formative research was aimed at validating Suwedi-Kapesa’s work. Do you want to say something about this validation in your discussion or at least concluding remarks? | Thanks for pointing out. We have given remarks that the findings confirm Suwedi Kapesa’s  Some findings have been discussed and related to Suwedi-Kapesa's findings | Line 555 and 556  Line 576, 610-611 |
| **Reviewer2** | **Response** | **Section revised** |
| Overall, the abstract does not entirely clarify the context of the collaboration mentioned, the details of the setting, or the stakeholders. Please add this information to help the reader better understand your study.  Line 31: To be more apparent to the reader, please specify who is underutilizing health services after birth. | We have clarified that women and infants underutilise health services | Abstract, line 31 |
| Line 32-34: Clarify the context in which this study was conducted. Why did this collaboration happen (is it part of an ongoing study?), who are the stakeholders, and what kind of EID services in Blantyre (in health facilities? public/private? and how many)? | Thanks for this note. We have clarified that stakeholders were healthcare workers, service users and non-governmental organisation workers from seven public facilities and five non-governmental organisations and that the study builds upon a previous study | Line 32-35 |
| Lines 41-46: When specifying the challenges, clarify the subjects to whom these challenges apply. For instance, who suffers from stigma or has clustered work shifts and minimal supervision? It would be a mistake to assume the readers can guess these. | We have specified the subjects that clients and healthcare workers' perspectives on stigma, HCW clustered work shifts, and there was minimal supervision among HCW | Lines 44, 45 and 48 |
| Line 46: the abbreviation HCW is not defined anywhere in the abstract; please correct it. | Thanks for noting this. We have spelled HCW | Line 33 |
| Overall, reduce the number of abbreviations to provide a smooth reading experience. | Thanks. We have reduced the number of the following abbreviations. We have dropped these abbreviations (MIP, HEI PMTCT, NGO, ANC, HDA, HAS) | Throughout manuscript |
| I recommend adding data about EID coverage in the study setting. | We have added EID coverage in the study setting | Line 86- 87 |
| Line 66: it’s unclear if the estimated 72.3% and 82% are related to postpartum or ART care retention. Please clarify. | Thanks for noting this.  We have clarified by providing both statistics for postpartum and ART | Line 68-71 |
| Line 82: define MRC | We have defined the Medical Research Council (MRC) | Line 96 |
| Line 76-86. The information in the paragraphs suggests that the present manuscript responds to a previous study’s findings. If that is the case, this should be clearly stated so the reader can understand the context of your study. | Yes, it does; we have included that this paper builds on the Suwedi-Kapesa et al. evaluation. | Line 93-94 |
| Material and methods Overall, the content needs to be reorganized. I recommend first describing the program/intervention and clarifying the study setting and design. | Thanks for this observation. To our knowledge, we have reorganised according to the recommendation.  We have first described the adapted theory (The theory is not an intervention itself, but it is what guided to develop the intervention)  And study setting and design have followed | Line 114- 204 |
| The results section is too long; it would benefit the reader if the information could be further synthesized  The results section is too long. Please summarize it as much as possible. | We acknowledge this, and we have synthesised from lines 415-857 down to lines 292-535 | from line 415-857 down to line 292-535 |
| I couldn’t read the content of the figures in PDF format; the quality was poor. | Thanks for this. We have edited |  |
| Lines 92-94 read: “We co-designed the intervention to be evaluated at one urban and one rural study site, selected based on location, catchment population, and the presence or absence of POC machines.” I don’t understand this text. Does “we co-designed the intervention to be evaluated” means “our study was conducted”? The way it is written, it seems that this co-designed intervention will (in the future) be evaluated at one urban and one rural study site. Were the workshops conducted at these two sites? Are the stakeholders from these two sites? Please clarify the setting of the present study. | Thanks for pointing out this. There are three things that we have clarified.   1. The intervention was developed to be evaluated at two primary facilities building from the Suwedi-Kapesas et al. study 2. The stakeholders were from the above two facilities and other primary facilities, 2 urban and one rural, with similar characteristics, making a total of 5 for diverse perspectives and possible scale-up. However, managers and coordinators from the district health office who look after all 28 facilities in the district were engaged. Plus, the one referral hospital where primary facilities refer clients and the five non-governmental organisations 3. The workshops were conducted at a neutral venue away from the sites at a conference in the district. | Line 172- 198 |
| Lines 93-97: How many urban and rural primary health facilities exist in Blantyre? You mentioned that they were selected based on location, catchment population, and the presence or absence of the POC. Please explain more about these selection criteria so we can understand how you chose your study settings. | There were a total of 28, 9 urban and 19 rural. And only four facilities had POC. The selection for this study was built on the Suwedi Kapesas study sites. The additional sites looked for similar characteristics to the initial study sites (Suwedi Kapesas’) | Line 172- 198 |
| Lines 105-118: I am confused. It states, “Our program theory…”. What program has been referred? I suggest describing the program first and then describing the theory. If your “program” is the same as your “study,” I suggest harmonizing the language to avoid confusion. | Thanks for pointing out this. In the Medical Research Council, it is called a program theory. However, we have called it adapted theory, meaning the theories that guided the intervention development during the workshops  We have described all theories used and how they were used along with the workshops, and we have harmonised the language | 114-167 |
| Line-120:123. In this section, please describe only the study designs. For instance, a descriptive study design of the stakeholder analysis content and a qualitative study design for the stakeholder’s discussion are used. Information about the intervention steps should be removed and added to a section describing the intervention/program. | Thanks for this suggestion. We have described the study design as  a descriptive study design of the stakeholder analysis content, and a qualitative study design for the stakeholder’s discussion are used | 201-202 |
| Lines 126-141: The transition to the “recruitment and enrolment of stakeholders” section is weird because the workshop and its participants were not previously described in the paper. I recommend creating a section at the beginning of the methods section to describe the program/intervention (which seems to me to be the workshops) so that when we read the setting, study design, and all other sections, it is clear what we are talking about. | We agree. We have described workshops in the adapted theory and the desired stakeholders to engage at workshops, and we have reorganised the sections  The workshops were not interventions; they were just a means of engaging with stakeholders, as described in the adapted theory section, as recommended. | Line 114- 218  Line 141- 167 |
| Lines 154-167: I recommend moving the information about the workshops' characteristics to the new program description section I suggested above. In the data collection section, please describe when the data was collected, who collected it, the instruments used, and the data types collected. | We have moved the workshop characteristics to the adapted theory | Line 141- 167 |
| Lines 169-193: Again, this is part of program description. I recommend moving this information. | We have moved to the adapted theory | Line 118-123 |
| Line 240: I suggest using the exact wording as lines 224-227 to facilitate the information flow. Instead of “client identification,” I suggest writing “Theme 1: enabling client identification”. The same goes for the other four themes. | Thank you for this suggestion we have changed all themes | Result section  Line 289,  352, 396,  454, 487 |
| Discussion Lines 582-588: As mentioned above, the selection of study sites needs to be clarified. I am confused about what you identify as study sites, the description of the workshops, and the discussion surrounding these sites. | We have clarified this in the methods | Line 172- 198 |
| Line 586-594: By reading these lines, it seems that the EEH was tested in the two study sites. Is this the case? Or have these conclusions been extracted from the participants of the workshops? Please clarify. | Thanks for seeking clarification. These interpretations are based on the participants of the workshops | From Line 600 in the discussion referring to the results section from line 359-369 and from lines 443- 453 |
| The only study limitation cited in your discussion is the exclusion of one group of stakeholders. I encourage the authors to consider other possible limitations to conducting such a study. | We have added more limitations to our study in the discussion | Line 634- 648 |
| It would be essential to focus your discussion on the need for researchers to use co design approaches to design context-specific implementation strategies. Besides the findings drawn from the workshop participants, what have you learned as a researcher when applying a co-design approach? What advantages and disadvantages can be pointed out? | We have recommended that researchers focus on co-design approaches and provide lessons from our study. | Line 661- 664 and  649- 660 |
| What implications do your findings have from a research and policy perspective in Malawi? | We have added the implication of our research from a policy perspective in Malawi | Line 664-668 |
| **Editor comments** |  |  |
| Include a complete copy of PLOS’ questionnaire on inclusivity in global research | We have filled and included the form S1 Checklist |  |
| In the ethics statement in the methods, you have specified that verbal consent was obtained. Please provide additional details regarding how this consent was documented and witnessed and state whether the IRB approved this | We have added this: “The study was reviewed and approved by the College of Medicine Research & Ethics Committee (P.04/22/3607) and the Liverpool School of Tropical Medicine Research Ethics Committee (22-025). Participants provided informed verbal consent to attend the workshops, which involved training on the frameworks, validating the findings of Suwedi-Kapesa et al., and developing and refining the intervention guided by frameworks. We first sought written approval from the health and social services director to release HCW participants and non-governmental organisations, who gave us contacts to approach. Participants were given the invitation letter and study information. After two days, the researcher followed up by phone if they had questions and were interested in participating in the workshops. Interested participants opted in and two opted out and were sent the workshop materials and agenda by phone two weeks before the workshops. | Line 103-113 |
| Please amend your detailed Financial Disclosure statement | We received funding from the Commonwealth Scholarship Commission (CSC) grant number (MWCS-2020-286) and the Institutional Translation Partnership award (Wellcome) through the Policy Catalyst Fund—Malawi Liverpool Wellcome Trust (206545/Z/17/Z). LCSK received support from CSC. LSTM supported AC. ND, and AO. KUHeS supported ALNM; MLW supported DN; JHZ, EL and JN were supported by the Ministry of Health; and EGPAF supported AM. The policy catalyst fund supported the stakeholder engagement workshops. The funders had no role in study design, data collection and analysis, publication decisions, or manuscript preparation. The content does not represent the views of the funders. |  |
| Please confirm at this time whether or not your submission contains all raw data required to replicate the results of your study. Authors must share the “minimal data set” for their submission | Our submission contains all relevant raw data in a compressed folder |  |
| Please insert an Ethics Statement at the beginning of your Methods section, under a subheading 'Ethics Statement' | We have inserted the statement | Line 103 |
| 103Please provide an Author Summary. This should appear in your manuscript between the Abstract (if applicable) and the Introduction and should be 150–200 words long. | Conceptualisation, LCSK under the supervision of AO and ND. LCSK developed methodology and executed it with EL, JHZ, DN, and AM with supervision and guidance from AO, ND, AC, ALNM, JN and MS. Software LCSK. Formal analysis was conducted by LCSK with Validation from AO, ND, EL, JHZ, DN, AO and AM. Visualisation LCSK, Writing—original draft LCSK, Funding acquisition, LCSK. Project administration LCSK, ALNM, AC & MS, Resources LSCK. All authors reviewed and provided feedback on the analysis and approved the final manuscript. | After abstract |
| Please review your reference list to ensure that it is complete and correct | We have done this, and we are open to any pointers if any part has been missed |  |
